# Identification of common hub genes and construction of immune regulatory networks in aplastic anemia, myelodysplastic syndromes, and acute myeloid leukemia

**DOI:** 10.3389/fimmu.2025.1547289

**Published:** 2025-05-08

**Authors:** Mingliang Shan, Li Xu, Wenzhe Yang, Lili Sui, Ping Sun, Xiumei Zhuo, Shiguo Liu

**Affiliations:** ^1^ Medical Genetic Department, The Affiliated Hospital of Qingdao University, Qingdao, China; ^2^ Post - Doctoral Innovation Practice Base, Gaomi Maternity and Child Health Hospital, Gaomi, China; ^3^ School of Management, Shandong Second Medical University, Weifang, China; ^4^ College of Acupuncture and Massage, Shandong University of Traditional Chinese Medicine, Jinan, China

**Keywords:** aplastic anemia, myelodysplastic syndromes, acute myeloid leukemia, hub gene, bioinformatics, Mendelian randomization, plasmid

## Abstract

**Background:**

Aplastic anemia (AA), myelodysplastic syndromes (MDS), and acute myeloid leukemia (AML) exhibit complex pathogenic mechanisms and interrelated characteristics. We aimed to identify the common hub genes, establishing a foundation for preventing disease progression.

**Methods:**

We selected relevant datasets from the Gene Expression Omnibus(GEO) database for differential gene expression, gene set enrichment, and weighted gene co-expression network analyses to identify hub genes, and then validated them. Subsequent analyses included immune infiltration analysis, single-cell sequencing, and cell communication analysis. We performed Mendelian randomization to screen inflammatory factors and immune cells. We used RT-qPCR, Enzyme - Linked Immunosorbent Assay(ELISA), and cell proliferation assays to validate the identified hub genes, their relationship with cellular communication mediators and inflammatory factors, and their impact on cellular function.

**Results:**

POLG and MAP2K7 were identified as common hub genes, with low expression observed across AA, MDS, and AML. There were distinct immune differentials among these diseases, with an enhanced correlation between immune cells and hub genes as the disease progressed. Macrophage Migration Inhibitory Factor(MIF) emerged as a key mediator of cellular communication. We identified 20 regulatory pathways of immune cells and inflammatory factors across different disease stages. *In vitro* validation confirmed low expression of the hub genes, which were inversely correlated with MIF and inflammatory factors, though they showed no significant impact on cell proliferation or migration.

**Conclusions:**

POLG and MAP2K7 demonstrate crucial roles in the progression from AA to MDS and, ultimately, to AML. These genes regulate more than 20 immune regulatory pathways through MIF-mediated communication, thereby influencing disease progression.

## 1 Introduction

Within the expansive realm of hematological research, aplastic anemia (AA), myelodysplastic syndromes (MDS), and acute myeloid leukemia (AML) stand as major diseases that captivate the attention of researchers worldwide. These diseases exhibit intricate connections and distinctions in their pathogenesis, clinical manifestations, therapeutic responses, and prognosis (1).

AA, with an incidence of 2–3 cases per million people (2), is characterized as an immune-mediated bone marrow hematopoietic failure (3–5), where T lymphocytes mount an immune assault against hematopoietic stem/progenitor cells (6). Recently, the advent of immunomodulatory therapies has significantly refined treatment strategies for AA, markedly enhancing patient outcomes (7, 8). Nevertheless, the underlying pathogenesis remains incompletely elucidated (9–11), necessitating further investigation (3). Additionally, a large percentage of AA patients may progress to MDS (12). Long-term survivors of AA are at a high risk of developing MDS and AML following immunosuppressive therapy (IST) (13, 14). Approximately 15% to 20% would develop secondary MDS/AML within a decade of follow-up (15).

MDS, with an incidence of 3–4 per one hundred thousand (16), are heterogeneous clonal hematopoietic stem cell disorders (17), characterized by dysplastic blood cell development and varying degrees of cytopenias (18, 19). This disease is postulated to have significant immunological abnormalities (20), and patients often face a high risk of progression to AML, a process entwined with complex genetic alterations (21, 22). Progression rates from MDS to AML range from 5-15% in low-risk cases to 40-50% in high-risk cases (23). Furthermore, the lack of distinctive clinical features often results in delayed treatment, increasing the risk of AML transformation.

AML, with an incidence of 4.3 cases per one hundred thousand (24),an aggressive hematological malignancy, is closely associated with genetic and epigenetic alterations (25, 26), alongside marked immunological aberrations (27). Both immunophenotyping and immunotherapy have become essential components of AML management (25, 28, 29). High-throughput sequencing has greatly advanced the molecular classification of AML (30), providing support for the implementation of precision medicine (31, 32). However, the treatment faces various challenges, including drug resistance and high relapse rates (33), leading to increased treatment complexity and mortality rates (25). Preventing disease progression to this advanced stage is therefore critical for reducing treatment difficulty and improving prognosis.

Immune abnormality is the common feature of the above three diseases, and they share certain similarities in their transformation and immunological features. Thus, we hypothesized that they have common abnormally expressed genes, which play vital roles in immunological regulation throughout disease progression.

This study aimed to identify common hub genes among the three diseases, assess their immune characteristics, and explore their interrelationships. We identified key mediators through cell communication analysis and constructed immune regulatory networks using Mendelian Randomization (MR) analysis. *In vitro* validation confirmed the reliability of common hub genes and communication mediators, shedding light on their roles in immune pathways and their impact on cellular function. The verification of the immune regulatory network and related functions of the hub genes is conducive to clarifying their importance in preventing the progression of the three diseases during the disease progression, as well as mapping the different immune networks regulated by them in the three diseases.

## 2 Materials and methods

### 2.1 Bioinformatics analysis

#### 2.1.1 Data sources

The datasets were sourced from the GEO database (https://www.ncbi.nlm.nih.gov/geo/) (34), specifically GSE15061 (404 AML samples and 328 MDS samples) and GSE3807 (8 AA samples). Additional data was retrieved from the GTEX dataset (11 datasets) on the UCSC Xena platform (http://xena.ucsc.edu/), selecting 70 samples of normal bone marrow. The samples of the three diseases were respectively combined with 70 normal samples, and batch correction was performed on each set of combined data to eliminate the batch effects. GSE185381 was selected as the single-cell sequencing analysis dataset for AML and normal cells. Detailed information for each dataset is provided in **Supplementary Table S1**-datasets.

#### 2.1.2 Differential gene expression analysis and functional enrichment analysis

A threshold of P < 0.05 and |log2 fold change (FC)| > 1(a difference of more than twice) was set. We took the intersection of upregulated and downregulated genes for each disease, respectively, and subjected them to Gene Ontology (GO) and Kyoto Encyclopedia of Genes and Genomes (KEGG) enrichment analyses. Intersection genes were further analyzed using TRRUST (https://www.grnpedia.org/trrust/) for transcription factor enrichment, and a regulatory network was subsequently constructed.

#### 2.1.3 Weighted gene co-expression network analysis

We clustered genes from each disease and excluded outliers. The fitting index and average connectivity were determined by calculating the optimal power value and were used to construct the optimal scale-free network. We draw the scale-free topology to validate network construction success. A distance matrix was derived, and gene clustering was performed. Dynamic module identification was carried out with the criteria that the number of genes in each module should be ≥ 30. Similar modules were clustered and merged, and heatmaps between modules and clinical traits were plotted to identify significant modules. Gene significance (GS) > 0.5 and module membership (MM) > 0.8 were set as thresholds to identify key module gene sets.

#### 2.1.4 Identification and validation of common hub genes

We intersected the upregulated and downregulated genes from the key modules identified in the WGCNA with the upregulated and downregulated genes from each disease. The resulting key genes underwent validation through Least Absolute Shrinkage and Selection Operator (LASSO) regression analysis. We draw boxplots for each disease to assess the differential expression of common hub genes between groups. Additionally, receiver operating characteristic (ROC) curves were generated for further validation.

#### 2.1.5 Expression of common hub genes and gene set enrichment analysis of synergistic gene

We visualized the expression profiles of common hub genes across various tissues, differentiated by gender. The synergistic gene set interacting with the hub genes was extracted from a merged dataset of the three diseases, followed by GSEA analysis to identify enriched pathways.

#### 2.1.6 Immune infiltration analysis

Immune-related GSEA analysis was performed for each disease, with P < 0.05 considered statistically significant and |log2 FC| set to > 1. Additionally, the single sample GSEA (ssGSEA) analysis was conducted to assess changes in immune infiltration during disease transformation and its potential role.

#### 2.1.7 Single-cell sequencing and cell communication analysis

Based on the hypothesis that the transformation from normal cells to AA, then to MDS, and ultimately to AML represents a dynamic process, we utilized available single-cell datasets for normal cells and AML, which represent the initial and terminal stages, respectively. Due to the lack of complete single-cell datasets for AA and MDS, we used data from existing literature to supplement the analysis and form a complete single-cell study (35, 36). Therefore, we could infer similarities and differences between the three diseases at the single-cell level.

We processed single-cell transcriptomic data using the package “Seurat”, following established protocols (37). Single-cell sequencing datasets for AML and normal samples were selected from GSE185381, with filtering criteria including gene count > 50 and mitochondrial percentage < 5%. The 1500 genes with the highest variation coefficients were selected for principal component analysis (PCA) to reduce dimension. The top 20 most significant PCA components were selected for t-distributed stochastic neighbor embedding (t-SNE) clustering analysis and cell annotation. The expression of common hub genes in various cell types was visualized.

Based on the single-cell sequencing results, cell communication analysis was performed and visualized. A ligand-receptor interaction map was constructed, filtering out communications involving fewer than 10 cells. We also generated graphs of the number and strength of interactions. A bubble plot of receptor-ligand interactions was created according to cell types, highlighting core pathways that play critical roles in both normal and AML groups.

### 2.2 MR analysis

The MR analysis was conducted following the TROBE-MR-checklist (38, 39), utilizing the package “TwosampleMR”. All data used in this study were derived from publicly accessible databases, and the original studies had been ethically approved.

#### 2.2.1 Data sources

Data on AA were sourced from the IEU database (https://gwas.mrcieu.ac.uk/) (GWAS ID: ebi-a-GCST90018794), comprising 473,500 samples and 24,192,378 SNPs. Data for AML, MDS, immune cells, and inflammatory factors were retrieved from the EBI GWAS Catalog (https://www.ebi.ac.uk/gwas/). Accession numbers of AML and MDS were GCST90435652 and GCST9004394, respectively. Immune cell accession numbers ranged from GCST90274758 to GCST90274848, covering 728 immune cell types and their corresponding GWAS IDs (**Supplementary Table S1**-immune cell). Inflammatory factors had accession numbers from GCST90274758 to GCST9027484, encompassing 90 immune cell types with IDs listed in the EBI GWAS Catalog (**Supplementary Table S1**-inflammatory factors). All samples were drawn from European populations.

#### 2.2.2 Instrumental variable selection

IV needs to satisfy three assumptions: relevance, independence, and exclusion restriction. All IVs must undergo linkage disequilibrium (LD) test, heterogeneity test, and pleiotropy test. Further screening involved: (1) IVs should adhere to genome-wide significance thresholds (P < 5.0 × 10^-8). If significant SNPs were unavailable, SNPs with P < 5×10^-6 were considered candidates; (2) LD assessed using European samples from the 1000 Genomes Projects was treated as the reference. The SNPs with the lowest P-values at R^2^ = 0.001 (clumping window size = 10,000 kb) were considered; (3) SNPs with a minor allele frequency (MAF) ≤ 0.01 were excluded; (4) Palindromic SNPs (A/T with ambiguous allele frequencies or G/C polymorphisms) were excluded when harmonizing exposure and outcome data. For each SNP included in the analysis, the following methods were used to calculate R^2^ and F values for efficiency evaluation according to the data situation: R^2^ = 2*EAF*(1-EAF)*β^2^ or R^2^ = β^2^/(β^2^+SE^2^*N), and F = R^2^(N-2)/(1-R^2^), ensuring F ≥ 10.

#### 2.2.3 Batch screening

Following established protocols (40), we conducted MR analyses as follows: (1) Firstly, the collected 728 types of immune cells and 90 inflammatory factors were used as exposures, with the three diseases as outcomes, identifying positive immune cells and inflammatory factors; (2) Positive immune cells and inflammatory factors were further analyzed to identify bidirectional associations, denoted as double-positive inflammatory factors and double-positive immune cells; (3) Factors served as the outcomes in step (2) were used as exposure, with the three diseases as outcomes in subsequent MR analysis, identifying triple-positive immune Cells and triple-positive inflammatory factors.

#### 2.2.4 Mediation analysis

Based on the relationships of triple-positive immune cells and inflammatory factors, we determined two components of the mediated MR analyses: triple-positive immune cells act on triple-positive inflammatory factors to cause diseases and triple-positive immune cells act on triple-positive inflammatory factors to cause diseases. Each batch of analysis included four steps: (1) The total effect from exposure to outcome (beta_all) was evaluated; (2) Potential reverse causation was assessed; (3) The effect of exposure on mediator (beta1) was calculated; (4) The effect of mediator to outcome (beta2) was calculated, utilizing distinct SNPs from step three. The mediation effect was calculated as beta12 = beta1*beta2. The total effect can be decomposed into the direct effect of exposure on the outcome (beta_dir) and the indirect effect of exposure mediated through the mediator (beta12). The mediation proportion (Z) was calculated by dividing the indirect effect by the total effect. We used the delta methods for 95% confidence intervals (CI). The IVW and MR-Egger methods were applied to determine causality (41, 42). IVW combines the causal effects of each SNP through meta-analysis, and the premise is that all SNPs are valid IVs, so this method needs to be used after excluding pleiotropy. When IVW and MR-Egger results cannot satisfy P < 0.05 at the same time, the IVW results would be used as the final judgment basis. When neither IVW nor MR-Egger results satisfy P < 0.05, the results from the decomposition steps of IVW and MR-Egger can be examined to determine if they meet the P < 0.05 threshold. If both methods show P < 0.05, the direction of each step is then combined to define the overall effect direction.

#### 2.2.5 Sensitivity analysis

Cochran’s Q statistic was utilized to measure the heterogeneity of IVs, with P > 0.05 indicating no heterogeneity, calculated using the mr_heterogeneity function. A random effects model was applied when significant heterogeneity was detected among SNPs, otherwise, a fixed effects model would be used. Additionally, a “leave-one-out” analysis was performed to identify potential outlier SNPs. Pleiotropy was evaluated based on the intercept calculated by MR-Egger regression using mr_pleiotropy_test.

### 2.3 Experimental validation

#### 2.3.1 Cell sources

This study was approved by the local hospital ethics committee (No. 20230309-03), complying with the Declaration of Helsinki. Written informed consents were obtained from both the donors and their parents for the use of human tissues, body fluids, or cell lines.

The AA cells were derived from the bone marrow sample of a child with severe AA. The MDS cells were the MDS-L cell line preserved in the laboratory, and STR identification had been carried out before the experiment (see attached **Supplementary Figure S1**). The AML cells were the KG-1a cell line (CELLCOOK, Guangzhou, Guangdong, China), and STR verification was also conducted for them (see attached **Supplementary Figure S2**). The normal control cells were the human umbilical cord blood stem cells from an individual whose umbilical cord blood stem cells were stored in our hospital (protocol number: ZTQX0470663).

Cells were cultured in Dulbecco modified Eagle medium (DMEM) (HyClone; GE Healthcare Life Sciences, Logan, UT, USA) supplemented with 10% fetal bovine serum (FBS) (opcel, Shanghai, China), and incubated at 37°C under a humid 5% CO2 atmosphere. Cells were plated in 6-well plates at a density of 1 × 10^5^ cells per well, and these in the logarithmic growth phase were used for subsequent experiments.

#### 2.3.2 Plasmid construction

The pUC19 plasmid (Solarbio, Beijing, China) was selected as the expression vector (**Supplementary Figure S3**), and vectors were digested with HindIII (LMAI Bio, Shanghai, China) and NdeI (KALANG, Shanghai, China). Gene sequences for Homo sapiens POLG and MAP2K7 were retrieved from NCBI, and primers were designed accordingly (**Supplementary Table S1**-Step 1). The DNA fragment was ligated using overlap extension PCR (**Supplementary Figure S4**). The ligation product was then transferred into competent Escherichia coli DH5α (Wenzhou KeMiao Biotechnology Co., Ltd., Wenzhou, Zhejiang, China), which was revived on blasticidin-free medium and subsequently grown on kanamycin (Beijing Ita Biotechnology Co., Ltd., Beijing, China) plates. Single colonies were randomly selected and subjected to plasmid transfection. We performed qPCR to confirm the successful construction of plasmid and expression of the hub genes in the target cells.

#### 2.3.3 Experimental grouping and plasmid transfection

The cells were divided into the following groups: Normal (human umbilical cord blood stem cells), AA Control (AA cells), AA Experimental (AA cells transfected with plasmids), MDS Control (MDS-L cells), MDS Experimental (MDS-L cells transfected with plasmids), AML Control (KG-1a cells), and AML Experimental (KG-1a cells transfected with plasmids). Each group was cultured in 4500 µL of 20% DMEM (absin, Shanghai, China) supplemented with 500 µL FBS (opcel) and 200 µL of penicillin-streptomycin (P/S) (absin). Cells in the logarithmic growth phase were transfected using Lipofectamine 2000 (Invitrogen, Hangzhou, Zhejiang, China).Then, gently mix the diluted plasmid DNA with the Lipofectamine 2000 reagent, and incubate them at room temperature for 15–20 minutes to allow the formation of transfection complexes. Add the transfection complexes drop - by - drop into the culture vessel containing the cells. Gently shake the culture vessel to ensure the complexes are evenly distributed on the cell surface. Then, return the cell culture vessel to the cell incubator and continue the cultivation for 6 hours. Each group was set up with three replicate wells.

#### 2.3.4 RT-qPCR

Total RNA was extracted using Trizol reagent (Life Technologies, Grand Island, NY, USA) and quantified using spectrophotometric readings at 260/280 nm. The RNA was resuspended in RNase-free water and treated with DNase I (Life Technologies) to remove potential DNA contamination. Reverse transcription was carried out using the ReverTra Ace qPCR RT Kit (TOYOBO, Japan) for 1 µg total RNA of each sample. First-strand cDNA was synthesized in 20 μL of reaction mixture. cDNA was stored at −20°C until further use.

Specific primer sequences were inserted (**Supplementary Table S1**-Step 2). The expression levels of target genes were quantified using the 2^−ΔΔCT method, with GAPDH as the internal control. Tianlong Gentier 96E PCR analysis system (TIANLONG, China) and LEPU fluorescence quantitative PCR system (LEPU, China) were used for analyses.

#### 2.3.5 Expression of MIF and hub genes

Expression of MIF and hub genes was compared between the normal and control groups for each disease using RT-qPCR.Considering that the data scale of Group AA in the bioinformatics section is relatively small, in order to enhance the robustness of the differential expression analysis for this group, Group AA was verified intensively by dividing it into two independent cohorts.

#### 2.3.6 Impact of high-expression hub genes on MIF and inflammatory factors expression, and cellular function

After culturing the normal and experimental groups of each disease for 72 hours, RT-qPCR was performed to assess the expression of hub genes and MIF following plasmid transfection. All subgroups were selected, and the medium was then replaced with human serum, which was obtained from the remaining serum after serum transfusion of neonates with coagulation disorders in our hospital. Each group included three replicate wells. With reference to a previous study (43), cells were cultured for 72 hours, and RT-qPCR was performed to evaluate the expression of proliferating cell nuclear antigen (PCNA), B-cell lymphoma 2 (Bcl-2), and Matrix metalloproteinase-2 (MMP-2) in each group following the induced overexpression of hub genes.

PCNA plays a crucial role in cell proliferation, primarily expressed in the nucleus (44). PCNA expression significantly increases during the S phase of the cell cycle, where it functions as a DNA polymerase auxiliary factor, promoting DNA synthesis and replication (45). Bcl-2, an anti-apoptotic protein located on the mitochondrial membrane, regulates cell survival by inhibiting apoptosis through reducing cytochrome C release and other pathways (46, 47). The balance of its family proteins determines whether cells will undergo apoptosis. MMP-2, a member of the protease family, is secreted by cells and is capable of degrading extracellular matrix components (48). In physiological processes, MMP-2 is involved in tissue remodeling, and in pathological conditions, it is associated with tumor invasion, metastasis, and various diseases. Inhibiting MMP-2 activity could be a therapeutic strategy for several diseases (49).

The Normal, AA Control, MDS Control, AML Control, AA Experimental, MDS Experimental, and AML Experimental groups were used. From the 20 immune regulatory pathways identified through MR, MIF and inflammatory factors relevant to each disease were selected for analysis. To measure the cytokine levels, a double-antibody sandwich enzyme-linked immunosorbent assay (ELISA) method was performed on the collected medium. Centrifuge samples of the culture mediums immediately at 4000 × g for 5 minutes to collect the conditioned supernatant, and store at −80°C until use. Cytokine concentrations, including MIF, LIF (AA), HGF (MDS), IL-20 (MDS), TWEAK (AA), TSLP (AML), and CCL19 (MDS), were measured using ELISA kits according to the manufacturer’s protocol (for reagent information and the manual, please refer to **Supplementary Figure S5**). Standard curves for each cytokine were constructed using standard solutions, and cytokine concentrations in the samples were calculated by comparing the optical density (OD) values of the samples to the standard curves (**Supplementary Table S2**). The assays were constructed using a BioTek Epoch full-wavelength microplate reader (Epoch, USA).The ELISA experimental procedure underwent enhanced validation across two separate independent cohorts.

In parallel, cell proliferation was measured using the MTT assay. Cells from all groups were cultured in human serum for 72 hours. Cell proliferation was assessed using the CellTiter 500T assay kit (Wanlei Biotechnology, Shanghai, China) and a FlexA-200 full-wavelength microplate reader (Aosheng, Hangzhou, China). In each group, three replicate wells were set, with 5000 cells seeded per well in 96-well plates. The MTT concentration was set at 0.2 mg/mL, and the absorbance was measured at 570 nm.The MTT experimental process was strengthened and validated in two independent cohorts.

### 2.4 Statistical analysis

Statistical analyses were performed using SPSS 25.0 and R 4.4.1. P<0.05 was considered statistically significant. Comparisons between two groups were conducted using t-tests, while pairwise comparisons between multiple groups were performed using LSD-t tests.

## 3 Results

### 3.1 Bioinformatics analysis

#### 3.1.1 Transcriptomic characteristics

**Figure 1:**
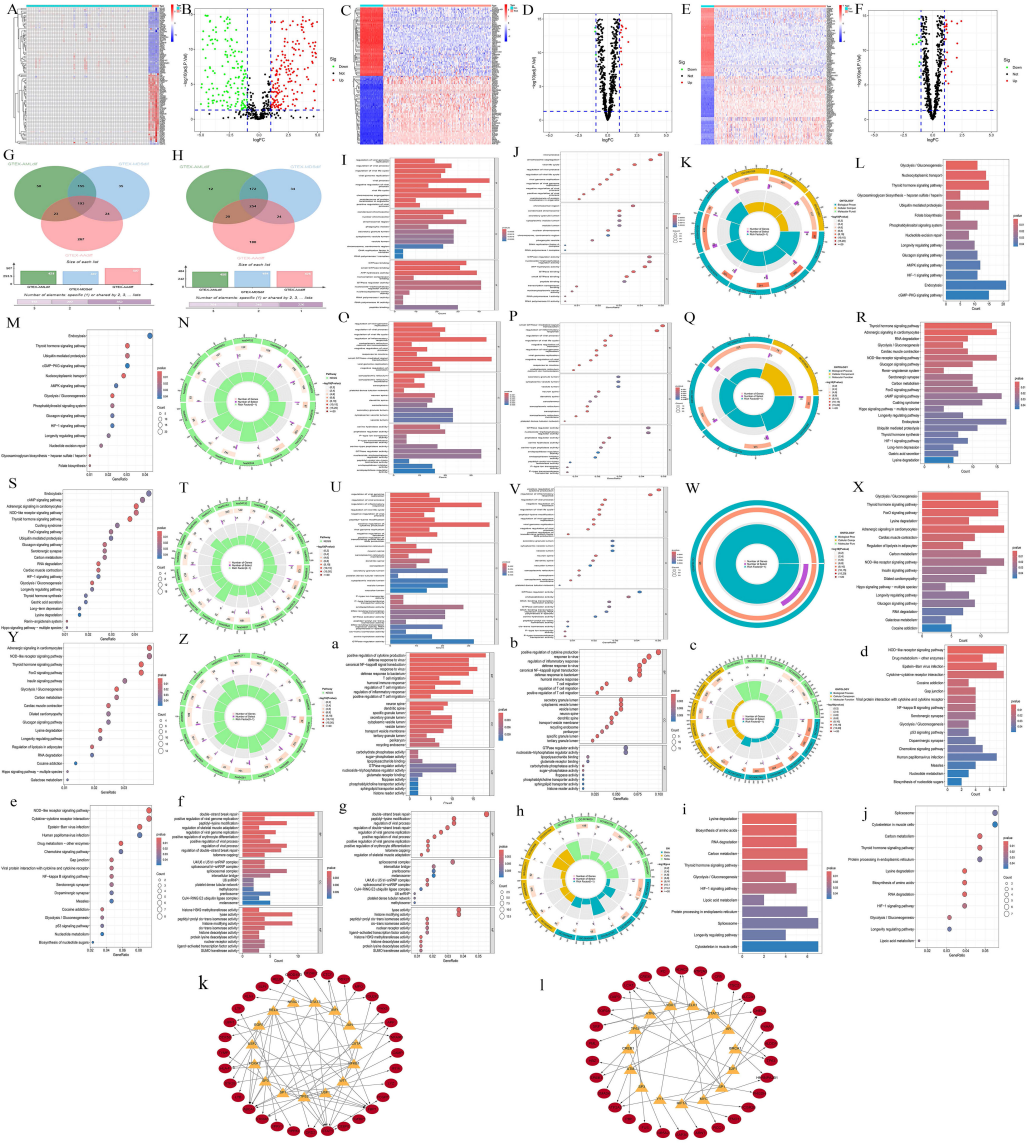
Transcriptomics preliminary analysis. **(A, C, E)** Heatmaps illustrating the DEGs in disease samples and normal samples. The abscissa represents different samples. Blue represents the normal group and red represents the disease group (A, AA; C, MDS; E, AML). The ordinate represents genes. High expression is red and low expression is deep blue. **(B, D, F)** Volcano plots of DEGs in disease vs normal samples. Red, green, and black points indicate genes that are upregulated, downregulated, or have no significant difference in the disease group compared with the normal group (B, AA; D, MDS; F, AML). **(G, H)** Venn diagrams displaying the commonly upregulated and downregulated gene sets. Red represents the AA group, blue represents the MDS group, and green represents the AML group. **(I-K, O-Q, U-W, a-c, f-h)** GO enrichment analyses. **(L-N, R-T, X-Z, d-e, i-j)** KEGG enrichment analyses. For AA, I-K; L-N, MDS, O-Q; R-T, and AML, U-W; X-Z, the gene sets upregulated in all three diseases are: a-c, d-e, and the gene sets downregulated in all three diseases are: f-h, i-j. For each type, results are presented as bar charts, bubble charts, and circle plots. In bar charts, redder means more significant difference and bluer means less. Bar length shows the number of enriched genes. In bubble charts, the same color - significance rule applies. The size of the bubbles represents the number of enriched genes. The outermost circle represents the GO IDs, and the next inner circle represents the number of enriched genes. The following inner circle represents the number of differentially expressed genes, and the innermost circle represents gene proportions. The color represents the second circle from outside to inside. The redder the color, the more significant the differential gene enrichment is.

A total of 985 differentially expressed genes (DEGs) were identified between AA and normal samples, comprising 507 upregulated genes (log2FC > 1) and 478 downregulated genes (log2FC < −1) (**Figures 1A, B**). Similarly, between MDS and normal samples, 891 DEGs were identified, with 407 genes upregulated and 484 genes downregulated (**Figures 1C, D**). In AML and normal samples, 879 DEGs were identified, including 421 upregulated genes and 458 downregulated genes (**Figures 1E, F**). By intersecting the upregulated and downregulated gene sets across the three diseases, we identified 193 commonly upregulated genes in all three conditions (**Figure 1G**), and 254 commonly downregulated genes (**Figure 1H**). GO enrichment results indicated that all diseases were primarily associated with viral infections (**Figures 1I-K, O-Q, U-W**), while KEGG enrichment showed a link to endocrine and metabolism (**Figures 1L-N, R-T, X-Z**). Among the common upregulated and downregulated genes, the GO enrichment results showed that the upregulated genes were closely associated with viruses, inflammation, and T-cells (**Figures 1A-C**), while KEGG analysis showed a link to pathogen infection and the degradation of biological compounds (**Figures 1D, E**). The downregulated genes set was associated with viruses (GO) and endocrine or metabolism (KEGG) (**Figures 1F-J**). Further transcription factor enrichment analysis indicated that 15 transcription factors were enriched within the common upregulated and downregulated gene sets (**Tables S3**-up-regulate, S3-Down-regulated), respectively, with their regulatory interactions depicted in **Supplementary Figures S1A, B**.

#### 3.1.2 Hub genes selection

**Figure 2:**
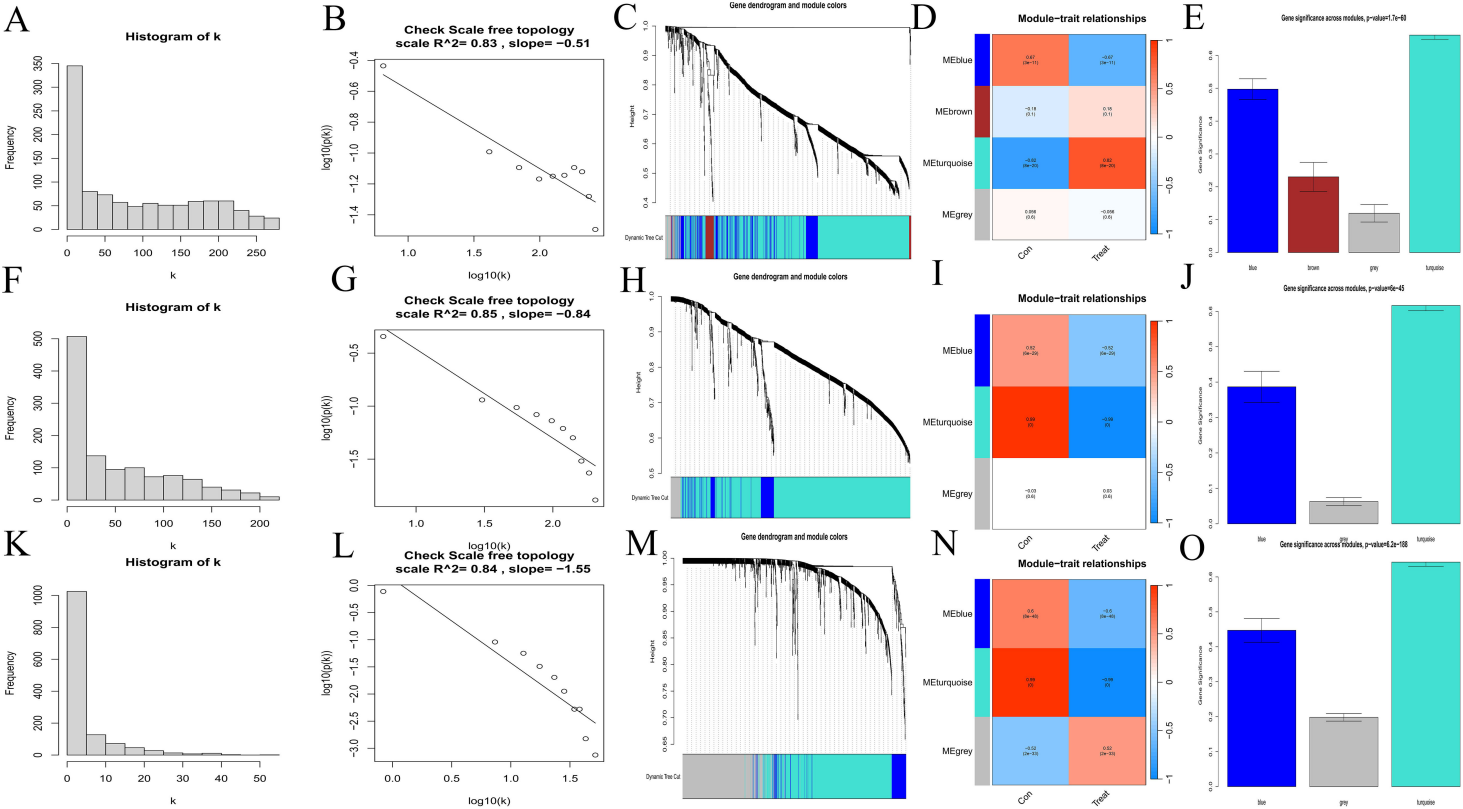
Selection of hub genes. **(A, B, F, G, K, L)** Scale-free network topology plots. AA, A-B; MDS, F-G; AML, K-L. **(C, H, M)** Merged weighted gene co-expression networks, AA, C; MDS, H; AML, M. **(D, I, N)** Heatmaps illustrating the correlation between modules and clinical traits. Red represents a positive correlation and blue represents a negative correlation. AA, D; MDS, I; AML, N. **(E, J, O)** Gene importance plots. The abscissa represents module names, and the ordinate represents gene importance. AA, E; MDS, J; AML, O.

Scale-free network topology analysis for each disease revealed that the correlation coefficients were all greater than 0.8 (AA = 0.83, MDS = 0.85, AML = 0.84), validating the power values chosen for constructing the scale-free network (AA: **Figures 2A, B**, MDS: **Figures 2F, G**, AML: **Figures 2K, L**). Then we yielded the WGCNA module results for each disease (AA: **Figure 2C**, MDS: **Figure 2H**, AML: **Figure 2M**). By correlating these modules with clinical traits (AA: **Figure 2D**, MDS: **Figure 2I**, AML: **Figure 2N**) and conducting gene importance analysis (AA: **Figure 2E**, MDS: **Figure 2J**, AML: **Figure 2O**), we identified the dominant modules for each disease (turquoise for AA, blue for MDS, and blue for AML).

#### 3.1.3 Identification and validation of common hub genes

**Figure 3:**
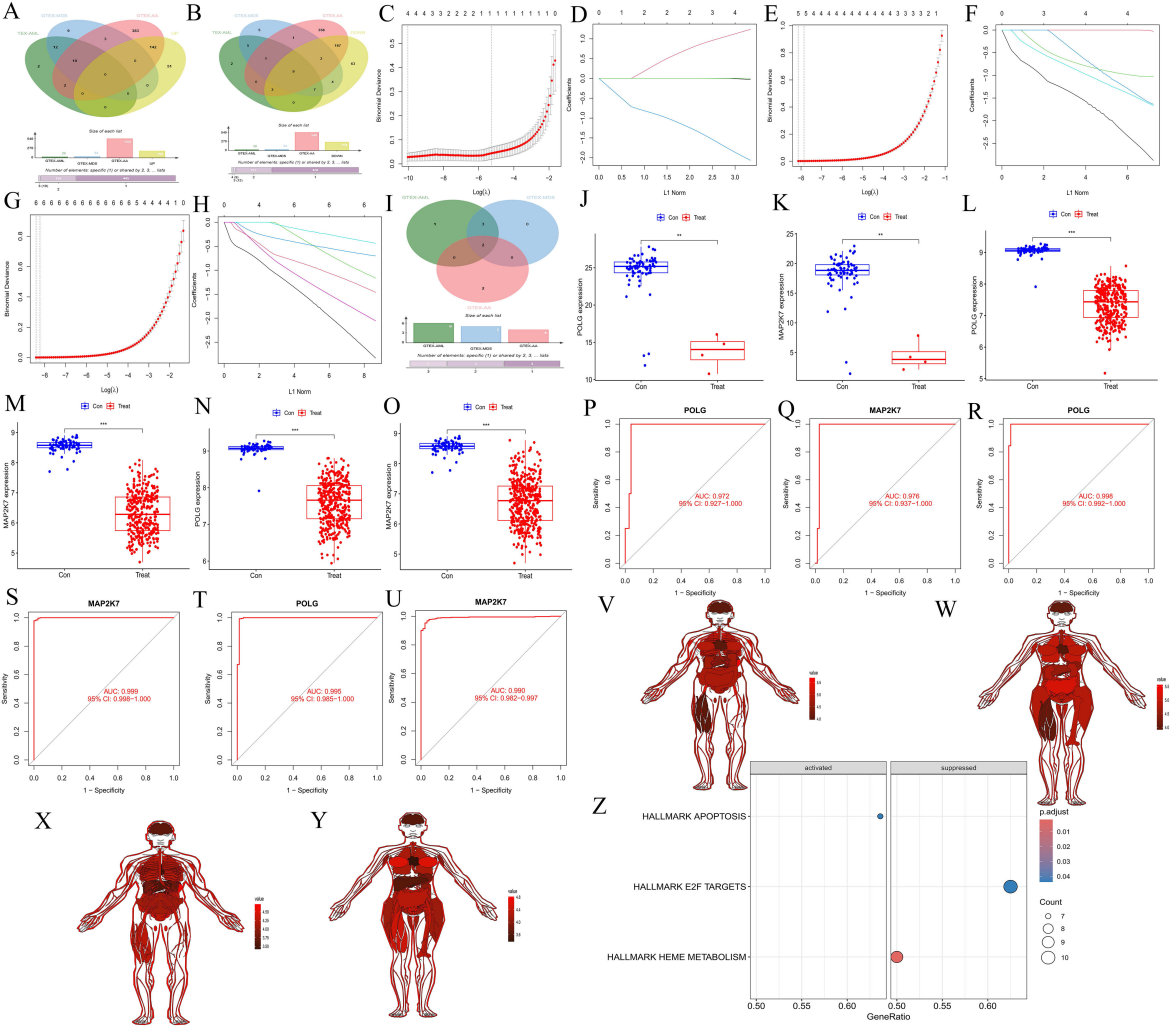
Screening and validation of common hub genes, their expression across different tissues, and GSEA analysis of synergistic genes. **(A, B)** Venn diagrams showing the intersection of upregulated and downregulated genes from key modules in WGCNA with upregulated and downregulated genes in each disease. **(A)** represents the upregulated gene set, and **(B)** represents the downregulated gene set. Red represents the AA group, blue represents the MDS group, green represents the AML group, and yellow represents the gene set in the WGCNA key module. **(C-D, E, F, G, H)** Lasso regression cross-validation plots for the three diseases, AA, C-D; MDS, E-F; AML, G-H. The abscissa represents Log(λ) values, and the ordinate represents cross-validation errors. **(I)** Venn diagram showing the intersection of genes after Lasso validation. Red represents the AA group, blue represents the MDS group, and green represents the AML group. **(J-O)** Box plots of differential expression for common hub genes. AA, J-K; MDS, L-M; AML, N-O. **(P-Q, R-S, T-U)** ROC validation curves. AA: P-Q, MDS: R-S; AML, T-U. The abscissa represents the false positive rate (1 - specificity), and the ordinate represents the true positive rate (sensitivity). **(V-Y)** Expression of common hub genes across normal tissues in the body. POLG, males **(V)** and females **(W)**. MAP2K7, males **(X)** and females **(Y)**. **(Z)** GSEA analysis of immune-related synergistic genes for common hub genes, where only POLG was enriched.

Intersecting the upregulated and downregulated genes from the key WGCNA modules with the DEGs for each disease, we identified 10 common upregulated genes (ENO1, MORF4L2, RHEB, POLG, DCAF13, MAP2K7, PDIA3, FNBP4, RPS24, and TSPAN3) (**Figure 3A**), and 9 common downregulated genes (ENO1, MORF4L2, RHEB, POLG, DCAF13, MAP2K7, PDIA3, RPS24, and TSPAN3) (**Figure 3B**). Further Lasso regression analysis of these genes (AA: **Figures 3C, D**, MDS: **Figures 3E, F**, AML: **Figures 3G, H**) narrowed the scope to POLG and MAP2K7, which were downregulated across all diseases (**Figure 3I**). These genes were further validated using boxplots (AA: **Figures 3J, K**, MDS: **Figures 3L, M**, AML: **Figures 3N, O**) and ROC curves (AA: **Figures 3P, Q**, MDS: **Figures 3R, S**, AML: **Figures 3T, U**), confirming their reliability.

#### 3.1.4 Expression of common hub genes in normal tissues and GSEA analysis of synergy genes

The common hub genes were found to be highly expressed in most normal tissues (POLG: **Figure 3V** for males, **Figure 3W** for females; MAP2K7: **Figure 3X** for males, **Figure 3Y** for females). GSEA analysis of the synergy genes revealed that only POLG was successfully enriched, activating the APOPTOSIS pathway and inhibiting the HEME METABOLISM and E2F TARGETS pathways (**Figure 3Z**). These findings suggested that POLG is associated with cell apoptosis and heme metabolism, which aligns with the disease characteristics.

#### 3.1.5 Immune infiltration

**Figure 4:**
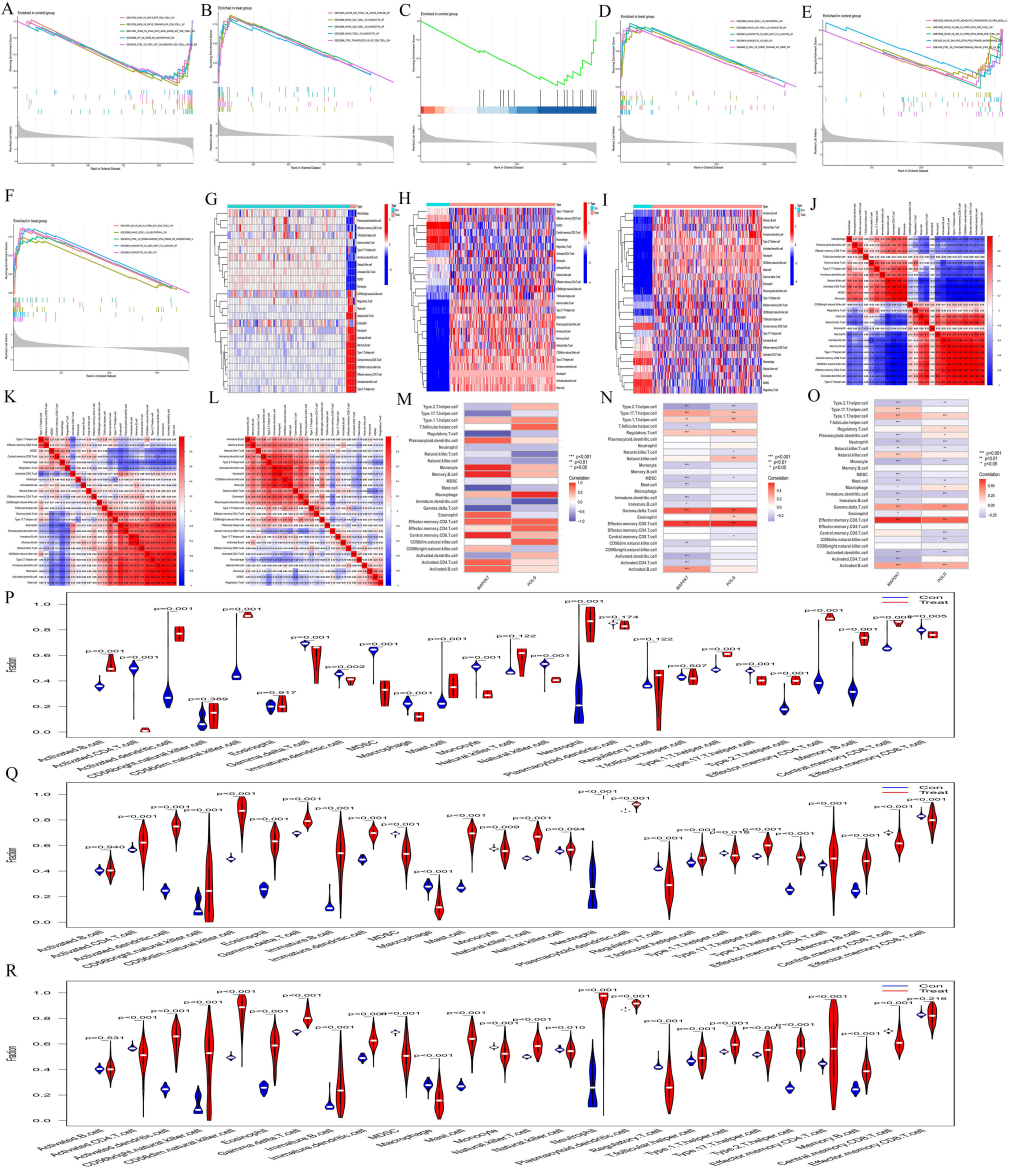
Immune infiltration analysis. **(A-F)** Immune-related GSEA enrichment analyses. In AA, **(A)** represents the normal group, and **(B)** represents the AA group. In MDS, **(C)** represents the normal group, and **(D)** represents the MDS group. In AML, **(E)** represents the normal group, and **(F)** represents the AML group. **(G-I)** ssGSEA immune infiltration analysis heatmaps. AA, G; MDS, H; and AML, **(I)** The abscissa represents different samples. Blue represents the normal group (Con) and red represents the disease group (Treat) The ordinate represents different immune cell types. **(J-L)** Correlation heatmaps between ssGSEA immune cells. AA, J; MDS, K; and AML, L. The redder the color, the higher the positive correlation between the two, and the bluer the color, the higher the negative correlation. **(M-O)** ssGSEA gene-immune cell correlation heatmaps. AA, M; MDS, N; and AML, O. The abscissa represents gene names, and the ordinate represents immune cell types. The redder the color, the higher the positive correlation between the gene and immune cell type, and the bluer the color, the higher the negative correlation. **(P-R)** Violin plots of ssGSEA immune cells. AA, P; MDS, Q; and AML, R. The abscissa represents immune cell types, and the ordinate represents the percentage of immune cells. Blue represents the normal group, red represents the disease groups, and p indicates the p-value of the difference between the two groups.

The immune-related KEGG pathway enrichment revealed distinct immune cell types enriched across the three diseases (AA: **Figures 4A, B**, MDS: **Figures 4C, D**, AML: **Figures 4E, F**), highlighting the immunological heterogeneity. ssGSEA immune infiltration analysis (AA: **Figure 4G**, MDS: **Figure 4H**, AML: **Figure 4I**) showed significant differences in immune cell content and composition between normal and diseased samples, suggesting immune alterations. Correlation heatmaps (AA: **Figure 4J**, MDS: **Figure 4K**, AML: **Figure 4L**) further illustrated the connections between immune cells. Immune cell correlation heatmaps (AA: **Figure 4M**, MDS: **Figure 4N**, AML: **Figure 4O**) indicated that POLG and MAP2K7 play increasingly important roles throughout the progression from AA to MDS and AML, with the associated immune cell numbers and intensities becoming more pronounced. The violin plots showed that most immune cells exhibited significant differences between the control and the experimental group (AA: **Figure 4P**, MDS: **Figure 4Q**, AML: **Figure 4R**), indicating that all three diseases had immune differences compared to normal cells.

#### 3.1.6 Single-cell sequencing and cell communication analysis

**Figure 5:**
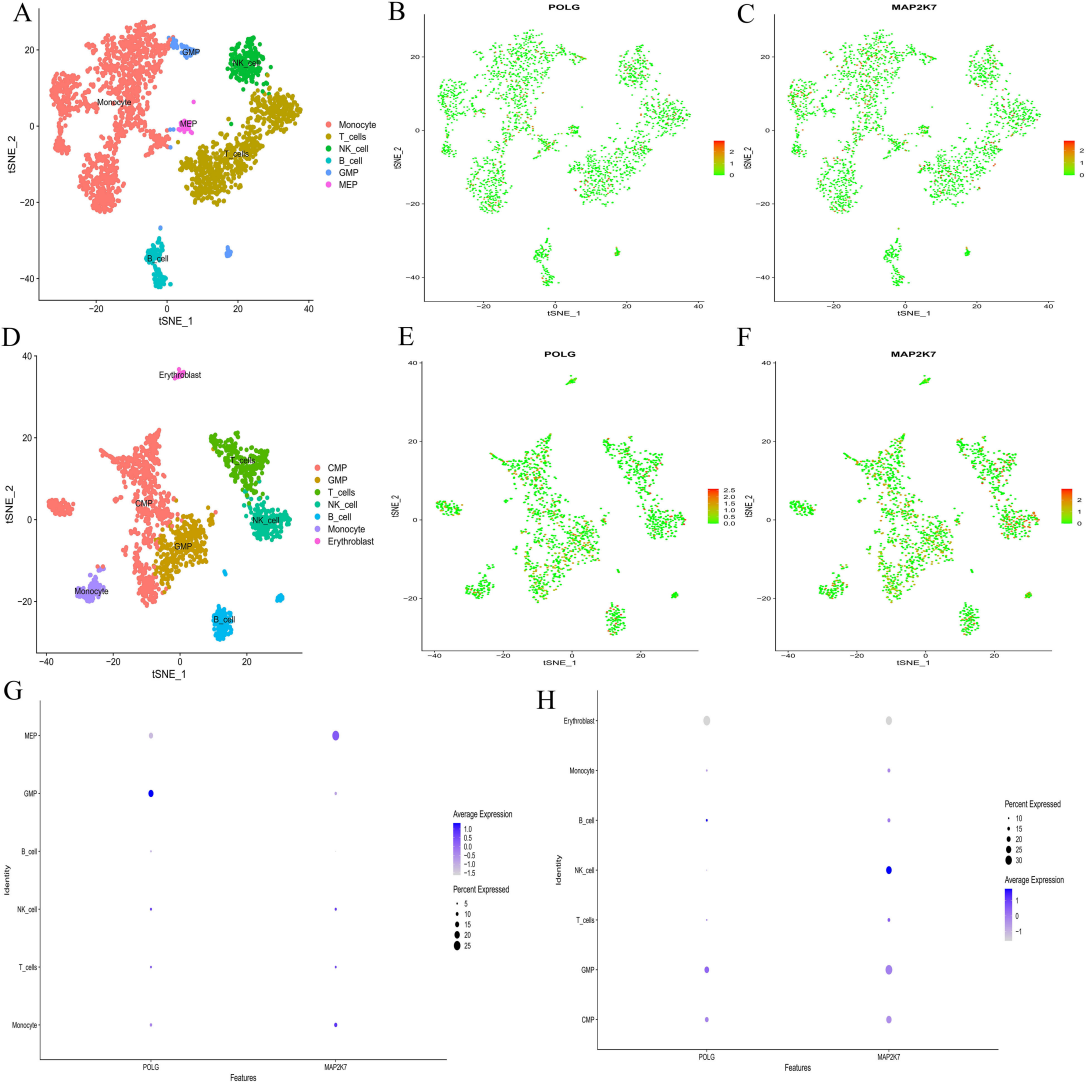
Single-cell sequencing analysis. **(A, D)** t-SNE clustering plots after dimension reduction by PCA. A, the normal group; D, the AML group. Different colors represent different clustering types. **(B-C, E-F)** Distribution of common hub genes in cells from the normal and AML groups. In the normal group, POLG is shown in B, and MAP2K7 in **(C)** In the AML group, POLG is shown in E, and MAP2K7 in **(F)** The redder the color, the higher the expression level, and the bluer the color, the lower the expression level. **(G, H)** Bubble plots for common hub genes. G, the normal group; H, the AML group. The abscissa represents different gene types, and the ordinate represents different immune cell types. The bubble size represents the proportion of gene expression, with bluer bubbles indicating higher expression levels.

Single-cell sequencing analysis revealed that the t-SNE clustering of the normal group identified 11 clusters, which were distributed across 6 cell types, including Monocytes, T-cell, NK-cell, B-cell, granulocyte‐macrophage progenitor(GMP), and Megakaryocyte - Erythrocyte Progenitor(MEP) (**Figure 5A**). The AML group identified 10 clusters, which were distributed across 7 cell types, including Common Myeloid Progenitor(CMP), GMP, T-cell, NK-cell, B-cell, Monocyte, and Erythroblast (**Figure 5D**). The main cellular compositions of the normal group (**Figures 5B, C**) and the AML group (**Figures 5E, F**) differed. Common hub genes were expressed to some extent in cells of all clusters, but the numbers were relatively low (**Figure 5G**). In the normal group, POLG was highly expressed and occupied a relatively large proportion in GMP, while it was lowly expressed and occupied a relatively large proportion in MEP. MAP2K7 was highly expressed and occupied a relatively large proportion in MEP, while it was lowly expressed but occupied a relatively large proportion in GMP (**Figure 5H**). POLG was lowly expressed and occupied a relatively large proportion in Erythroblast, while it was highly expressed and occupied a relatively large proportion in GMP. MAP2K7 was lowly expressed and occupied a relatively large proportion in Erythroblast, while it was highly expressed and occupied a relatively large proportion in both GMP and NK-cell.

**Figure 6:**
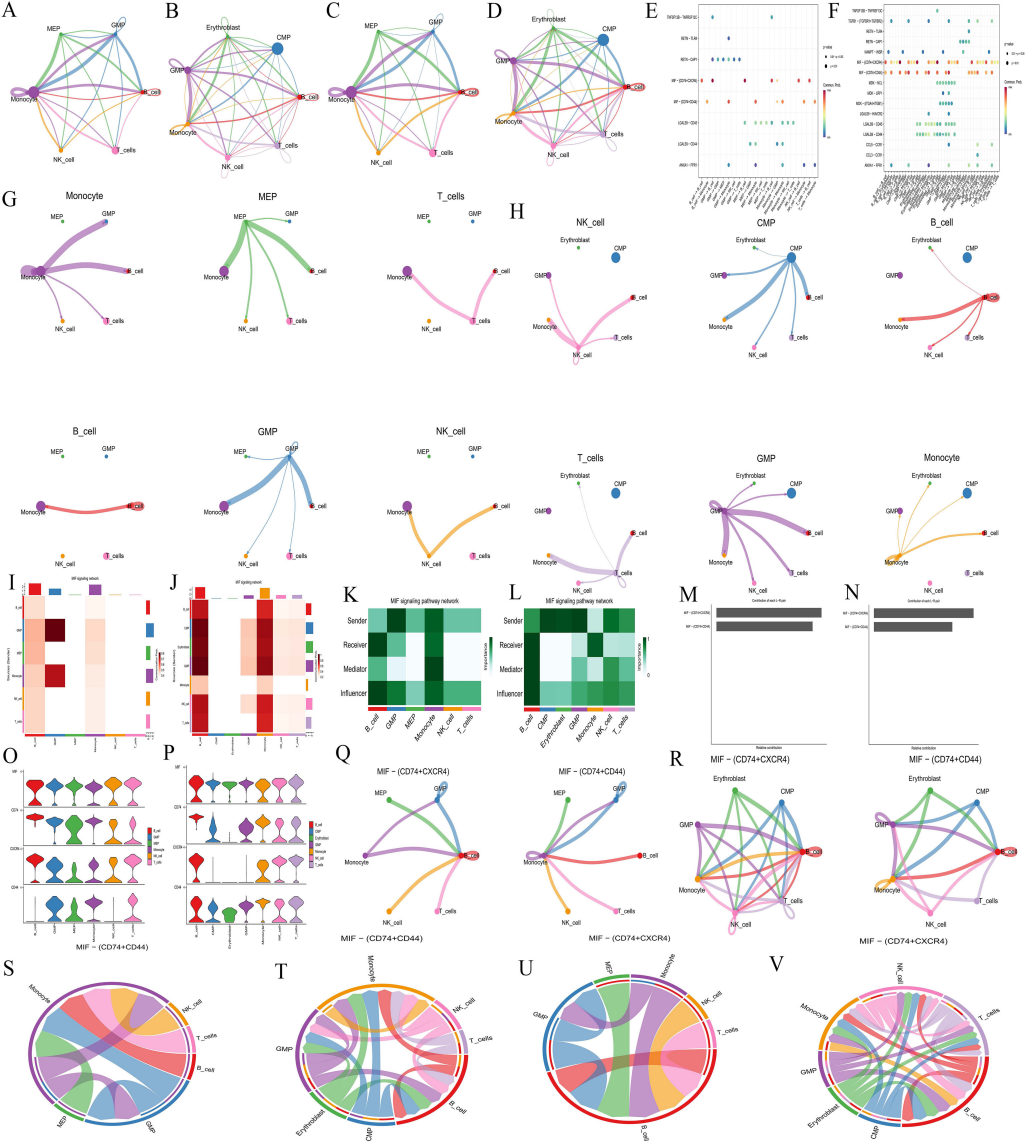
Cell communication analysis. **(A-B, C-D, G-H, Q-R)** Intercellular communication relationship diagrams. Number of interactions: A, normal group; B, AML group. Interaction strength: C, normal group; D, AML group. Communication of each cell with other cells: G, normal group; H, AML group. Cell communication includes MIF − (CD74+CXCR4) and MIF − (CD74+CD44) as receptor-ligand pairs: Q, normal group; R, AML group. Nodes represent cell types, lines represent cell interactions. Line thickness indicates the number of communication types (thicker lines mean more types). Line color represents the ligand cell color. **(E-F)** Bubble plots of receptor-ligand pair. E, normal group; F, AML group. The abscissa represents interacting cells, and the ordinate represents receptor-ligand pairs. **(I-J)** Cell communication heatmaps. I, normal group; J, AML group. Both the abscissa and ordinate represent cell types. The cells in ordinate act as ligand cells sending signals to other cells, while the cells in abscissa act as receptor cells receiving signals. The redder the color, the stronger the interaction between cells. **(K-L)** Types of cell interactions. K, normal group; L, AML group. The abscissa represents cell types, and the ordinate represents interaction types. **(M-N)** Contribution degree of receptor-ligand pairs. M, normal group; N, AML group. The ordinate represents receptor-ligand pair types, and the abscissa represents the contribution degree, with longer length representing higher contribution levels. **(O-P)** Expression level of interacting genes in pathways. O, normal group; P, AML group. The abscissa represents cell types, and the ordinate represents gene names. **(S-V)** Chord diagrams of receptor-ligand pairs interaction. Normal group, S and U; AML group, T and V. Nodes represent cell types, and the lines represent interactions between cells. The thickness of the lines indicates the number of communication types, with thicker lines representing more types. The line color represents the ligand cell color.

Cell communication analysis revealed intercellular communication relationships (**Supplementary Table S3**-CON,S3-AML), interaction counts (CON: **Figure 6A**, AML: **Figure 6B**), interaction strength (CON: **Figure 6C**, AML: **Figure 6D**), and the communication of individual cells with other cell types (CON: **Figure 6G**, AML: **Figure 6H**). Monocyte, MEP, and GMP were ranked highest in the normal group. In AML, CMP, GMP, B-cell, and Monocyte showed the highest rankings. Both the normal and AML groups demonstrated that most cellular communication occurred via two primary receptor-ligand pairs: MIF − (CD74+CXCR4) and MIF − (CD74+CD44) (CON: **Figure 6E**, AML: **Figure 6F**). Visualization of the MIF pathways for both groups indicated that all analyzed cells had the ability to send signals. GMP and Monocyte acted as ligand cells, sending signals to other cells. B-cell, Monocyte, and GMP served as receptor cells, receiving the majority of signals (CON: **Figure 6I**, AML: **Figure 6J**), and also played the role of intermediary and bridge (CON: **Figure 6K**, AML: **Figure 6L**). MIF − (CD74+CXCR4) and MIF − (CD74+CD44) receptor-ligand pairs contributed significantly to the communication network in both groups (CON: **Figure 6M**, AML: **Figure 6N**). The expression levels of genes involved in cell communication were consistent with the observed interactions (CON: **Figure 6O**, AML: **Figure 6P**). Moreover, cell communication utilizing MIF − (CD74+CXCR4) and MIF − (CD74+CD44) as receptor-ligand pairs was clearly depicted (CON: **Figures 6Q, S, U**; AML: **Figures 6R, T, V**).

### 3.2 MR analysis

(1) We conducted a bulk MR analysis on 728 immune cells, identifying 19 positive immune cells for AA (**Supplementary Table S3**-AA positive immune cells), 14 for MDS (**Supplementary Table S3**-MDS positive immune cells), and 27 for AML (**Supplementary Table S3**-AML positive immune cells). Additionally, we analyzed 90 inflammatory factors, identifying 3 positive inflammatory factors for AA (**Supplementary Table S3**-AA positive inflammatory factor), 4 for MDS (**Supplementary Table S3**-MDS positive inflammatory factor), and 2 for AML (**Supplementary Table S3**-AML positive inflammatory factor). (2) Using the identified positive immune cells as exposures and positive inflammatory factors as outcomes, a bulk MR analysis revealed 4 correspondences in AA (**Supplementary Table S3**-AA immune-inflammatory), 2 in MDS (**Supplementary Table S3**-MDS immune-inflammatory), and 2 in AML (**Supplementary Table S3**-AML immune-inflammatory). When inflammatory factors were used as exposures and immune cells as outcomes, 2 correspondences were identified in AA (**Supplementary Table S3**-AA inflammatory-immune), 4 in MDS (**Supplementary Table S3**-MDS inflammatory-immune), and 6 in AML (**Supplementary Table S3**-AML inflammatory-immune). (3) Further analysis using double-positive immune cells as exposures and the three diseases as outcomes revealed 2 correspondences in AA (**Supplementary Table S3**-AA Double positive immune), 4 in MDS (**Supplementary Table S3**-MDS Double positive immune), and 6 in AML (**Supplementary Table S3**-AML Double positive immune). Similarly, using double-positive inflammatory factors as exposures, we found 4 correspondences in AA (**Supplementary Table S3**-AA Double positive inf), 2 in MDS (**Supplementary Table S3**-MDS Double positive inf), and 2 in AML (**Supplementary Table S3**-AML Double positive inf). (4) Ultimately, based on the previous steps, 8 pathways were identified where triple-positive immune cells act through triple-positive inflammatory factors to influence the diseases (**Table 1**). Meanwhile, 12 pathways were identified where triple-positive inflammatory factors influence the diseases via triple-positive immune cells (**Table 2**). The mediating MR analysis confirmed the reliability of results.

**Table 1:** Immune cell→inflammatory factor→disease.

Immune cell	Inflammatory factor	Disease	Figures
FSC-A on Natural Killer T	Leukemia inhibitory factor (LIF)	AA	S6(A)
HLA DR on CD14+ CD16- monocyte	Leukemia inhibitory factor receptor (LIFR)	AA	S6(B)
HLA DR on CD14+ monocyte	Leukemia inhibitory factor receptor (LIFR)	AA	S6(C)
CD39 on CD39+ secreting CD4 regulatory T cell	Tumor necrosis factor ligand superfamily member 12 (TWEAK)	AA	S6(D)
CCR7 on naive CD8+ T cell	Hepatocyte growth factor levels(HGF)	MDS	S6(E)
CD28 on CD28+ CD45RA+ CD8+ T cell	SIR2-like protein 2 (SIRT2)	MDS	S6(F)
CD8 on Terminally Differentiated CD8+ T cell	Natural killer cell receptor 2B4(2B4)	AML	S6(G)
Naive CD4-CD8- T cell Absolute Count	Thymic stromal lymphopoietin(TSLP)	AML	S6(H)

**Table 2:** inflammatory factor→immune cell→disease.

Inflammatory factor	Immune cell	Disease	figures
Leukemia inhibitory factor (LIF)	FSC-A on Natural Killer T	AA	S6(I)
Tumor necrosis factor ligand superfamily member 12 (TWEAK)	CD16 on CD14- CD16+ monocyte	AA	S6(J)
C-C motif chemokine 19 (CCL19)	CD27 on Plasma Blast-Plasma Cell	MDS	S6(K)
SIR2-like protein 2 (SIRT2)	CD25 on CD39+ CD4+ T cell	MDS	S6(L)
C-C motif chemokine 19 (CCL19)	CD19 on B cell	MDS	S6(M)
Interleukin-20 (IL-20)	CD8 on CD28- CD8+ T cell	MDS	S6(N)
Thymic stromal lymphopoietin (TSLP)	CD27 on CD24+ CD27+ B cell	AML	S6(O)
Thymic stromal lymphopoietin (TSLP)	IgD on IgD+ CD38+ B cell	AML	S6(P)
Thymic stromal lymphopoietin (TSLP)	CD33 on CD33dim HLA DR+ CD11b+	AML	S6(Q)
Thymic stromal lymphopoietin (TSLP)	CD33 on CD66b++ myeloid cell	AML	S6(R)
Thymic stromal lymphopoietin (TSLP)	CD25 on CD39+ CD4+ T cell	AML	S6(S)
Thymic stromal lymphopoietin (TSLP)	CD11b on basophil	AML	S6(T)

All heterogeneity tests are detailed in **Supplementary Table S4**-Heterogeneity imm-inf-dise and **Supplementary Table S4**-Heterogeneity inf-imm-dise, while results of pleiotropy tests are presented in **Supplementary Table S4**-Pleiotropy imm-inf-dise and S4-Pleiotropy inf-imm-dise. SNP data for all exposures are available in **Supplementary Table S4**-SNPS of imm-inf-dise and **Supplementary Table S4**-SNPS of inf-imm-dise, as well as **Supplementary Table S4**-SNPS imm-inf-dise (relationship) and **Supplementary Table S4**-SNPS inf-imm-dise (relationship). Results from five MR calculation methods are presented in **Supplementary Table S4**-OR of imm-inf-dise and **Supplementary Table S4**-OR of inf-imm-dise, as well as **Supplementary Table S4**-OR imm-inf-dise(relationship) and **Supplementary Table S4**-OR inf-imm-dise(relationship), and individual SNP analysis results are in Table S-singlesnpOR of imm-inf-dise and **Supplementary Table S4**-singlesnpOR inf-imm-dise, as well as **Supplementary Table S4**-siOR imm-inf-dise(relationship) and **Supplementary Table S4**-siOR inf-imm-dise(relationship). Metrics for each pathway, including beta_all, beta1, beta2, beta_dir, Z-values, and 95% CI, are listed in **Supplementary Table S4**-effect of imm-inf-dise and **Supplementary Table S4**-effect of inf-imm-dise. Forest plots, funnel plots, scatter plots, and leave-one-out forest plots from the four-step screening process of the 20 immunological pathways are shown in **Supplementary Figure S6**, corresponding to **Tables 1**, **2**.

### 3.3 Experimental validation

#### 3.3.1 Expression of common hub genes in normal and AML groups

**Figure 7:**
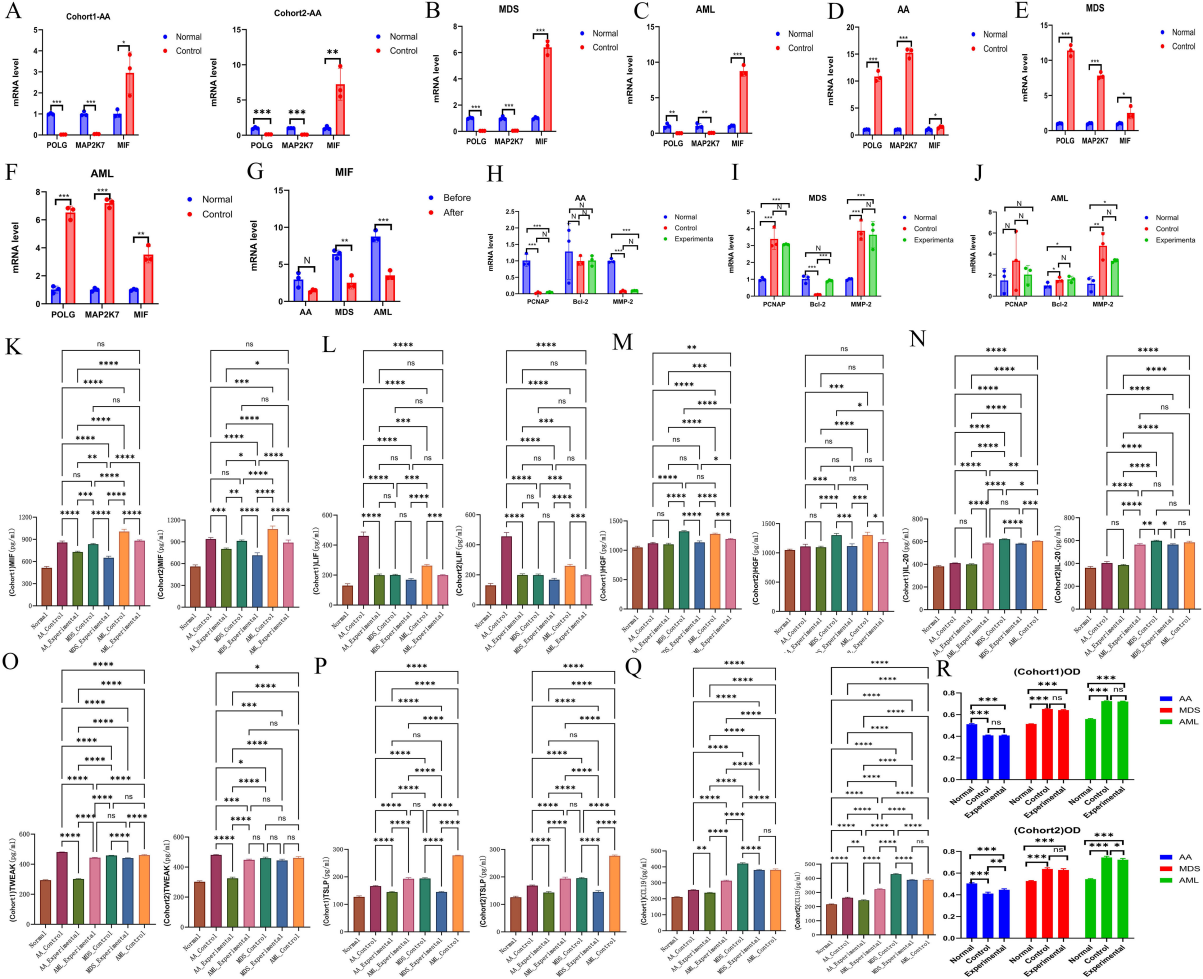
Experimental validation of hub genes. **(A-C)** Validation of hub gene expression. AA, A; MDS, B; AML, C. **(D-F)** Validation of plasmid transfection. AA, D; MDS, E; AML, F. **(G)** Comparison of MIF expression levels before and after plasmid transfection across all three diseases. **(H-J)** Effects of upregulated common hub genes on the expression of PCNA, Bcl-2, and MMP-2. AA, H; MDS, I; AML, J. **(K-Q)** Expression levels of inflammatory factors, including MIF **(K)**, LIF **(L)**, HGF **(M)**, IL-20 **(N)**, TWEAK **(O)**, TSLP **(P)**, and CCL19 **(Q)**. **(R)** Comparison of cell proliferation across different groups for each disease. *P < 0.05; **P < 0.01; ***P < 0.001; ****P < 0.0001.

Both POLG and MAP2K7 exhibited low expression in AA, MDS, and AML, with significant inter-group differences observed among the diseases (**Table 3**, **Supplementary Table S5**-Verification, **Figures 7A-C**), which was consistent with bioinformatic predictions.

**Table 3:** Expression of MIF and hub genes in normal group and AML group.

Diseases		Gene	Normal	Control	t	*P*
AA	Cohort1	POLG	1.001 ± 0.040	0.022 ± 0.002	42.419	0.000
		MAP2K7	1.003 ± 0.103	0.046 ± 0.004	16.020	0.000
		MIF	1.010 ± 0.180	2.952 ± 0.992	-3.337	0.029
	Cohort2	POLG	1.006 ± 0.141	0.119 ± 0.009	10.883	0.000
		MAP2K7	1.000 ± 0.008	0.094 ± 0.015	90.186	0.000
		MIF	1.019 ± 0.251	7.239 ± 2.276	-4.706	0.009
MDS	Cohort1	POLG	1.001 ± 0.048	0.036 ± 0.003	34.669	0.000
		MAP2K7	1.006 ± 0.138	0.047 ± 0.004	12.033	0.000
		MIF	1.002 ± 0.083	6.394 ± 0.538	-17.156	0.000
AML	Cohort1	POLG	1.029 ± 0.305	0.005 ± 0.001	5.815	0.004
		MAP2K7	1.032 ± 0.331	0.042 ± 0.018	5.178	0.007
		MIF	1.004 ± 0.113	8.755 ± 0.755	-17.586	0.000

#### 3.3.2 Plasmid transfection

Plasmid transfection was successfully performed, with cells from each disease group exhibiting high expression of the common hub genes following transfection (**Table 4**, **Supplementary Table S5**-Transfection, **Figures 7D-F**). This indicated successful transfection and that the designed plasmids effectively induced high expression of POLG and MAP2K7 in cells from each disease group, leading to a reduction in MIF expression levels, with a negative correlation between the two (**Table 5**, **Figure 7G**).

**Table 4:** Expression of hub genes after plasmid induction.

Diseases	Gene	Normal	Experimental	t	*P*
AA	POLG	1.001 ± 0.046	10.843 ± 0.964	-17.67	0
	MAP2K7	1.001 ± 0.050	15.239 ± 0.906	-27.173	0
	MIF	1.009 ± 0.170	1.442 ± 0.188	-2.959	0.042
MDS	POLG	1.000 ± 0.052	11.389 ± 0.789	-22.756	0
	MAP2K7	1.003 ± 0.099	7.826 ± 0.457	-25.273	0
	MIF	1.003 ± 0.101	2.521 ± 0.854	-3.057	0.038
AML	POLG	1.022 ± 0.251	6.524 ± 0.462	-18.125	0
	MAP2K7	1.005 ± 0.125	7.198 ± 0.309	-32.181	0
	MIF	1.001 ± 0.055	3.514 ± 0.593	-7.309	0.002

**Table 5:** Comparison of MIF expression before and after plasmid induction.

Disease	Before	After	t	P
AA	2.952 ± 0.992	1.442 ± 0.188	2.59	0.061
MDS	6.394 ± 0.538	2.521 ± 0.854	6.646	0.003
AML	8.755 ± 0.755	3.514 ± 0.593	4	0.001

#### 3.3.3 Impact of overexpressed common hub genes

Compared to normal cells, PCNA was downregulated in AA but upregulated in MDS and AML. Bcl-2 showed no significant change in AA, was downregulated in MDS, and slightly upregulated in AML. MMP-2 was downregulated in AA but upregulated in both MDS and AML. These findings reflected the differences across the disease cells.

Following the induction of common hub genes, only MDS showed significant differences in Bcl-2 expression, while no statistically significant changes were observed in the other disease groups. When considering the overall expression levels of the three genes, the common hub genes had minimal overall impact on the three diseases. They did not affect the proliferation or migration of cells, and the only notable impact was on apoptosis regulation in MDS, with no effect on apoptosis in AML and AA (**Supplementary Table S5**-Changes, **Supplementary Table S5**–LSD-t of Changes **Figures 7H-J**).

#### 3.3.4 ELISA analysis of inflammatory factors expression

Following inducing high expression of common hub genes, levels of characteristic inflammatory factors and MIF showed a trend of reduction across all diseases, suggesting a negative correlation between common hub genes and both MIF and inflammatory factors expression (**Supplementary Table S6**, **Figures 7K-Q**).

#### 3.3.5 MTT assay for cell proliferation

The proliferation rate in AA cells was lower than that of normal cells, while MDS and AML cells exhibited higher proliferation rates. Upon induction of high expression of common hub genes, no significant impact on the proliferation of various cells was observed (**Table 6**, **Figure 7R**, the raw data was provided in **Supplementary Table S7**).

**Table 6:** Cell OD statistics.

Disease	Group	Cohort1					Cohort2				
		Mean 1	Mean 2	S1	S2	*P*	Mean 1	Mean 2	S1	S2	*P*
AA	Normal vs. Control	0.512	0.408	0.006	0.003	0.000	0.505	0.412	0.007	0.013	0.000
	Normal vs. Experimenta	0.512	0.408	0.006	0.004	0.000	0.505	0.446	0.007	0.009	0.000
	Control vs. Experimenta	0.408	0.408	0.003	0.004	0.930	0.412	0.446	0.013	0.009	0.005
MDS	Normal vs. Control	0.514	0.652	0.002	0.007	0.000	0.527	0.639	0.005	0.010	0.000
	Normal vs. Experimenta	0.514	0.642	0.002	0.005	0.000	0.527	0.629	0.005	0.011	0.000
	Control vs. Experimenta	0.652	0.642	0.007	0.005	0.043	0.639	0.629	0.010	0.011	0.265
AML	Normal vs. Control	0.559	0.724	0.005	0.003	0.000	0.544	0.744	0.005	0.011	0.000
	Normal vs. Experimenta	0.559	0.721	0.005	0.004	0.000	0.544	0.724	0.005	0.011	0.000
	Control vs. Experimenta	0.724	0.721	0.003	0.004	0.298	0.744	0.724	0.011	0.011	0.041

## 4 Discussion

AA, MDS, and AML all exhibit pronounced immune dysregulation (1), with intricate interconnections in their transformation and progression (50). Therefore, exploring shared pathogenic genes and associated immune mechanisms is crucial for diagnosis and treatment. In this study, we used bioinformatics to identify the DEGs of each disease. Enrichment analyses revealed that these diseases had strong associations with viral infections, inflammation, endocrine, and metabolism. We had enriched transcription factors from the common upregulated and downregulated gene sets, suggesting that these common transcription factors continuously play an important role in the transcriptional regulation of the three diseases. Ultimately, we identified the common hub genes across three diseases as the downregulated POLG and MAP2K7. GSEA analysis of POLG-related synergistic genes revealed a positive correlation with apoptosis and a negative correlation with heme metabolism and the E2F transcription factor. This suggested anomalies in apoptosis, erythropoiesis, and transcription regulation within these diseases. Using single-cell sequencing and cell communication analysis, we found that the hub genes modulate the immune response primarily via MIF-mediated signaling. Through MR, we screened and confirmed eight immune regulatory pathways, in which immune cells influence these diseases via inflammatory factors, and 12 pathways, in which inflammatory factors act through immune cells, creating a detailed immune regulatory network. Our *in vitro* experiments corroborated the reliability of POLG and MAP2K7 as common hub genes. They exhibited a negative correlation with MIF and inflammatory factors, without regulatory effects on cell proliferation and migration. Therefore, we speculate that they only function in delaying the progression of the disease.

The POLG, mitochondrial DNA polymerase γ gene, is integral for maintaining mitochondrial function (51, 52). The protein encoded by POLG is involved in mitochondrial DNA replication and repair (53). Given that mitochondria are the “powerhouses” of cells, mitochondrial DNA stability is vital for cellular energy production. Studies have reported that instabilities in mitochondrial-related genes can lead to erythroid dysplasia (54), megaloblastic anemia, and increased risk of impaired lymphopoiesis (55), features that align closely with the characteristics of AA, MDS, and AML. Furthermore, reduced POLG function has been found to be linked to heightened inflammatory responses following viral infections (56), providing direct evidence of its immunoregulatory capability and aligning with our experimental results. The MAP2K7, mitogen-activated protein kinase kinase-7 gene, encodes the protein that is involved in the MAPK signaling pathway, which plays a crucial role in cell growth, differentiation, apoptosis, and responses to external stimuli (57). Studies have demonstrated that MAP2K7 can regulate tumor cell proliferation, invasion, and metastasis (58, 59), with activation of related pathways exhibiting anti-leukemia effects (60). Research on MAP2K7 in the context of AA and MDS is sparse, rendering our investigation particularly meaningful. Additionally, some studies have shown that KLF4 inhibits MAP2K7, and its deletion can activate MAP2K7 in T-cell acute lymphoblastic leukemia (ALL) (61, 62). In our study, KLF4 did not show significant differences in AA but was upregulated in MDS and AML, consistent with the known negative correlation between KLF4 and MAP2K7 (61, 62). MAP2K7 has also been reported to enhance antiviral capabilities and plays a crucial role in immune regulation (63, 64).

The enrichment analysis underscored the critical roles of viral infection, inflammation, endocrine, and metabolism in disease progression. Both viral and inflammatory factors ultimately drive abnormal immune activation, aligning with previous studies (6, 20, 25, 28). While endocrine and metabolism are thought to be associated with AML (65), their connection to AA and MDS remains largely unexplored. Immunocyte infiltration analysis revealed that AA, MDS, and AML exhibit distinct patterns of immune cell infiltration compared to normal cells. As disease progresses, the association between the common hub genes and immune cells intensifies, suggesting a positive correlation, and underscoring their critical roles in immune regulation.

In the normal group, single-cell clustering occurred in six cell types, including Monocyte, T-cell, NK-cell, B-cell, GMP, and MEP. In AML, clustering was observed in seven cell types, including CMP, GMP, T-cell, NK-cell, B-cell, Monocyte, and Erythroblast. This is consistent with prior studies (66), which identified 11 main cell types in AML, including T-cell, NK-cell, CMP, Myeloid, GMP, MEP, Promono, HSC, B-cell, and erythroid cell, confirming the importance of immune cells. Furthermore, a single-cell study on AA identified the following main cell types: a mixed population of hematopoietic stem cell and multipotent progenitor cell (HSC/MPP), lymphoid primed multipotent progenitor cell (LMPP), megakaryocyte‐erythroid progenitor (MEP), multipotent lymphoid progenitor (MLP), and eosinophil-basophil-mast cell progenitor (EBM) (36), supporting the significance of immune cells as well. Similarly, a single-cell study on MDS revealed that NK-cell, T-cell, B-cells, GMP, megakaryocyte-erythrocyte progenitor (MEP), erythroid progenitor (Eryp), and plasmacytoid dendritic cell (pDC) were the predominant cell types (35), affirming the essential role of immune cells. Integrating our findings with previous studies, we established a comprehensive single-cell analysis across the three diseases, noting both similarities and differences in immune cell compositions, which was highly similar to our immune infiltration results.

However, due to the lack of single - cell samples for AA and MDS, we were unable to complete this part of the analysis. Although we incorporated others’ single - cell analyses of AA and MDS into our research to construct a complete system, this undoubtedly makes the overall results subject to the quality of others’ research. As a result, the robustness of the single - cell analysis in this paper is compromised, which is a shortcoming of this study.

Cell communication analysis identified Monocyte, MEP, and GMP as the primary signaling cells in healthy controls, while in AML, CMP, GMP, B-cell, and Monocyte dominated. This highlights the shared involvement of immune cells in cellular communication, with MIF−(CD74+CXCR4) and MIF−(CD74+CD44) emerging as major receptor-ligand pairs. MIF has been confirmed as an essential factor in the pathogenesis of AML (67), and it has been shown to have close contact with MDS (68). However, there is limited research on the relationship between MIF and AA. Considering the shared hub genes and their relationships, we hypothesized that MIF and AA are closely related. MIF, a crucial cytokine, is widely expressed across various cell types, including immune cells like macrophages, T-cells, and B-cells, as well as non-immune cells such as epithelial and endothelial cells (69). MIF participates in the activation of macrophages and other immune cells, and initiates and amplifies the inflammatory response by inhibiting macrophage migration (70). It also promotes tumor cell growth, angiogenesis, and metastasis (71, 72). Our results confirmed that the common hub genes trigger subsequent immune regulatory networks through MIF-mediated signaling, exerting different influences on the three diseases.

Based on the previous functional analysis of POLG and MAP2K7 as well as the regulatory analysis of MIF, it can be inferred that the abnormally low expression of POLG first leads to impaired mitochondrial function (51, 52). This, in turn, affects many cellular functions, including an enhanced inflammatory response of cells after viral infection (55). The abnormally low expression of MAP2K7 impacts the body’s immune function and weakens the body’s antiviral ability (63, 64). The abnormally low expression of these two genes ultimately affects the normal function of MIF, causing a disorder in its functions of inhibiting macrophage migration and initiating and amplifying the inflammatory response (70). This may contribute to the progression of the disease.

The above analyses underscored the critical role of the immune in disease transformation and suggested that MIF could trigger complex immune regulatory networks. The networks, comprising inflammatory factors and immune cells, are interdependent, adding complexity to immune regulation (73, 74). To refine our findings, we collected 728 immune cell types and 90 inflammatory factors, conducting bulk MR analyses to map their interactions. Ultimately, we identified 20 distinct pathways, establishing a comprehensive immune regulatory network for the three diseases. By analyzing inflammatory factors, we linked the common hub genes and MIF with these 20 pathways, observing that the types and densities of immune cells associated with the hub genes varied across disease stages (AA: **Figure 4M**, MDS: **Figure 4N**, AML: **Figure 4O**). The regulatory strength was positively correlated with disease progression, as MIF was modulated differently by the hub genes at various disease stages, and it drove divergent immune networks. These regulatory pathways likely extend beyond the 20 we identified, and further *in vitro* experiments are necessary to elucidate their precise impacts on cellular function across different disease contexts.

Through our experiments, we first confirmed that POLG and MAP2K7 were expressed at lower levels in cells from all three diseases compared to normal cells. RT-qPCR results indicated that these hub genes did not influence cell proliferation or migration in AA, MDS, or AML. Consistent with the MTT assay results, POLG and MAP2K7 only affected anti-apoptotic properties in MDS cells, with no impact on apoptosis in AML or AA cells. We speculate that the reason for the decreased influence of hub genes on cell functions might be the differences between the model-building environment and the *in-vivo* environment. For instance, the model-building environment lacks the immune organs present in the body, which significantly affects the quantity and renewability of immune cells. Additionally, as the immune cells in the model-building environment become exhausted, their immune regulatory ability continuously declines until it is completely depleted. Eventually, the off-target effects of regulation gradually emerge. Furthermore, the expression of these common hub genes was inversely correlated with MIF expression. ELISA analysis demonstrated a negative correlation between these hub genes and MIF as well as with several inflammatory factors. Prior studies have shown a positive association between MIF and LIF (75, 76), TNF (77, 78), HGF (79, 80), IL-20 (81), and TSLP (82, 83), trends consistent with our observations.

Despite these findings, limitations remain. Direct validation of immune cells involved in the identified 20 immune regulatory pathways remains incomplete. Meanwhile, in the bioinformatics section, due to the small number of samples in the AA analysis, there is a significant imbalance in the quantity between MDS and AML samples. This may lead to a certain degree of bias in the experimental results, which might amplify the impact effect of hub genes in AA. Although we have compensated for this deficiency to some extent during the experimental stage, this still remains an issue that requires attention.

Since POLG and MAP2K7 did not directly modulate genes such as PCNA, Bcl-2, or MMP-2 to exert cellular regulatory effects, they could not reverse or induce functional changes in already altered cells. Our study suggested that POLG and MAP2K7 may mitigate the immune-driven progression of these diseases to some extent. Their normal expression appeared to play a crucial role in maintaining cells in a normal state and preventing pathological transformation. However, these genes cannot affect the function of cells that have already undergone malignant transformation, as they lack the ability to reverse cell state and induce apoptosis or functional alterations at various disease stages.

Our research also has important value for clinical diagnosis and treatment. Since hub genes play a crucial role in maintaining the normal functions of cells, for patients with low expression of these two hub genes discovered clinically, it is suggested that attention should be paid to screening for these three diseases. And it is expected that more detailed studies will be carried out in the future to determine whether there is a risk of the disease continuing to evolve. At the same time, through more clinical cohort studies in the future, it is highly likely that clinical physicians will be able to delay or inhibit the progression of diseases by means of inducing high expression of hub genes. This undoubtedly brings new hope to patients in the early stages of the disease.

## 5 Conclusion

**Figure 8:**
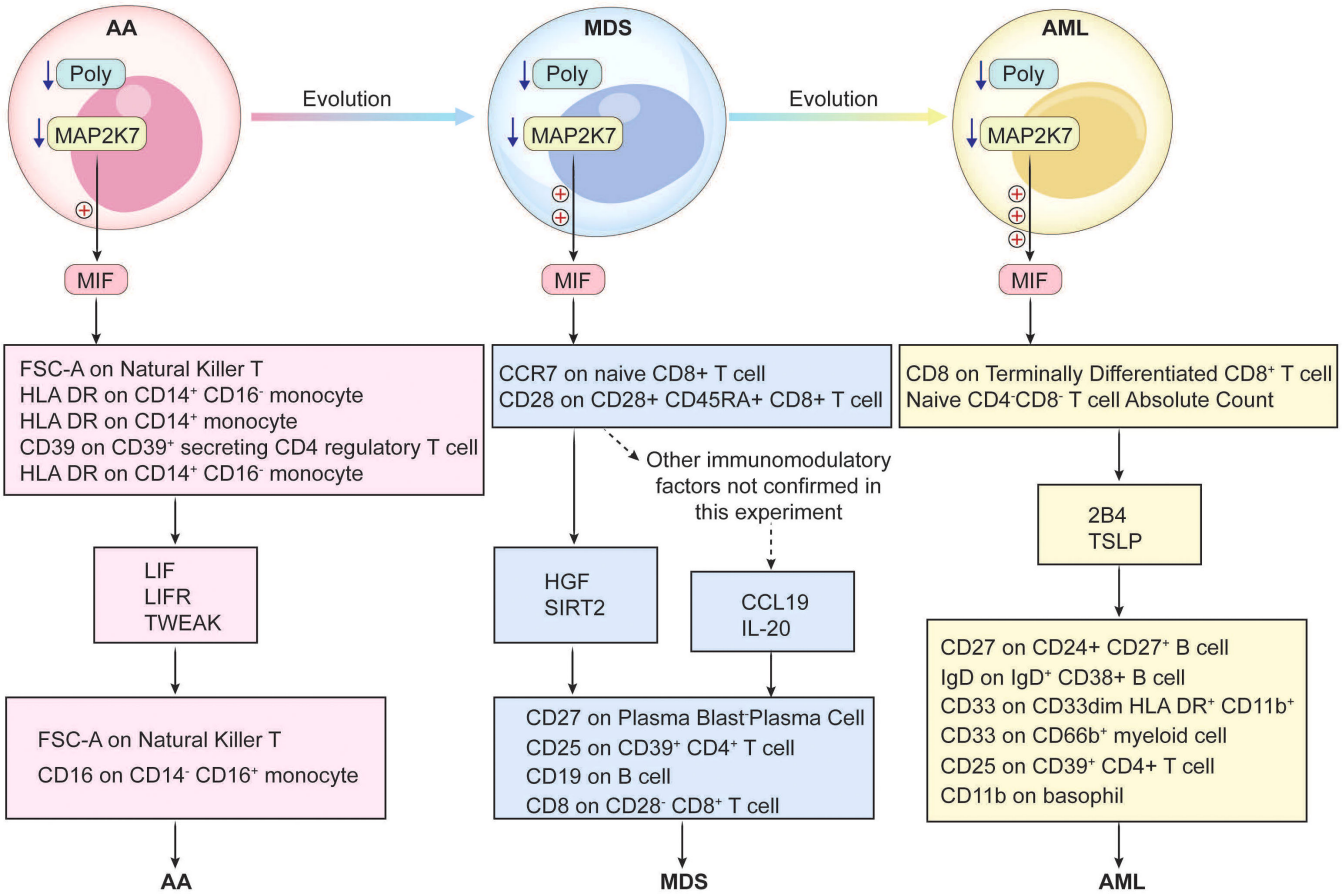
Schematic Diagrams of the Regulatory Mechanisms of AA, MDS, and AML.

POLG and MAP2K7 play a significant role throughout the progression from AA to MDS and ultimately to AML. By modulating immune regulatory pathways through MIF-mediated signaling, these genes subsequently modulate multiple downstream immune regulatory mechanisms, thereby influencing disease transformation and progression (**Figure 8**).

## Data Availability

The original contributions presented in the study are included in the article/**Supplementary Material**. Further inquiries can be directed to the corresponding author.
