# Supplementary material for: Identification of common hub genes and construction of immune regulatory networks in aplastic anemia, myelodysplastic syndromes, and acute myeloid leukemia
Source: Front Immunol. 2025 May 8;16:1547289. doi: 10.3389/fimmu.2025.1547289 (PMC12095185; doi:10.3389/fimmu.2025.1547289)
Supplement: Supplementary file 8 [file Table8.docx]

**STROBE-MR checklist of recommended items to address in reports of Mendelian randomization studies**^1^ ^2^

| **Item No.** | **Section** | **Checklist item** | **Page No.** | **Relevant text from manuscript** |
| --- | --- | --- | --- | --- |
| 1 | **TITLE and ABSTRACT** | Indicate Mendelian randomization (MR) as the study’s design in the title and/or the abstract if that is a main purpose of the study | 1 | We performed Mendelian randomization to screen inflammatory factors and immune cells. |
|  | **INTRODUCTION** |  |  |  |
| 2 | **Background** | Explain the scientific background and rationale for the reported study. What is the exposure? Is a potential causal relationship between exposure and outcome plausible? Justify why MR is a helpful method to address the study question | N | Not performed |
| 3 | **Objectives** | State specific objectives clearly, including pre-specified causal hypotheses (if any). State that MR is a method that, under specific assumptions, intends to estimate causal effects | 2 | We identified key mediators through cell communication analysis and constructed immune regulatory networks using Mendelian Randomization (MR) analysis |
|  | **METHODS** |  |  |  |
| 4 | **Study design and data sources** | Present key elements of the study design early in the article. Consider including a table listing sources of data for all phases of the study. For each data source contributing to the analysis, describe the following: |  |  |
|  | a) | Setting: Describe the study design and the underlying population, if possible. Describe the setting, locations, and relevant dates, including periods of recruitment, exposure, follow-up, and data collection, when available. | N | Not performed |
|  | b) | Participants: Give the eligibility criteria, and the sources and methods of selection of participants. Report the sample size, and whether any power or sample size calculations were carried out prior to the main analysis | 4 | Data on AA were sourced from the IEU database (https://gwas.mrcieu.ac.uk/) (GWAS ID: ebi-a-GCST90018794), comprising 473,500 samples and 24,192,378 SNPs. Data for AML, MDS, immune cells, and inflammatory factors were retrieved from the EBI GWAS Catalog (https://www.ebi.ac.uk/gwas/). Accession numbers of AML and MDS were GCST90435652 and GCST9004394, respectively. Immune cell accession numbers ranged from GCST90274758 to GCST90274848, covering 728 immune cell types and their corresponding GWAS IDs (Table S2). Inflammatory factors had accession numbers from GCST90274758 to GCST9027484, encompassing 90 immune cell types with IDs listed in the EBI GWAS Catalog (Table S3). All samples were drawn from European populations. |
|  | c) | Describe measurement, quality control and selection of genetic variants | 4 | IV needs to satisfy three assumptions: relevance, independence, and exclusion restriction. All IVs must undergo linkage disequilibrium (LD) test, heterogeneity test, and pleiotropy test. |
|  | d) | For each exposure, outcome, and other relevant variables, describe methods of assessment and diagnostic criteria for diseases | N | Not performed |
|  | e) | Provide details of ethics committee approval and participant informed consent, if relevant | 4 | and the original studies had been ethically approved. |
| 5 | **Assumptions** | Explicitly state the three core IV assumptions for the main analysis (relevance, independence and exclusion restriction) as well assumptions for any additional or sensitivity analysis | 4 | IV needs to satisfy three assumptions: relevance, independence, and exclusion restriction. All IVs must undergo linkage disequilibrium (LD) test, heterogeneity test, and pleiotropy test. |
| 6 | **Statistical methods: main analysis** | Describe statistical methods and statistics used |  |  |
|  | a) | Describe how quantitative variables were handled in the analyses (i.e., scale, units, model) | N | Not performed |
|  | b) | Describe how genetic variants were handled in the analyses and, if applicable, how their weights were selected | 4-5 | (1) IVs should adhere to genome-wide significance thresholds (P < 5.0 × 10^-8). If significant SNPs were unavailable, SNPs with P < 5×10^-6 were considered candidates; (2) LD assessed using European samples from the 1000 Genomes Projects was treated as the reference. The SNPs with the lowest P-values at R2 = 0.001 (clumping window size = 10,000 kb) were considered; (3) SNPs with a minor allele frequency (MAF) ≤ 0.01 were excluded; (4) Palindromic SNPs (A/T with ambiguous allele frequencies or G/C polymorphisms) were excluded when harmonizing exposure and outcome data. For each SNP included in the analysis, the following methods were used to calculate R2 and F values for efficiency evaluation according to the data situation: R2 = 2*EAF*(1-EAF)*β2 or R2 = β2/(β2+SE2*N), and F = R2(N-2)/(1-R2), ensuring F ≥ 10. |
|  | c) | Describe the MR estimator (e.g. two-stage least squares, Wald ratio) and related statistics. Detail the included covariates and, in case of two-sample MR, whether the same covariate set was used for adjustment in the two samples | N | Not performed |
|  | d) | Explain how missing data were addressed | 4 | (1) IVs should adhere to genome-wide significance thresholds (P < 5.0 × 10^-8). If significant SNPs were unavailable, SNPs with P < 5×10^-6 were considered candidates; |
|  | e) | If applicable, indicate how multiple testing was addressed | N | Not performed |
| 7 | **Assessment of assumptions** | Describe any methods or prior knowledge used to assess the assumptions or justify their validity | 9 | indicating that all three diseases had immune differences compared to normal cells. |
| 8 | **Sensitivity analyses and additional analyses** | Describe any sensitivity analyses or additional analyses performed (e.g. comparison of effect estimates from different approaches, independent replication, bias analytic techniques, validation of instruments, simulations) | 5 | Cochran's Q statistic was utilized to measure the heterogeneity of IVs, with P > 0.05 indicating no heterogeneity, calculated using the mr_heterogeneity function. A random effects model was applied when significant heterogeneity was detected among SNPs, otherwise, a fixed effects model would be used. Additionally, a "leave-one-out" analysis was performed to identify potential outlier SNPs. Pleiotropy was evaluated based on the intercept calculated by MR-Egger regression using mr_pleiotropy_test. |
| 9 | **Software and pre-registration** |  |  |  |
|  | a) | Name statistical software and package(s), including version and settings used | 4 | utilizing the package "TwosampleMR".  Statistical analyses were performed using SPSS 25.0 and R 4.4.1. |
|  | b) | State whether the study protocol and details were pre-registered (as well as when and where) | N | Not performed |
|  | **RESULTS** |  |  |  |
| 10 | **Descriptive data** |  |  |  |
|  | a) | Report the numbers of individuals at each stage of included studies and reasons for exclusion. Consider use of a flow diagram | N | Not performed |
|  | b) | Report summary statistics for phenotypic exposure(s), outcome(s), and other relevant variables (e.g. means, SDs, proportions) | N | Not performed |
|  | c) | If the data sources include meta-analyses of previous studies, provide the assessments of heterogeneity across these studies | N | Not performed |
|  | d) | For two-sample MR:  i.  Provide justification of the similarity of the genetic variant-exposure associations between the exposure and outcome samples  ii.  Provide information on the number of individuals who overlap between the exposure and outcome studies | N | Not performed |
| 11 | **Main results** |  |  |  |
|  | a) | Report the associations between genetic variant and exposure, and between genetic variant and outcome, preferably on an interpretable scale | 10-11 | Ultimately, based on the previous steps, 8 pathways were identified where triple-positive immune cells act through triple-positive inflammatory factors to influence the diseases (Table 1). Meanwhile, 12 pathways were identified where triple-positive inflammatory factors influence the diseases via triple-positive immune cells (Table 2). |
|  | b) | Report MR estimates of the relationship between exposure and outcome, and the measures of uncertainty from the MR analysis, on an interpretable scale, such as odds ratio or relative risk per SD difference | 11 | The mediating MR analysis confirmed the reliability of results.  All heterogeneity tests are detailed in Table S16, while results of pleiotropy tests are presented in Table S17. SNP data for all exposures are available in Table S18. Results from five MR calculation methods are presented in Table S19, and individual SNP analysis results are in Table S20. Metrics for each pathway, including beta_all, beta1, beta2, beta_dir, Z-values, and 95% CI, are listed in Table S21. |
|  | c) | If relevant, consider translating estimates of relative risk into absolute risk for a meaningful time period | N | Not performed |
|  | d) | Consider plots to visualize results (e.g. forest plot, scatterplot of associations between genetic variants and outcome versus between genetic variants and exposure) | 11 | Forest plots, funnel plots, scatter plots, and leave-one-out forest plots from the four-step screening process of the 20 immunological pathways are shown in Figure S6, corresponding to Tables 1 and 2. |
| 12 | **Assessment of assumptions** |  |  |  |
|  | a) | Report the assessment of the validity of the assumptions | N | Not performed |
|  | b) | Report any additional statistics (e.g., assessments of heterogeneity across genetic variants, such as *I^2^*, Q statistic or E-value) | 11 | All heterogeneity tests are detailed in Table S16, while results of pleiotropy tests are presented in Table S17. |
| 13 | **Sensitivity analyses and additional analyses** |  |  |  |
|  | a) | Report any sensitivity analyses to assess the robustness of the main results to violations of the assumptions | 11 | All heterogeneity tests are detailed in Table S16, while results of pleiotropy tests are presented in Table S17. |
|  | b) | Report results from other sensitivity analyses or additional analyses | N | Not performed |
|  | c) | Report any assessment of direction of causal relationship (e.g., bidirectional MR) | 10 | Further analysis using double-positive immune cells as exposures and the three diseases as outcomes revealed 2 correspondences in AA (Table S14-AA), 4 in MDS (Table S14-MDS), and 6 in AML (Table S14-AML). Similarly, using double-positive inflammatory factors as exposures, we found 4 correspondences in AA (Table S15-AA), 2 in MDS (Table S15-MDS), and 2 in AML (Table S15-AML). |
|  | d) | When relevant, report and compare with estimates from non-MR analyses | N | Not performed |
|  | e) | Consider additional plots to visualize results (e.g., leave-one-out analyses) | 11 | Forest plots, funnel plots, scatter plots, and leave-one-out forest plots from the four-step screening process of the 20 immunological pathways are shown in Figure S6, corresponding to Tables 1 and 2. |
|  | **DISCUSSION** |  |  |  |
| 14 | **Key results** | Summarize key results with reference to study objectives | 12 | Through MR, we screened and confirmed eight immune regulatory pathways, in which immune cells influence these diseases via inflammatory factors, and 12 pathways, in which inflammatory factors act through immune cells, creating a detailed immune regulatory network. |
| 15 | **Limitations** | Discuss limitations of the study, taking into account the validity of the IV assumptions, other sources of potential bias, and imprecision. Discuss both direction and magnitude of any potential bias and any efforts to address them | 14 | Despite these findings, limitations remain. Direct validation of immune cells involved in the identified 20 immune regulatory pathways remains incomplete. |
| 16 | **Interpretation** |  |  |  |
|  | a) | Meaning: Give a cautious overall interpretation of results in the context of their limitations and in comparison with other studies | 13 | To refine our findings, we collected 728 immune cell types and 90 inflammatory factors, conducting bulk MR analyses to map their interactions. Ultimately, we identified 20 distinct pathways, establishing a comprehensive immune regulatory network for the three diseases. By analyzing inflammatory factors, we linked the common hub genes and MIF with these 20 pathways, observing that the types and densities of immune cells associated with the hub genes varied across disease stages |
|  | b) | Mechanism: Discuss underlying biological mechanisms that could drive a potential causal relationship between the investigated exposure and the outcome, and whether the gene-environment equivalence assumption is reasonable. Use causal language carefully, clarifying that IV estimates may provide causal effects only under certain assumptions | 14 | The regulatory strength was positively correlated with disease progression, as MIF was modulated differently by the hub genes at various disease stages, and it drove divergent immune networks. These regulatory pathways likely extend beyond the 20 we identified, |
|  | c) | Clinical relevance: Discuss whether the results have clinical or public policy relevance, and to what extent they inform effect sizes of possible interventions | 14 | Our study suggested that POLG and MAP2K7 may mitigate the immune-driven progression of these diseases to some extent, suggesting a protective function. Their normal expression appeared to play a crucial role in maintaining cells in a normal state and preventing pathological transformation. However, these genes cannot affect the function of cells that have already undergone malignant transformation, as they lack the ability to reverse cell state and induce apoptosis or functional alterations at various disease stages. |
| 17 | **Generalizability** | Discuss the generalizability of the study results (a) to other populations, (b) across other exposure periods/timings, and (c) across other levels of exposure | N | Not performed |
|  | **OTHER INFORMATION** |  |  |  |
| 18 | **Funding** | Describe sources of funding and the role of funders in the present study and, if applicable, sources of funding for the databases and original study or studies on which the present study is based | 15 | This work was supported by the Shandong Maternal and Child Health Association for their support through grant No. SFYXH-2023W043.  We thank Weifang City young medical talent lifting project support. |
| 19 | **Data and data sharing** | Provide the data used to perform all analyses or report where and how the data can be accessed, and reference these sources in the article. Provide the statistical code needed to reproduce the results in the article, or report whether the code is publicly accessible and if so, where | 15 | The dataset(s) supporting the conclusions of this article is(are) included within the article (and its additional file(s)). |
| 20 | **Conflicts of Interest** | All authors should declare all potential conflicts of interest | 40 | Not performed |

This checklist is copyrighted by the Equator Network under the Creative Commons Attribution 3.0 Unported (CC BY 3.0) license.

1. Skrivankova VW, Richmond RC, Woolf BAR, Yarmolinsky J, Davies NM, Swanson SA, et al. Strengthening the Reporting of Observational Studies in Epidemiology using Mendelian Randomization (STROBE-MR) Statement. JAMA. 2021;under review.

2. Skrivankova VW, Richmond RC, Woolf BAR, Davies NM, Swanson SA, VanderWeele TJ, et al. Strengthening the Reporting of Observational Studies in Epidemiology using Mendelian Randomisation (STROBE-MR): Explanation and Elaboration. BMJ. 2021;375:n2233.
