# Supplementary figures and images for: Identification of common hub genes and construction of immune regulatory networks in aplastic anemia, myelodysplastic syndromes, and acute myeloid leukemia
Source: Front Immunol. 2025 May 8;16:1547289. doi: 10.3389/fimmu.2025.1547289 (PMC12095185; doi:10.3389/fimmu.2025.1547289)

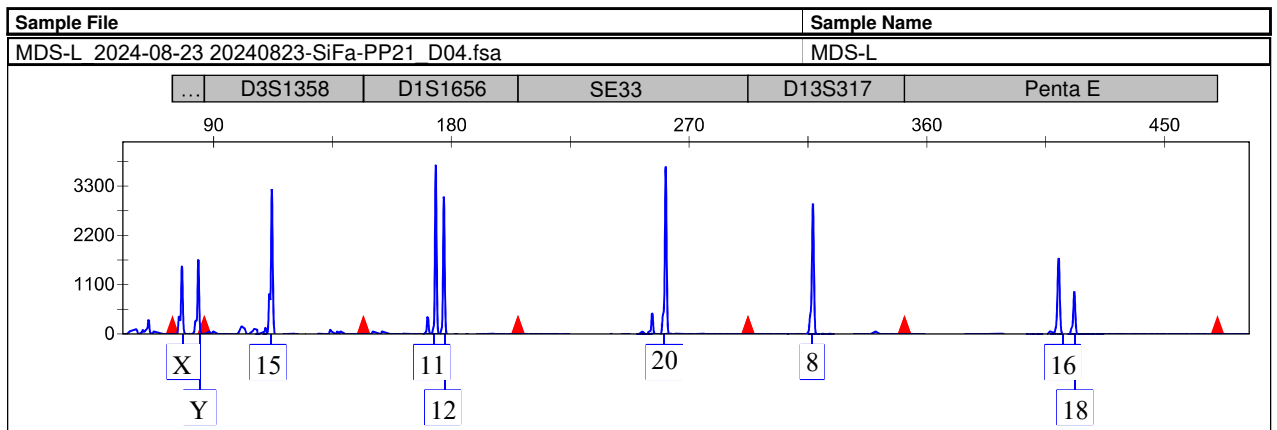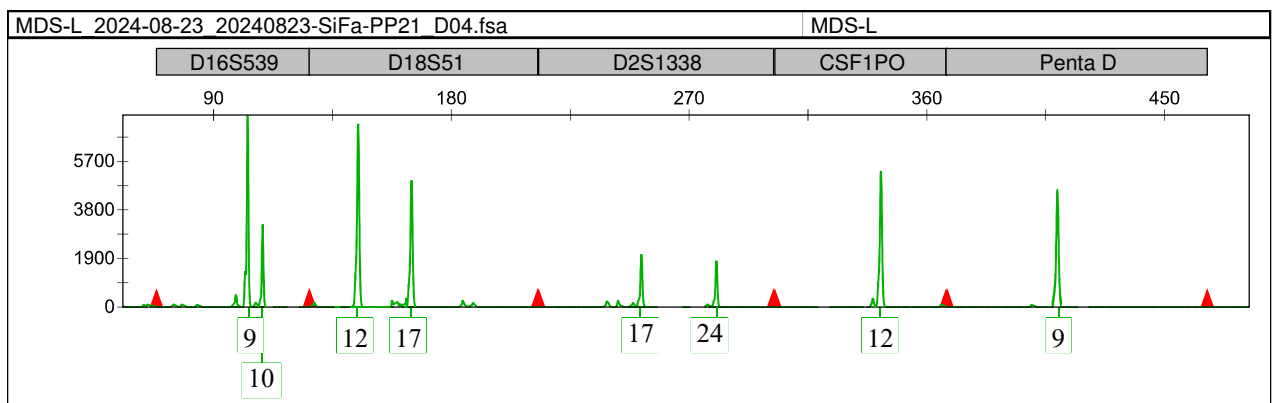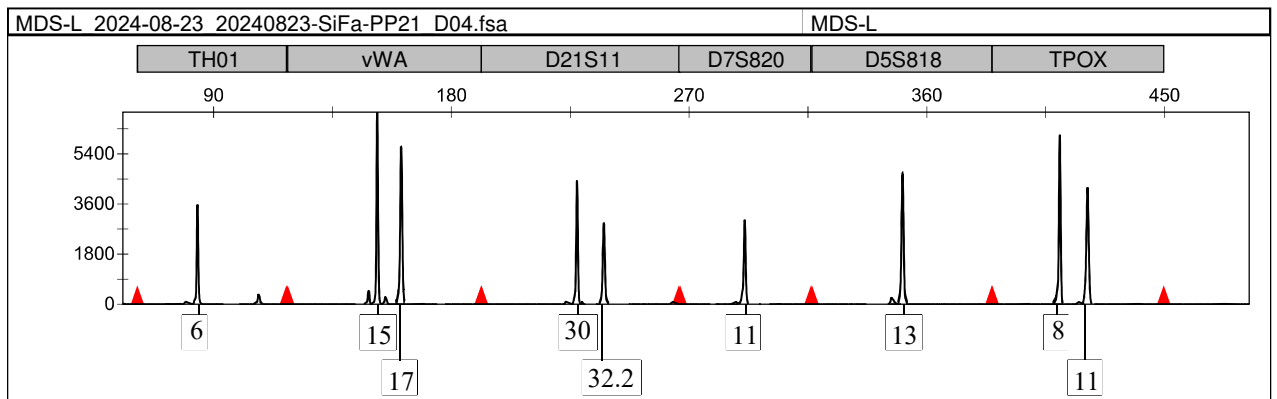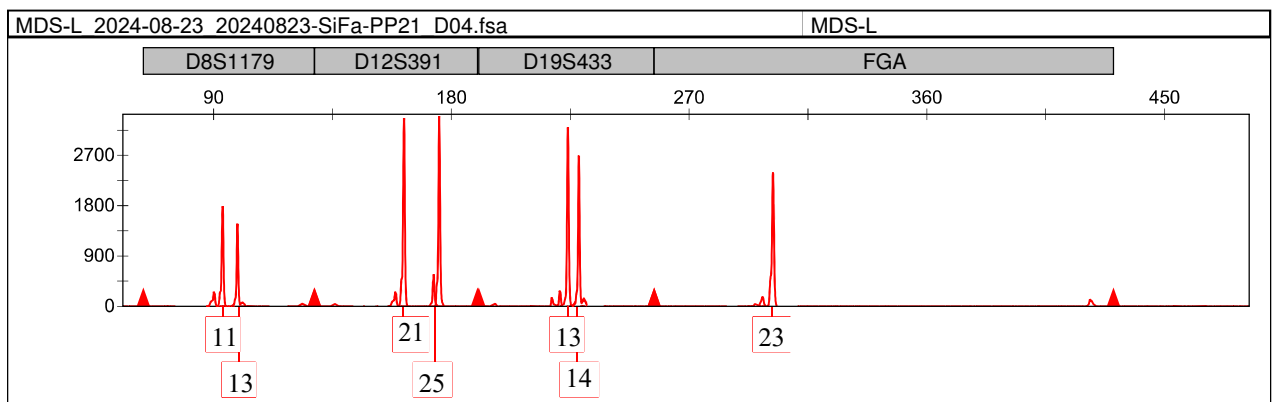

Supplement: Supplementary file 9 [file DataSheet1.pdf]

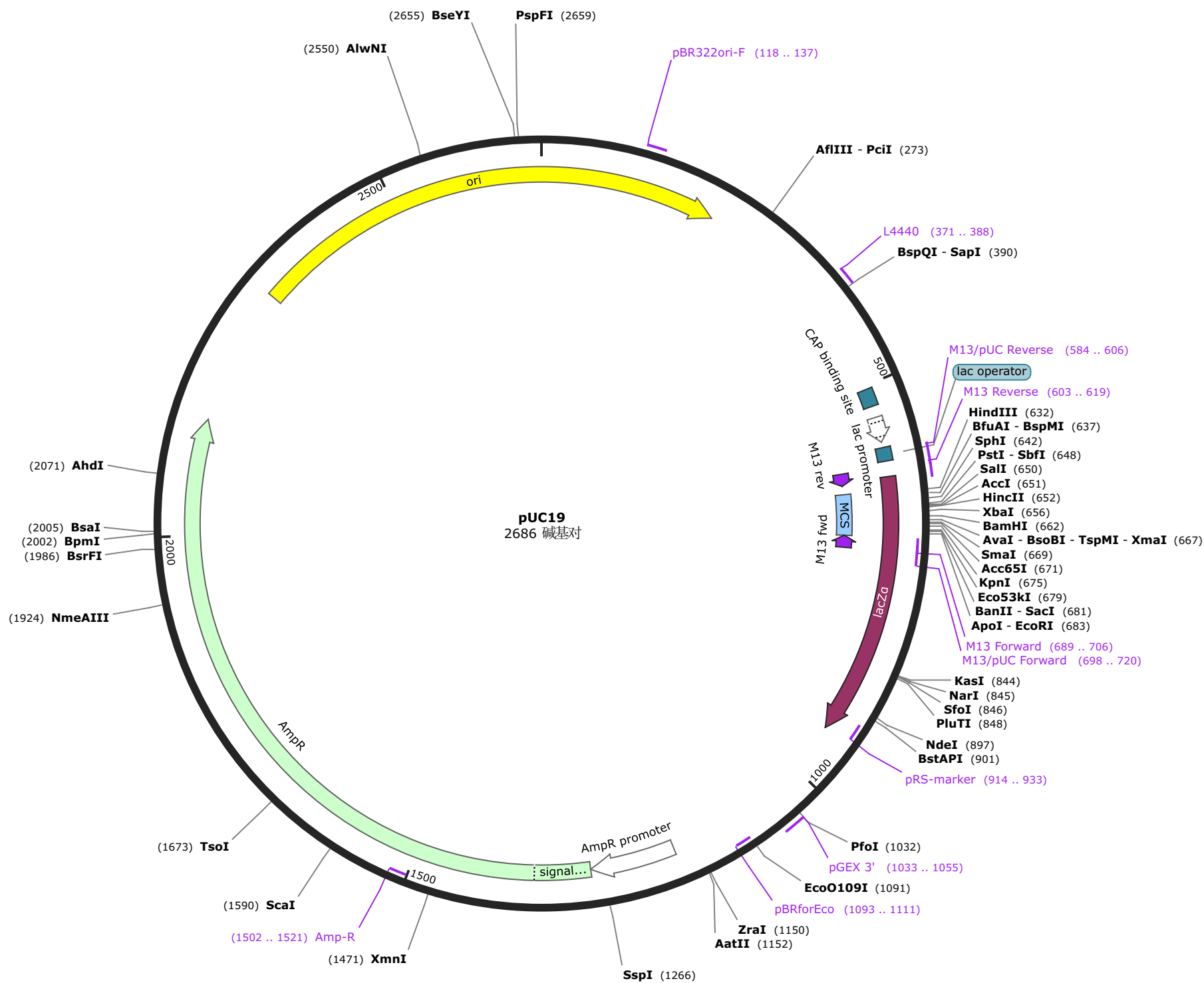

Supplement: Supplementary file 11 [file DataSheet3.pdf]

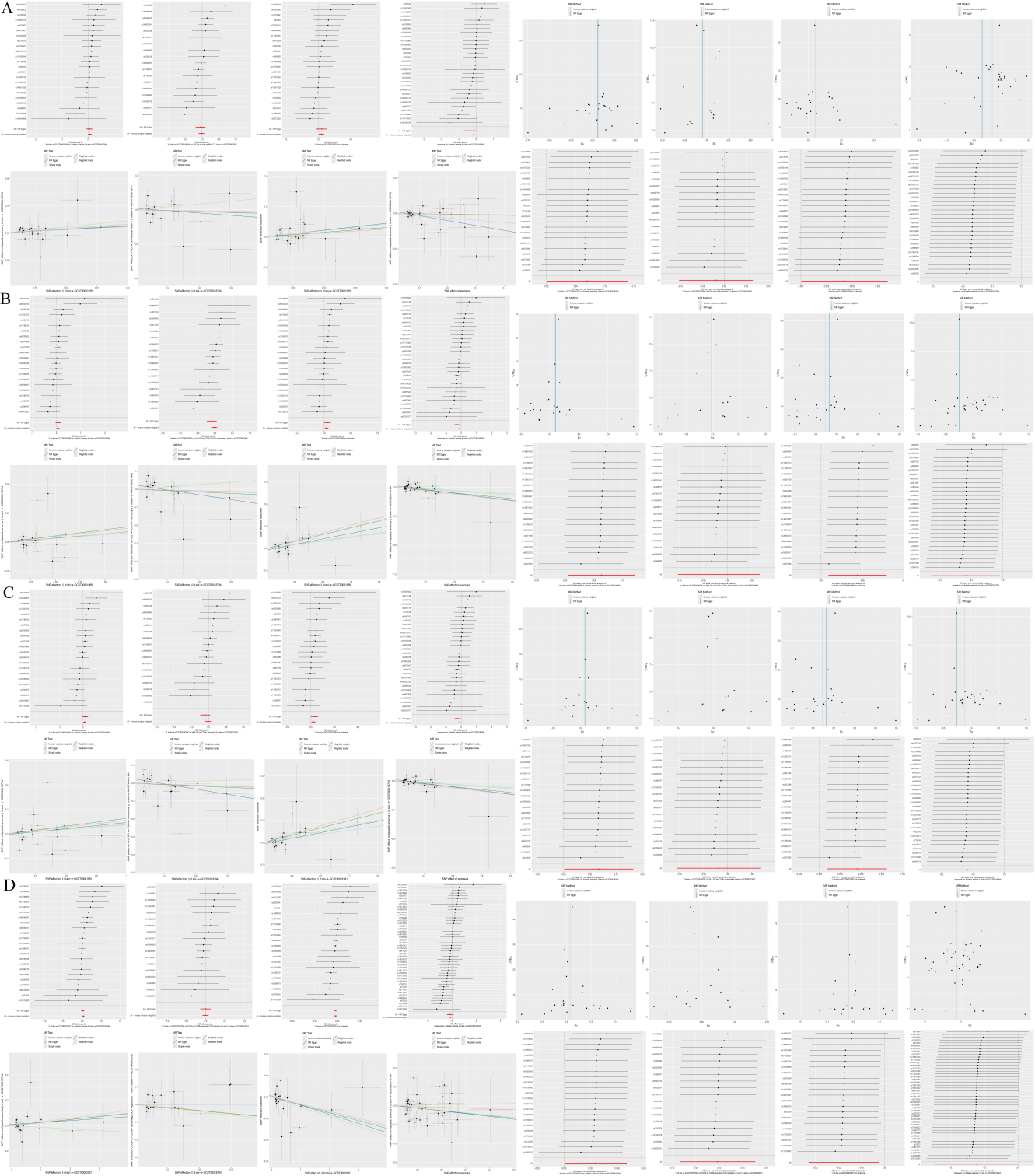

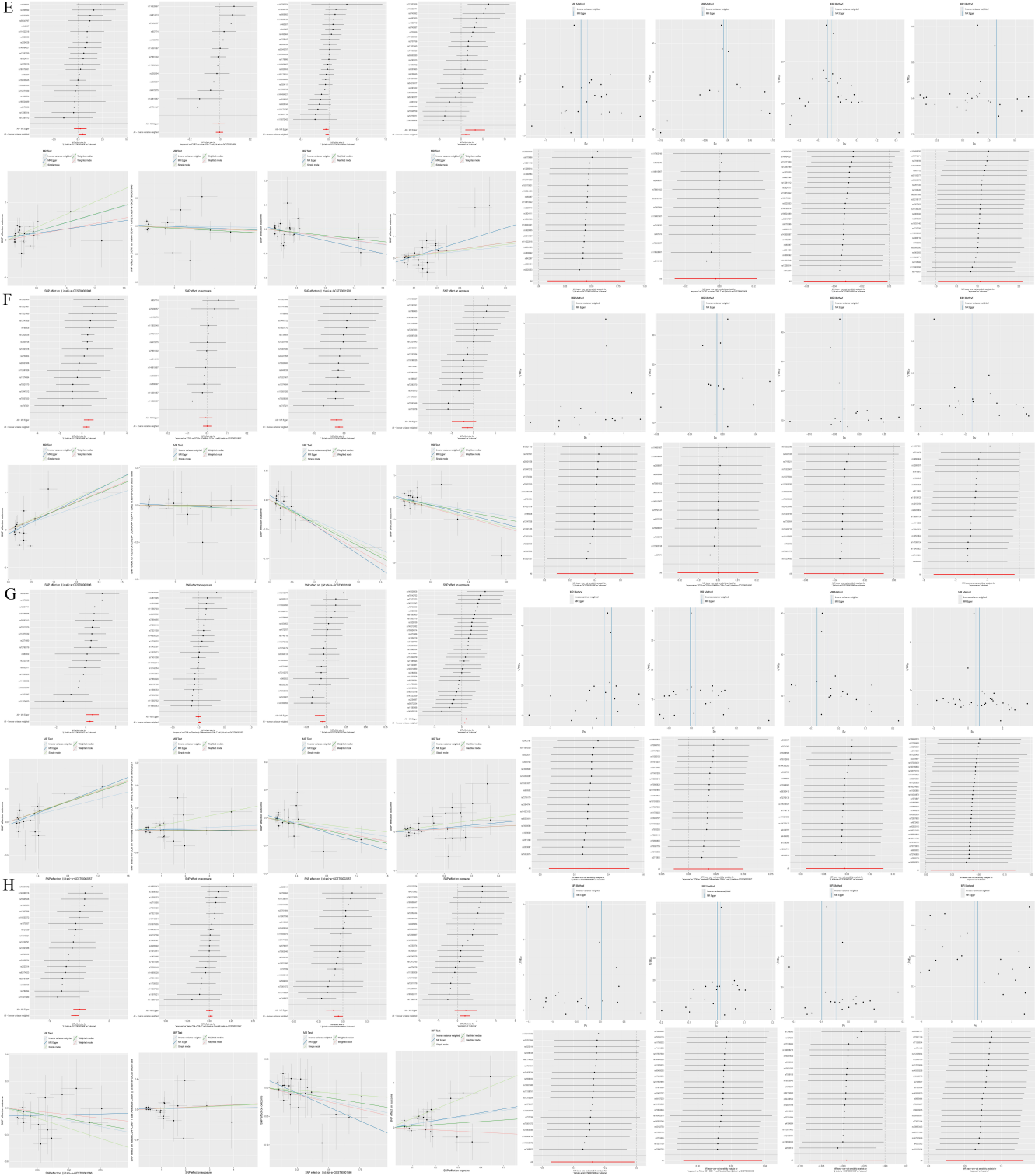

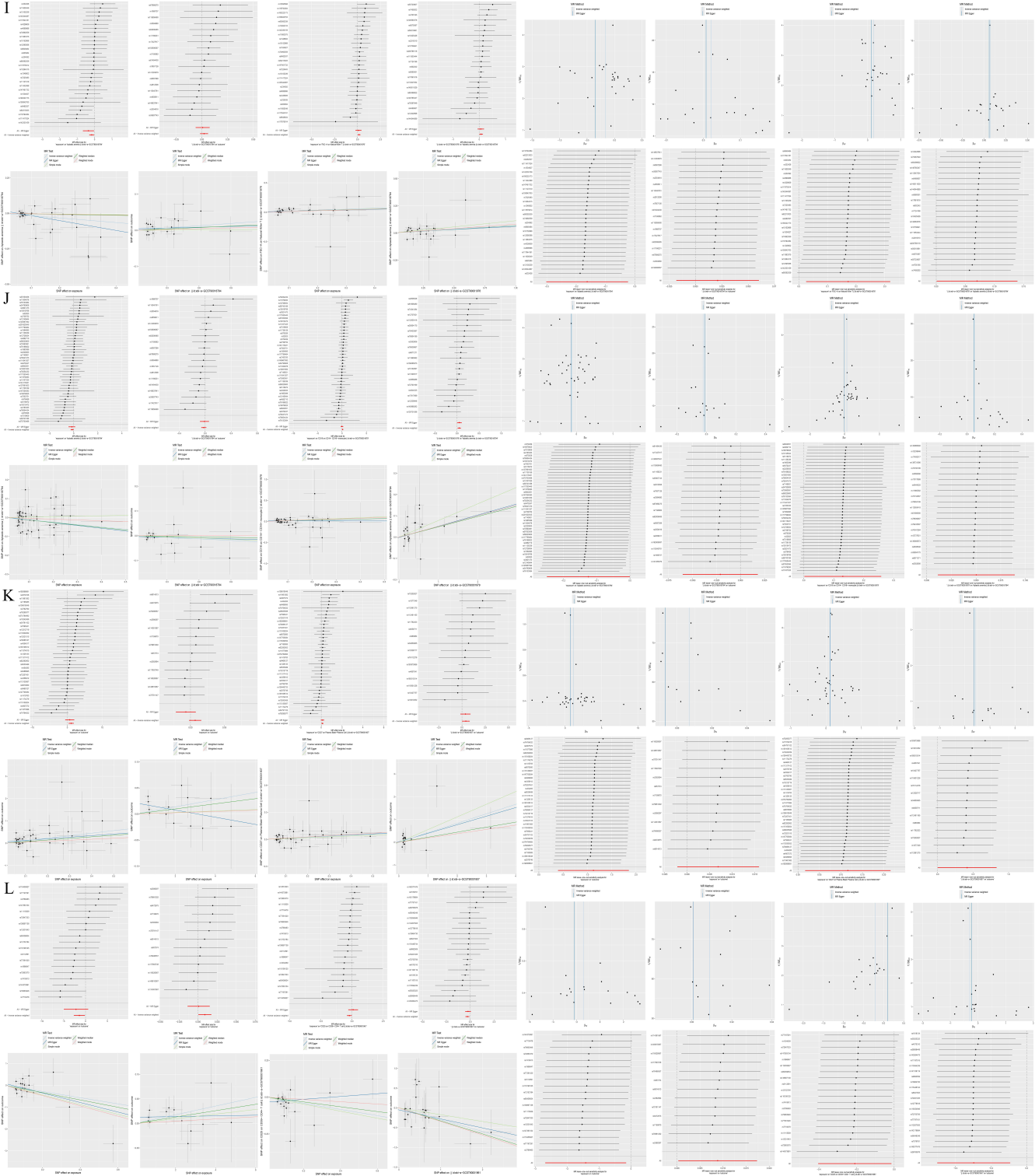

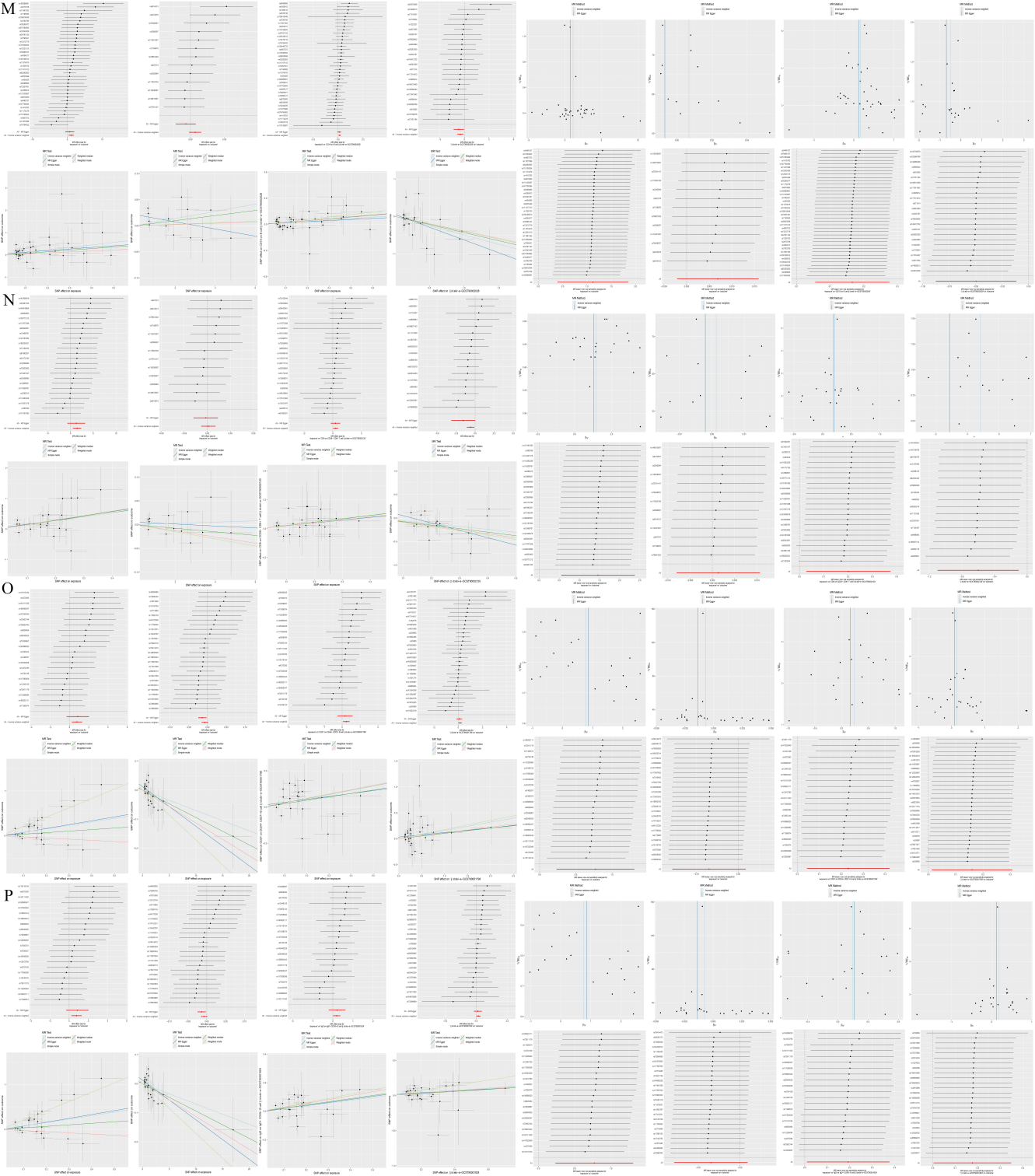

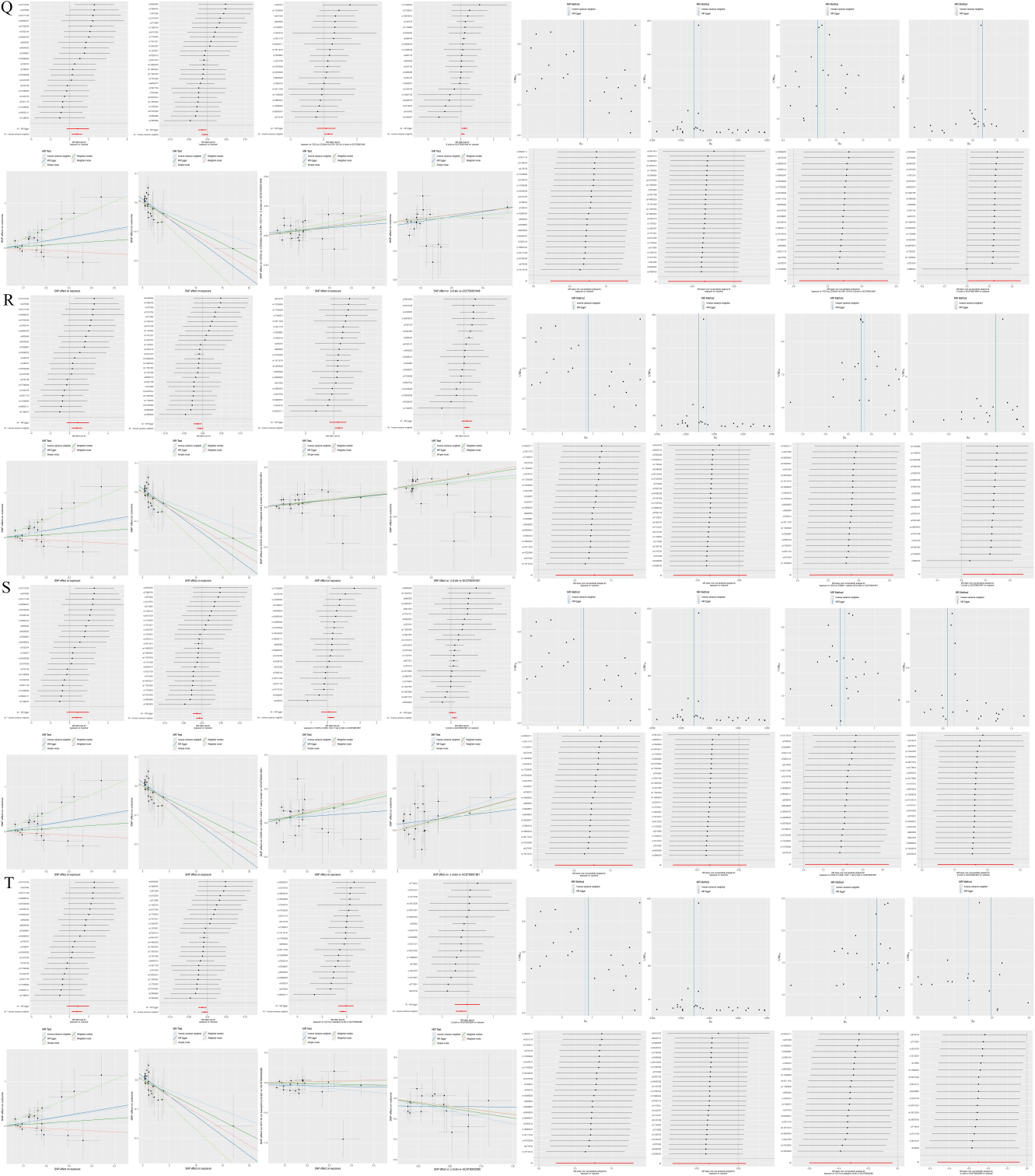

Supplement: Supplementary file 14 [file DataSheet6.pdf]
