# Supplementary material for: Identification of common hub genes and construction of immune regulatory networks in aplastic anemia, myelodysplastic syndromes, and acute myeloid leukemia
Source: Front Immunol. 2025 May 8;16:1547289. doi: 10.3389/fimmu.2025.1547289 (PMC12095185; doi:10.3389/fimmu.2025.1547289)
Supplement: Supplementary file 10 [file DataSheet2.pdf]

# 细胞 STR 峰图

Applied Biosystems

GeneMapper Software 6

20230906

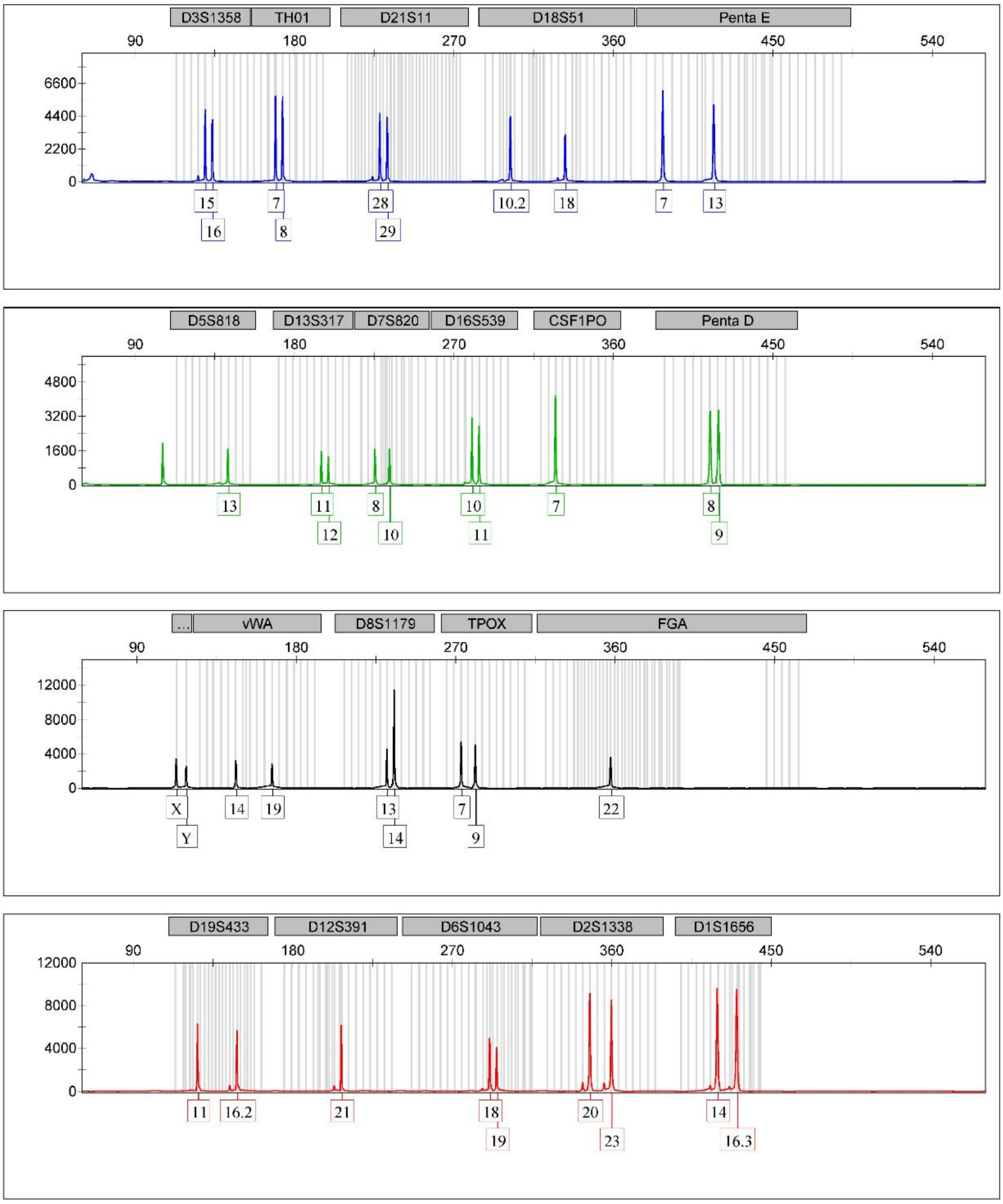


Wed Sep 06,2023 02:40PM, PDT

Printed by: Administrator

赛库生物 CELLCOOK

Product Information

# KG-1a

## Human myeloid leukemia cells

(CellCook cat:CC1913)

- Morphological characteristics: Lymphoblast-like
- Growth characteristics: Suspension
- Species: Human-derived
- Tissue origin: Bone marrow
- Disease: Acute myeloid leukemia

## Specifications and Storage

Regular delivery:
T25 culture flask, 1×10⁶ cells.
Please place the living cells in the cell incubator in a timely manner (at 37° C, with 5% $CO_2$).

For cryopreserved cell stocks during delivery:
Two tubes of the same batch, with $1\times10^6$ cells per tube.
Please store them in liquid nitrogen (-196° C) in a timely manner after receiving them.

## Range of application

This product is limited to scientific research only.


- Guangzhou Cellcook Biotech Co.,Ltd
- Tel:020-89449936
- Email:info@cel lcook .com
- www.cellcook.com


赛库公众号

赛库微信小程序

## culture conditions

RPMI 1640(Gibco cat:11875 Or the same formula.）20％Fetal Bovine Serum

Recommended Culture Reagents

Basal Medium:

RPMI 1640(Ce11Cook: CM 2017 OR Gib co cat: 11875 or the same formulation）

blood serum:
South American Fetal Bovine Serum(CellCook cat:CM1002L)

Additive:

\

Matched Complete Culture Medium(CellCook cat:CC1913M)

Sub - culturing Method: Sub - culture at a ratio of 1:3 (ratio of culture area); maintain the cell concentration at $2\times10^5$ - $1\times10^6$ cells/ml.
Sub - culturing Mode: Collect by centrifugation (1000 rpm, 5 minutes).
Medium - Changing Frequency: Change the medium once every 2 - 3 days.
Doubling Time: ~ 45 hours (CLS); ~ 50 hours (DSMZ)
Cryopreservation Solution Formula: RPMI 1640 + 20% FBS + 10% DMSO

Difficulty Level: ++

Key Points of Cultivation: It is necessary to control the cell density.

Characteristics: The KG1 cell line was established by H.P. Koeffler and D.W. Golde. Bone marrow was aspirated from a 59 - year - old Caucasian male who had erythroleukemia and then developed acute myelogenous leukemia. The KG1 cells are morphologically similar to acute myelogenous leukemia, showing significant polymorphism, with myeloblasts and myelocytes predominating. A small number of cells are mature granulocytes, and there are also trace amounts of macrophages and eosinophils. After 35 passages, morphological characteristics different from those of the parent cell line are exhibited. The subtype KG - 1a is composed of undifferentiated promyelocytes. Compared with KG - 1 cells, KG - 1a does not respond to colony - stimulating factors in soft - agar culture and does not express LA - like antigens. At the same time, it is resistant to phorbol - ester - induced macrophage differentiation, and phorbol - ester does not cause cell proliferation either.

STR Locus Information:

| STR Profile | AMEL | CSF1PO | D13S317 | D16S539 | D5S818 | D7S820 | TH01 | TPOX | vWA |
|---|---|---|---|---|---|---|---|---|---|
| KG-1a | X, Y | 7 | 11, 12 | 10, 11 | 13 | 8, 10 | 7, 8 | 7, 9 | 14, 19 |
