# Supplementary material for: Identification of common hub genes and construction of immune regulatory networks in aplastic anemia, myelodysplastic syndromes, and acute myeloid leukemia
Source: Front Immunol. 2025 May 8;16:1547289. doi: 10.3389/fimmu.2025.1547289 (PMC12095185; doi:10.3389/fimmu.2025.1547289)
Supplement: Supplementary file 12 [file DataSheet4.pdf]

To optimize gene expression, a G4S-linker sequence (GGTGGGGGCGGC) was inserted at the junction. Restriction sites were added to the primer sequences at both ends, and 2 or 3 protective bases were added at the end. Stop codons were removed. After the fusion gene was spliced into the plasmid, we checked in SnapGene whether the number of bases was a multiple of 3 to avoid potential decoding errors. If issues were detected, additional bases were randomly added downstream of MAP2K7.In the first round of PCR, upstream (F) and downstream (R) primers were added to gene templates including POLG and MAP2K7 (from the normal control cells). The POLG downstream primer and the MAP2K7 upstream primer both consisted of the POLG downstream partial sequence, the G4S sequence, and the MAP2K7 upstream partial sequence. Gel extraction was performed to obtain the fragments joined by overlap extension PCR. In the second round, the fragments obtained were subjected to 10 cycles of PCR without primers. After adding POLG upstream and MAP2K7 downstream primers respectively, we performed overlap extension PCR and linked two genes to form a fusion gene. Subsequently, the expression plasmid was constructed by digesting the vector with HindIII/NdeI and performing homologous recombination. Firstly, gel recovery was performed by electrophoresis, followed by homologous recombination between multiple fragments and vectors, allowing the expression vector to connect to the fusion gene to construct a recombinant plasmid.

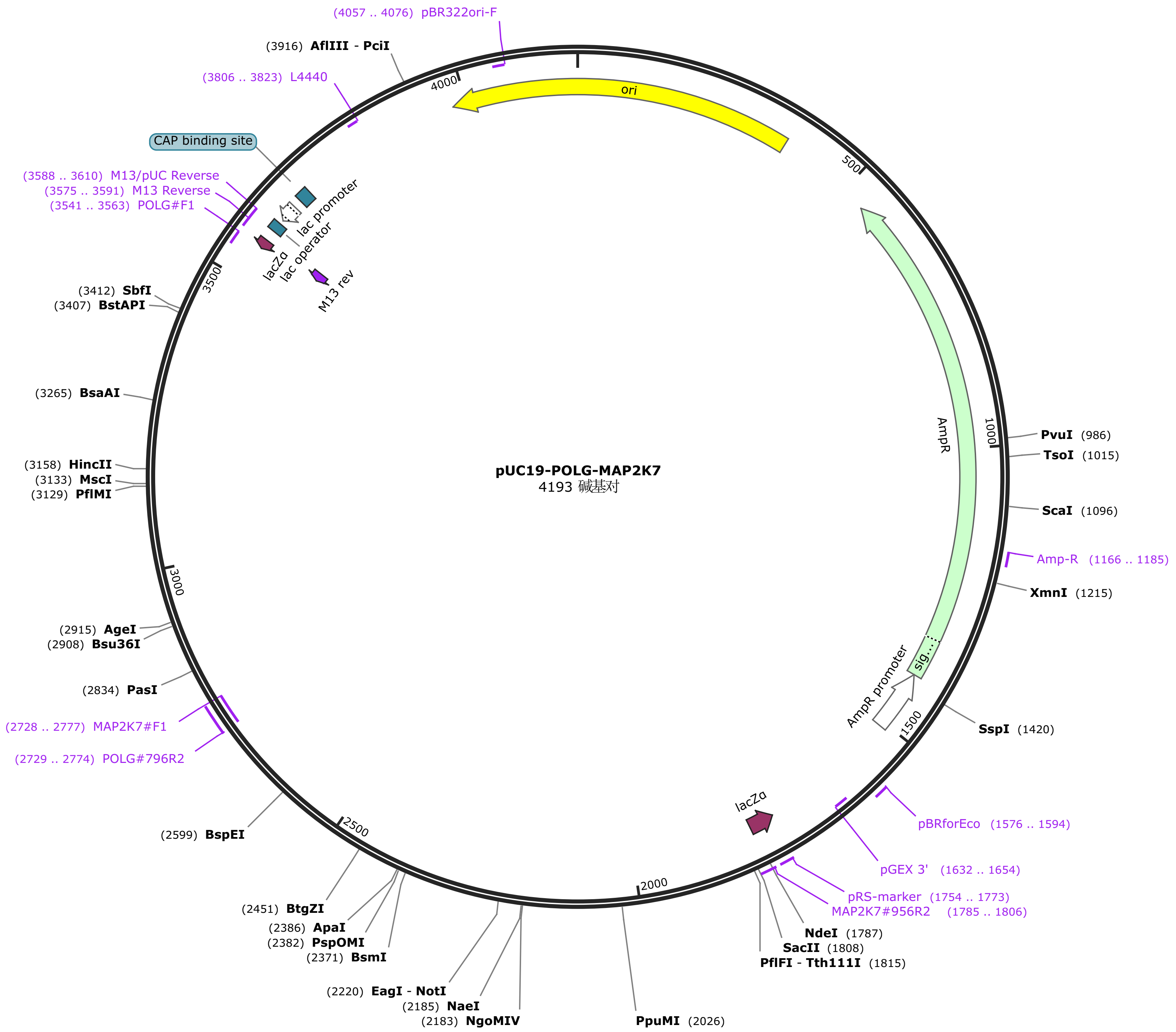

Created with SnapGene®
