# Supplementary material for: Identification of common hub genes and construction of immune regulatory networks in aplastic anemia, myelodysplastic syndromes, and acute myeloid leukemia
Source: Front Immunol. 2025 May 8;16:1547289. doi: 10.3389/fimmu.2025.1547289 (PMC12095185; doi:10.3389/fimmu.2025.1547289)
Supplement: Supplementary file 13 [file DataSheet5.pdf]

Beyotime

Support: +86-400-1683301 / info-intl@beyotime.com
Orders: order-intl@beyotime.com
Tel: +86-400-1683301 / +86-800-8283301
Add: 1500 Xinfei Rd, Songjiang, Shanghai, China
Web: www.beyotime.com

Human MIF ELISA Kit

Cat. No.	Product Name	Pack Size
PM715	Human MIF ELISA Kit	96T

Description:

- Beyotime's Human MIF ELISA Kit (Human Macrophage Migration Inhibitory Factor Enzyme-Linked ImmunoSorbent Assay Kit) provides a specific and highly sensitive method for quantification of MIF in human serum, plasma, and cell culture supernatant.
- This product has high sensitivity, high specificity and good reproducibility. Multiple repeated assays show that the minimum detection amount is 18pg/ml, and there is no cross-reactivity with human IL-1, IL-6, IL-8, ICAM, SDF-1α, SDF-1βI, mouse MIF, etc. The intra-plate and inter-plate coefficients of variation are less than 10%.
- Macrophage Migration Inhibitory Factor (MIF or MMIF), one of the first cytokines identified, was initially thought to inhibit macrophage migration at will, but was later shown to have chemotactic effects on macrophages. Further studies revealed that MIF is not a typical ligand for cytokine receptors. MIF is now considered to be a protein with cytokine functions only, also known as micro chemokines, and is an important regulator of intrinsic immune responses, with important roles in pathological processes such as inflammation, autoimmune diseases and cancer. The human *MIF* gene encodes a secreted MIF protein comprising 115 amino acid residues with a molecular weight of 12.5kDa. MIF usually exists as a homodimer, but may also exist as a monomer or a dimer. MIF has some reciprocal isomerase and oxidoreductase activities, but the biological significance of MIF as a protease has not yet been determined.
- A variety of cells in immune system express MIF, such as monocytes, macrophages, T cells, B cells, neutrophils, and atherosclerotic foam cells. It is also expressed in endothelial cells, smooth muscle cells and non-immune tissues such as atherosclerotic, tumor neovascularization and endocrine tissues. Activation of some MIFs requires CD74, with CD44 as a co-receptor. Other MIF binds to cytokine receptors such as CXCR4 or CXCR7 and exerts cytokine-like functions. MIF also binds to intracellular Jab1 and attenuates Jab1-mediated G1 phase cell cycle arrest.
- MIF regulates acute or chronic inflammatory responses and is secreted when stimulated by endotoxins or pro-inflammatory cytokines. MIF has paracrine and autocrine stimulatory effects on cell production and survival, promotes inflammatory cytokine production, and also inhibits the function of immunosuppressive glucocorticoids. Knockout of the *MIF* gene suppresses the development of endotoxemia and infectious shock in model mice. In humans, MIF level in blood is increased in the presence of sepsis or infectious shock and acute respiratory distress syndrome. In addition, elevated MIF expression is associated with increased vascular density and risk of tumor recurrence, and MIF is thought to increase the risk of cancer in patients with chronic inflammatory conditions.
- This kit employs the double-antibody sandwich ELISA for quantification analysis of human MIF in samples (Figure 1). The monoclonal antibodies against human MIF are precoated on the plate as capture antibodies and when a standard or sample is added, the human MIF binds to the capture antibody. The biotin-conjugated human MIF antibody is then added and binds to human MIF on the plate to form a sandwiched immune complex. After that, HRP-labeled Streptavidin is added and binds to the sandwich immune complex through the specific interaction between biotin and streptavidin. Finally, the chromogenic reaction is initiated by the addition of TMB Solution. TMB produces a deep blue color during the enzymatic degradation of hydrogen peroxide by HRP, and the addition of Stop Solution gives a clear yellow color that absorbs at 450nm. The A450 value is directly proportional to the concentration of human MIF in samples which can be calculated from the standard curve generated in the same assay.

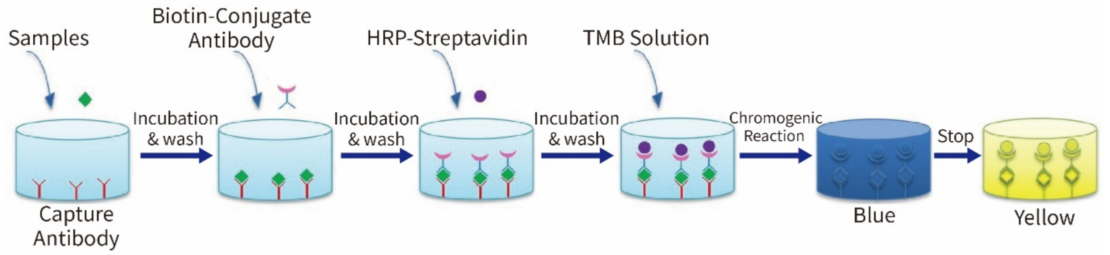

Figure 1. Schematic diagram of double-antibody sandwich ELISA

- This kit is sufficient for 96 assays.

Packing List:

Item	Component	Quantity
PM715-1	96-well Strip-well Plate Coated with Human MIF Antibody	8 wells × 12 strips
PM715-2	Assay Buffer	5ml
PM715-3	Standard Dilution Buffer (5X)	10ml
PM715-4	Human MIF Standard	2-4 bottles
PM715-5	Biotin-conjugated Human MIF Antibody	10ml
PM715-6	HRP-labeled Streptavidin	10ml
PM715-7	Wash Buffer (20X)	30ml
PM715-8	TMB Solution	10ml
PM715-9	Stop Solution	5ml
PM715-10	Adhesive Films (transparent)	2
PM715-11	Adhesive Films (white)	2
Manual	—	1 copy

Storage Conditions:

Store the Standard at 4ºC for up to 1-2 weeks, or -20ºC for up to 6 months. Store the other components in this kit at 4ºC for up to 6 months.

Precautions:

- The standard is generally lyophilized powder. Check the instructions labeled on the standard vial to prepare the standard solution.
- Crystal precipitation may exist in Wash Buffer (20X) at low temperatures. Please dissolve it completely using a water bath at room temperature prior to use.
- Standard solution should be prepared freshly. Discard the rest after use.
- Avoid oxidizer and metal contamination that cause the invalidation of TMB Solution.
- Change pipette tips between different samples and liquids to prevent contamination and incorrect loading volumes.
- Do not mix or interchange reagents from different kit lots.
- It is particularly important to perform sufficient mixing of reactions to ensure an accurate result. Please shake the 96-well plate gently after the addition of reagents.
- Most procedures of this experiment should be performed at room temperature (25-28ºC). Temperature lower than 25ºC will result in a significant decrease in the absorbance value of reactions.
- The washing process is very important. Insufficient wash will result in reduced accuracy and increased experimental errors.
- Run all standards, controls, and samples in duplicate.
- Avoid the formation of air bubbles when adding the sample.
- This product is for R&D only. Not for drug, household, or other uses.
- For your safety and health, please wear a lab coat and disposable gloves during the operation.

Instructions for Use:

1. **Preparation of the sample.**

   a. **Cell supernatant:** Centrifuge cell cultures at 100-500×g for 5 minutes to collect the supernatant.

   b. **Serum:** Leave the whole blood undisturbed at room temperature for 30 minutes to 2 hours. After the whole blood clots, collect the yellow supernatant (serum) by centrifuging the whole blood at 1000-2000×g for 10 minutes at 4ºC, and keep the serum on ice.

   ***Note:** Do Not add any preservatives or anticoagulants to serum.*

   c. **Plasma:** Add heparin or EDTA anticoagulant to whole blood and place on ice after mix. After centrifuging at 1000-2000×g for

10 minutes at 4ºC, collect the yellow supernatant (plasma) and keep it on ice.

Note 1: *If the samples cannot be analyzed immediately, make aliquots and store them at -20ºC or -80ºC. Avoid repeated freeze-thaw.*

Note 2: *Samples should be clear and transparent. Remove any particulates from samples by centrifuging before being analyzed.*

Note 3: *Do not use hemolyzed, hyperlipidemia, or contaminated samples for analysis.*

Note 4: *Serum or plasma samples may need to be properly diluted with Assay Buffer before the assay.*

2. Preparation of the kit.

a. Thaw the reagents and equilibrate to room temperature (25-28ºC) prior to use. Store the reagents at 4ºC immediately after use.

b. Prepare an appropriate amount of 1X Standard Dilution Buffer by diluting the Standard Dilution Buffer (5X) with ddH_2O or deionized water. For example, mix 10ml of Standard Dilution Buffer (5X) with 40ml of water.

c. Prepare an appropriate amount of 1X Wash Buffer by diluting the Wash Buffer (20X) with ddH_2O.

d. Reconstitute Standard to 2000pg/ml with 1X Standard Dilution Buffer according to the instructions labeled on the standard vial. Mix gently and incubate for 15min at room temperature. Gently pipette the contents several times to dissolve the standard completely. Generally, each concentration of standard is analyzed at least in duplicates (at least 2 wells) and 100μl is needed for each well. If one bottle of Standard is not enough, use more bottles of Standard, but the contents from different bottles need to be mixed before performing dilutions.

e. Add 250μl of 1X Standard Dilution Buffer to each of 5 tubes and make serial dilutions as shown in Figure 2 to obtain 5 concentrations: 1/2 (1000pg/ml), 1/4 (500pg/ml), 1/8 (250pg/ml), 1/16 (125pg/ml), 1/32 (62.5pg/ml). Mix thoroughly between steps.

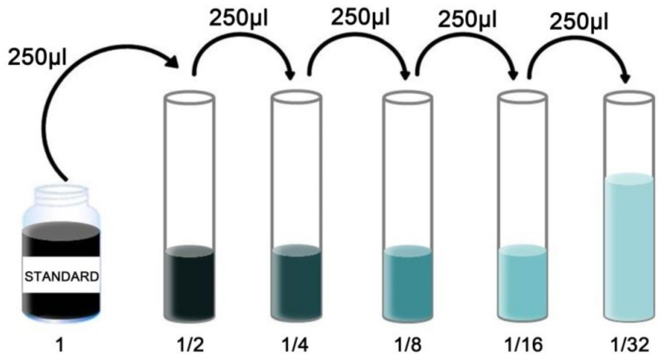

Figure 2. Dilution diagram for preparation of different concentrations of Standard.

3. Perform ELISA.

a. Determine the number of pre-coated 8-well strips required for the experiment. Insert the strips in a frame for use. Re-bag any unused strips and store them at 4ºC.

b. Prepare the Standard freshly and plot a standard curve for every run. Set up the blank control by only adding TMB Solution and Stop Solution.

c. Add 100μl of samples or standards to each well. Cover wells with Adhesive Films (transparent), and incubate for 2 hours at room temperature.

Note: *For the assay of serum or plasma samples, dilution ratio varies from sample to sample, generally in the range of 1:*5-1:20. *If there is no clear range, it is recommended to start from 1:10 dilution. Please add 50 μl of Assay Buffer first, and then add 50 μl of the sample diluted with 1X Standard Dilution Buffer. If the sample concentration is too high and beyond the detection range, increase the dilution factor and reanalyze. It is necessary to record the dilution factor of the sample.*

d. Thoroughly aspirate the solution by gently lowering a pipette tip into the bottom of each well and wash wells 5 times with 300μl of 1X Wash Buffer. Allow the buffer to stand for 15-30 seconds before aspiration. After the last wash, invert the strip and tap dry on absorbent tissue.

e. Add 100μl of Biotin-conjugated Antibody to each well (***Note:*** *the antibody can be used directly without dilution*). Cover wells with Adhesive Films (transparent) and incubate for 1 hour at room temperature.

f. Thoroughly aspirate the solution and wash wells 5 times with 300μl of 1X Wash Buffer, as described in step 3d.

g. Add 100μl of HRP-labeled Streptavidin to each well (***Note:*** *the HRP-labeled Streptavidin can be used directly without dilution*), cover wells with Adhesive Films (white), and incubate at room temperature for 20 minutes in the dark. If the room temperature is low, prolong the incubation time as appropriate.

h. Thoroughly aspirate the solution and wash wells 5 times with 300μl of 1X Wash Buffer, as described in step 3d.

i. Add 100μl of TMB Solution to each well, cover wells with Adhesive Films (white), and incubate at room temperature for 15-20 minutes in the dark. If the room temperature is low, prolong the incubation time until the standard sample exhibits significant color change. If the concentration of target protein in sample is high, color change will occur soon.

j. Add 50μl of Stop Solution to each well. Read the absorbance at 450nm immediately after mixing.

4. Analysis.

a. Calculate the average A450 value for each standard and sample. Duplicates should be within 20 percent of the mean value.

b. Subtract the A450 value of blank control from the A450 values of standards and samples (This step can be omitted if there is no blank control).

c. Generate the standard curve by plotting the concentrations of standard on the abscissa and their corresponding A450 values on the ordinate (Figure 3).

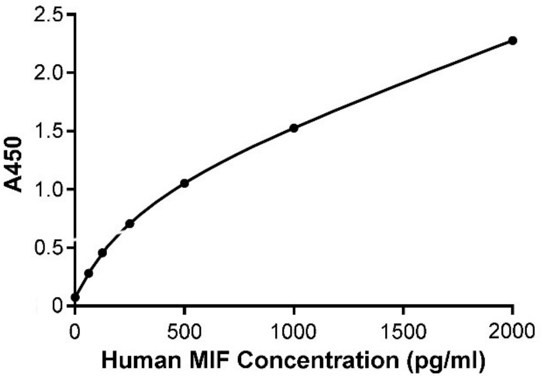

Figure 3. The standard curve of Beyotime's Human MIF ELISA Kit (PM715). This figure is for reference only, which may vary due to different experimental conditions.

d. Determine the concentrations of human MIF in samples from the standard curve based on their A450 values.

Note: *Dilute samples producing signals greater than the upper limit of the standard curve in Standard Dilution Buffer and reanalyze. Multiply the concentration by the dilution factor.*

Related Products:

Cat. No.	Product Name	Pack Size
PM715	Human MIF ELISA Kit	96T

Version 2018.11.21

Beyotime

Support: +86-400-1683301 / info-intl@beyotime.com
Orders: order-intl@beyotime.com
Tel: +86-400-1683301 / +86-800-8283301
Add: 1500 Xinfei Rd, Songjiang, Shanghai, China
Web: www.beyotime.com

Human LIF ELISA Kit

Cat. No.	Product Name	Pack Size
PL695	Human LIF ELISA Kit	96T

Description:

- Beyotime's Human LIF ELISA Kit (Human LIF Enzyme-Linked ImmunoSorbent Assay Kit) provides a specific and highly sensitive method for quantification of LIF in human serum, plasma, and cell culture supernatant.
- This product has high detection sensitivity, high specificity and good reproducibility. Multiple repeated assays show a minimum detection amount of 232pg/ml, no cross-reactivity with human HIC5 and LIM1, and 33% cross-reactivity with mouse LIF. The intra-plate and inter-plate coefficients of variation are both less than 10%.
- Leukemia inhibitory factor (LIF) is a member of the cytokine IL-6 family. It is a polypeptide with a molecular weight of 38-67 kDa and was originally identified as a proliferation inhibitor and differentiation inducer in the mouse M1 myeloid leukemia cell line. Mature LIF comprises 180 amino acid residues with multiple potential N-bond and O-bond glycosylation sites and six conserved cysteines, forming three intramolecular disulfide bonds. Mature mouse LIF shares 78% homology in amino acid sequence with human LIF, indicating that LIF is highly conserved biologically.
- LIF binds to the LIF receptor (LIF R) which is a member of the hematopoietin receptor superfamily. When expressed alone, LIF R binds LIF with low affinity. When expressed simultaneously with gp130, a signaling subunit shared by the cytokine IL-6 receptors, a high-affinity complex is formed between LIF, LIF R and gp130, and triggers downstream signaling events via the JAK/STAT, PI3K and MAPK signaling pathways.
- Cells known to express LIF include activated T cells, monocytes, astrocytes, osteoblasts, keratinocytes, regenerating skeletal muscle, mast cells, and fibroblasts. Functionally, LIF is involved in many processes including stem cell maintenance, reproduction, development, hematopoiesis, bone metabolism, skeletal muscle regeneration, and inflammation. Studies have also shown that LIF protects cardiomyocytes during ischemia-reperfusion and enhances repair after ischemic stroke by protecting neuronal cells from reactive oxygen species (ROS) damages and maintaining large numbers of neuronal stem cells. LIF can be detected in a variety of body fluids Elevated LIF concentrations in serum correlates with hematologic malignancies, while elevated LIF concentrations in bronchoalveolar lavage fluid correlates with increased inflammatory markers. Peripheral blood leukocyte counts correlate with elevated LIF concentrations in patients with rheumatoid arthritis.
- This kit employs the double-antibody sandwich ELISA for quantification analysis of human LIF in samples (Figure 1). The monoclonal antibodies against human LIF are precoated on the plate as capture antibodies and when a standard or sample is added, the human LIF binds to the capture antibody. The biotin-conjugated human LIF antibody is then added and binds to human LIF on the plate to form a sandwiched immune complex. After that, HRP-labeled Streptavidin is added and binds to the sandwich immune complex through the specific interaction between biotin and streptavidin. Finally, the chromogenic reaction is initiated by the addition of TMB Solution. TMB produces a deep blue color during the enzymatic degradation of hydrogen peroxide by HRP, and the addition of Stop Solution gives a clear yellow color that absorbs at 450nm. The A450 value is directly proportional to the concentration of human LIF in samples which can be calculated from the standard curve generated in the same assay.

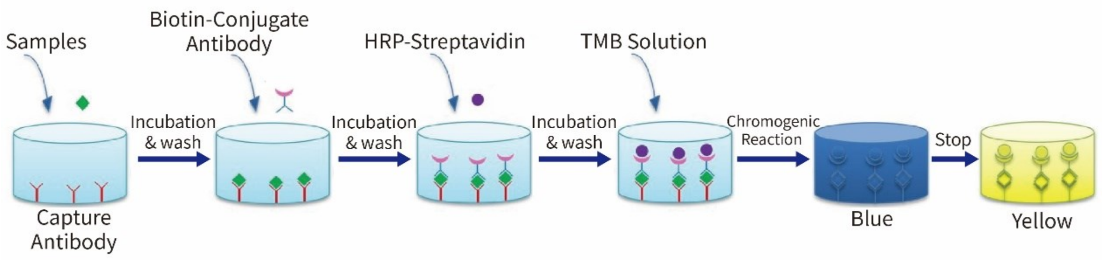

Figure 1. Schematic diagram of double-antibody sandwich ELISA

- This kit is sufficient for 96 assays.

Packing List:

Item	Component	Quantity
PL695-1	96-well Strip-well Plate Coated with Human LIF Antibody	8 wells×12 strips
PL695-2	Assay Buffer	5ml
PL695-3	Standard Dilution Buffer	10ml
PL695-4	Human LIF Standard	2-4 bottles
PL695-5	Biotin-conjugated Human LIF Antibody	10ml
PL695-6	HRP-labeled Streptavidin	10ml
PL695-7	Wash Buffer (20X)	30ml
PL695-8	TMB Solution	10ml
PL695-9	Stop Solution	5ml
PL695-10	Adhesive Films (transparent)	2
PL695-11	Adhesive Films (white)	2
Manual	—	1 copy

Storage Conditions:

Store the Standard at 4ºC for up to 1-2 weeks, or -20ºC for up to 6 months. Store the other components in this kit at 4ºC for up to 6 months.

Precautions:

- The standard is generally lyophilized powder. Check the instructions labeled on the standard vial to prepare the standard solution.
- Crystal precipitation may exist in Wash Buffer (20X) at low temperatures. Please dissolve it completely using a water bath at room temperature prior to use.
- Standard solution should be prepared freshly. Discard the rest after use.
- Avoid oxidizer and metal contamination that cause the invalidation of TMB Solution.
- Change pipette tips between different samples and liquids to prevent contamination and incorrect loading volumes.
- Do not mix or interchange reagents from different kit lots.
- It is particularly important to perform sufficient mixing of reactions to ensure an accurate result. Please shake the 96-well plate gently after the addition of reagents.
- Most procedures of this experiment should be performed at room temperature (25-28ºC). Temperature lower than 25ºC will result in a significant decrease in the absorbance value of reactions.
- The washing process is very important. Insufficient wash will result in reduced accuracy and increased experimental errors.
- Run all standards, controls, and samples in duplicate.
- Avoid the formation of air bubbles when adding the sample.
- This product is for R&D only. Not for drug, household, or other uses.
- For your safety and health, please wear a lab coat and disposable gloves during the operation.

Instructions for Use:

1. **Preparation of the sample.**
   a. **Cell supernatant:** Centrifuge cell cultures at 100-500×g for 5 minutes to collect the supernatant.
   b. **Serum:** Leave the whole blood undisturbed at room temperature for 30 minutes to 2 hours. After the whole blood clots, collect the yellow supernatant (serum) by centrifuging the whole blood at 1000-2000×g for 10 minutes at 4ºC, and keep the serum on ice.

   Note: *Do Not add any preservatives or anticoagulants to serum.*

   c. **Plasma:** Add heparin or EDTA anticoagulant to whole blood and place on ice after mix. After centrifuging at 1000-2000×g for 10 minutes at 4ºC, collect the yellow supernatant (plasma) and keep it on ice.

   Note 1: *If the samples cannot be analyzed immediately, make aliquots and store them at -20ºC or -80ºC. Avoid repeated freeze-*

thaw.

Note 2: *Samples should be clear and transparent. Remove any particulates from samples by centrifuging before being analyzed.*

Note 3: *Do not use hemolyzed, hyperlipidemia, or contaminated samples for analysis.*

Note 4: *Serum or plasma samples may need to be properly diluted with Assay Buffer before the assay.*

2. **Preparation of the kit.**
   a. Thaw the reagents and equilibrate to room temperature (25-28ºC) prior to use. Store the reagents at 4ºC immediately after use.
   b. Prepare an appropriate amount of 1X Wash Buffer by diluting the Wash Buffer (20X) with ddH_2O.
   c. Reconstitute Standard to 1000pg/ml with Standard Dilution Buffer according to the instructions labeled on the standard vial. Mix gently and incubate for 15min at room temperature. Gently pipette the contents several times to dissolve the standard completely. Generally, each concentration of standard is analyzed at least in duplicates (at least 2 wells) and 100μl is needed for each well. If one bottle of Standard is not enough, use more bottles of Standard, but the contents from different bottles need to be mixed before performing dilutions.
   d. Add 250μl of Standard Dilution Buffer to each of 5 tubes and make serial dilutions as shown in Figure 2 to obtain 5 concentrations: 1/2 (500pg/ml), 1/4 (250pg/ml), 1/8 (125pg/ml), 1/16 (62.5pg/ml), 1/32 (31.25pg/ml). Mix thoroughly between steps.

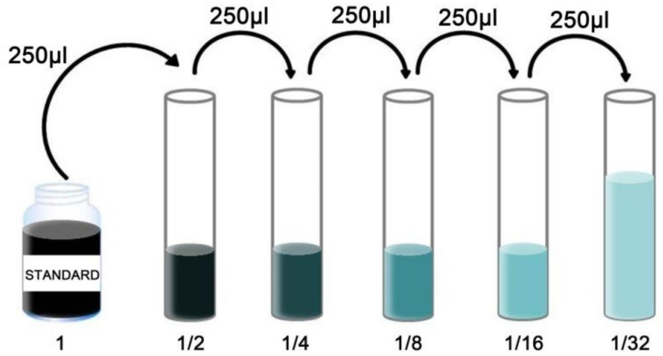

Figure 2. Dilution diagram for preparation of different concentrations of Standard.

3. **Perform ELISA.**
   a. Determine the number of pre-coated 8-well strips required for the experiment. Insert the strips in a frame for use. Re-bag any unused strips and store them at 4ºC.
   b. Prepare the Standard freshly and plot a standard curve for every run. Set up the blank control by only adding TMB Solution and Stop Solution.
   c. Add 100μl of samples or standards to each well. Cover wells with Adhesive Films (transparent), and incubate for 2 hours at room temperature.

   Note: *For the assay of serum or plasma samples, add 50 μl of Assay Buffer followed by 50 μl of sample. At this point, the sample is diluted 2 times. If the sample concentration is too high and beyond the detection range, add 50 μl of Assay Buffer first, and then add 50 μl of the diluted sample. It is necessary to record the dilution factor of the sample.*

   d. Thoroughly aspirate the solution by gently lowering a pipette tip into the bottom of each well and wash wells 5 times with 300μl 1X Wash Buffer. Allow the buffer to stand for 15-30 seconds before aspiration. After the last wash, invert the strip and tap dry on absorbent tissue.
   e. Add 100μl of Biotin-conjugated Antibody to each well (***Note:*** *the antibody can be used directly without dilution*). Cover wells with Adhesive Films (transparent) and incubate for 1 hour at room temperature.
   f. Thoroughly aspirate the solution and wash wells 5 times with 300μl of 1X Wash Buffer, as described in step 3d.
   g. Add 100μl of HRP-labeled Streptavidin to each well (***Note:*** *the HRP-labeled Streptavidin can be used directly without dilution*), cover wells with Adhesive Films (white), and incubate at room temperature for 20 minutes in the dark. If the room temperature is low, prolong the incubation time as appropriate.
   h. Thoroughly aspirate the solution and wash wells 5 times with 300μl of 1X Wash Buffer, as described in step 3d.
   i. Add 100μl of TMB Solution to each well, cover wells with Adhesive Films (white), and incubate at room temperature for 15-20 minutes in the dark. If the room temperature is low, prolong the incubation time until the standard sample exhibits significant color change. If the concentration of target protein in sample is high, color change will occur soon.
   j. Add 50μl of Stop Solution to each well. Read the absorbance at 450nm immediately after mixing.

4. **Analysis.**
   a. Calculate the average A450 value for each standard and sample. Duplicates should be within 20 percent of the mean value.
   b. Subtract the A450 value of blank control from the A450 values of standards and samples (This step can be omitted if there is no blank control).
   c. Generate the standard curve by plotting the concentrations of standard on the abscissa and their corresponding A450 values on the ordinate (Figure 3).

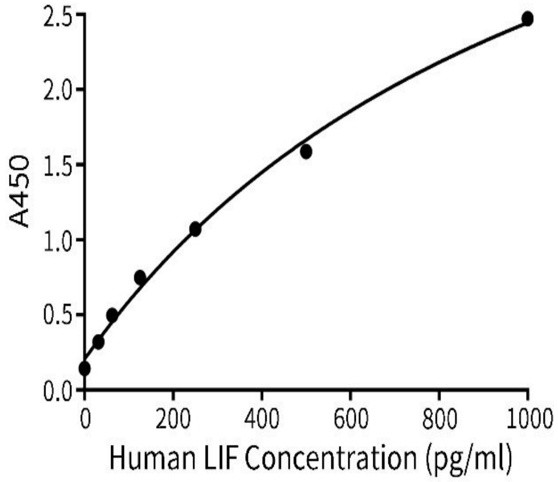

Figure 3. The standard curve of Beyotime's Human LIF ELISA Kit (PL695). This figure is for reference only, which may vary due to different experimental conditions.

   d. Determine the concentrations of human LIF in samples from the standard curve based on their A450 values.

   Note: *Dilute samples producing signals greater than the upper limit of the standard curve in Standard Dilution Buffer and reanalyze. Multiply the concentration by the dilution factor.*

Related Products:

Cat. No.	Product Name	Pack Size
PI600	Human IL-11 ELISA Kit	96T
PL695	Human LIF ELISA Kit	96T

Version 2021.07.28

Beyotime

Support: +86-400-1683301 / info-intl@beyotime.com
Orders: order-intl@beyotime.com
Tel: +86-400-1683301 / +86-800-8283301
Add: 1500 Xinfei Rd, Songjiang, Shanghai, China
Web: www.beyotime.com

Human HGF ELISA Kit

Cat. No.	Product Name	Pack Size
PH385	Human HGF ELISA Kit	96T

Description:

- Beyotime's Human HGF ELISA Kit (Human Hepatocyte Growth Factor Enzyme-Linked ImmunoSorbent Assay Kit) provides a specific and highly sensitive method for quantification of HGF in human serum, plasma, and cell culture supernatant.
- This product has high sensitivity, high specificity and good reproducibility. The multiple repeated assays show that the minimum detection amount is 112.7 pg/ml and there is no cross-reactivity with human EGF, G-CSF, GMCSF, HGF R, M-CSF, PDGF-AA, PDGF-AB, PDGF-BB, TGF-β1, VEGF, and mouse HGF. The intra-plate and inter-plate coefficients of variation are both less than 10%.
- Hepatocyte Growth Factor (HGF), also known as scatter factor, hepatopoietin A, and mammary growth factor, is a pleiotropic glycoprotein involved in the regulation of growth and migration of a variety of cells. The HGF molecule has an N-terminal PAN/APPLE-like region, four Kringle regions and a catalytic serine protease-like region. HGF is normally secreted as an inactive single-chain precursor peptide that can be activated after the excision by serine protease in damage tissues. Active HGF is a heterodimer composed of a N-terminal alpha chain (60 kDa) and a C-terminal beta chain (30 kDa) linked by disulfide bonds. In pathological conditions such as liver injury, acute renal failure, myocardial weakness, type I diabetes and tumors, HGF level in blood is significantly increased. Human HGF has 91-94% amino acid homology with bovine, canine, feline, mouse and rat HGF, with significant interspecies cross-reactivity.
- HGF is expressed mainly in fibroblasts, adipocytes, smooth muscle cells and endothelial cells, but the HGF receptor is mainly expressed in epithelial cells, suggesting that HGF is involved in regulating stroma-epithelial cell interactions in a paracrine manner. HGF induces proliferation and migration of many types of cells, including epithelial cells, hepatocytes, chondrocytes, keratinocytes and endothelial cells. HGF promotes the mitosis of tumor cells, but inhibits their proliferation in some cases. During organogenesis, tissue repair and angiogenesis, HGF can promote the morphogenesis of epithelial and endothelial cells, and its ability to regulate angiogenesis and promote mitosis of epithelial cells is important for the development of solid tumors. HGF also acts on pancreatic islet cells to promote their survival, proliferation and insulin production. In addition, HGF can suppress inflammatory responses by inducing dendritic cell tolerance, inhibiting T cell activation, suppressing IL-17 expression and inflammatory cell infiltration.
- This kit employs the double-antibody sandwich ELISA for quantification analysis of human HGF in samples (Figure 1). The monoclonal antibodies against human HGF are precoated on the plate as capture antibodies and when a standard or sample is added, the human HGF binds to the capture antibody. The biotin-conjugated human HGF antibody is then added and binds to human HGF on the plate to form a sandwiched immune complex. After that, HRP-labeled Streptavidin is added and binds to the sandwich immune complex through the specific interaction between biotin and streptavidin. Finally, the chromogenic reaction is initiated by the addition of TMB Solution. TMB produces a deep blue color during the enzymatic degradation of hydrogen peroxide by HRP, and the addition of Stop Solution gives a clear yellow color that absorbs at 450nm. The A450 value is directly proportional to the concentration of human HGF in samples which can be calculated from the standard curve generated in the same assay.

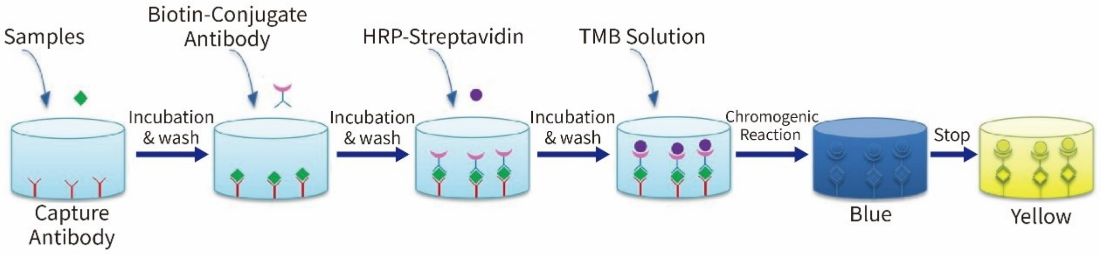

Figure 1. Schematic diagram of double-antibody sandwich ELISA

- This kit is sufficient for 96 assays.

Packing List:

Item	Component	Quantity
PH385-1	96-well Strip-well Plate Coated with Human HGF Antibody	8 wells × 12 strips
PH385-2	Assay Buffer	5ml
PH385-3	Standard Dilution Buffer	10ml
PH385-4	Human HGF Standard	2-4 bottles
PH385-5	Biotin-conjugated Human HGF Antibody	10ml
PH385-6	HRP-labeled Streptavidin	10ml
PH385-7	Wash Buffer (20X)	30ml
PH385-8	TMB Solution	10ml
PH385-9	Stop Solution	5ml
PH385-10	Adhesive Films (transparent)	2
PH385-11	Adhesive Films (white)	2
Manual	—	1 copy

Storage Conditions:

Store the Standard at 4ºC for up to 1-2 weeks, or -20ºC for up to 6 months. Store the other components in this kit at 4ºC for up to 6 months.

Precautions:

- The standard is generally lyophilized powder. Check the instructions labeled on the standard vial to prepare the standard solution.
- Crystal precipitation may exist in Wash Buffer (20X) at low temperatures. Please dissolve it completely using a water bath at room temperature prior to use.
- Standard solution should be prepared freshly. Discard the rest after use.
- Avoid oxidizer and metal contamination that cause the invalidation of TMB Solution.
- Change pipette tips between different samples and liquids to prevent contamination and incorrect loading volumes.
- Do not mix or interchange reagents from different kit lots.
- It is particularly important to perform sufficient mixing of reactions to ensure an accurate result. Please shake the 96-well plate gently after the addition of reagents.
- Most procedures of this experiment should be performed at room temperature (25-28ºC). Temperature lower than 25ºC will result in a significant decrease in the absorbance value of reactions.
- The washing process is very important. Insufficient wash will result in reduced accuracy and increased experimental errors.
- Run all standards, controls, and samples in duplicate.
- Avoid the formation of air bubbles when adding the sample.
- This product is for R&D only. Not for drug, household, or other uses.
- For your safety and health, please wear a lab coat and disposable gloves during the operation.

Instructions for Use:

1. **Preparation of the sample.**
    a. **Cell supernatant:** Centrifuge cell cultures at 100-500×g for 5 minutes to collect the supernatant.
    b. **Serum:** Leave the whole blood undisturbed at room temperature for 30 minutes to 2 hours. After the whole blood clots, collect the yellow supernatant (serum) by centrifuging the whole blood at 1000-2000×g for 10 minutes at 4ºC, and keep the serum on ice.

Note: Do Not add any preservatives or anticoagulants to serum.

c. **Plasma:** Add heparin or EDTA anticoagulant to whole blood and place on ice after mix. After centrifuging at 1000-2000×g for 10 minutes at 4ºC, collect the yellow supernatant (plasma) and keep it on ice.

***Note 1:** If the samples cannot be analyzed immediately, make aliquots and store them at -20ºC or -80ºC. Avoid repeated freeze-thaw.*

***Note 2:** Samples should be clear and transparent. Remove any particulates from samples by centrifuging before being analyzed.*

***Note 3:** Do not use hemolyzed, hyperlipidemia, or contaminated samples for analysis.*

***Note 4:** Serum or plasma samples may need to be properly diluted with Assay Buffer before the assay.*

2. **Preparation of the kit.**

a. Thaw the reagents and equilibrate to room temperature (25-28ºC) prior to use. Store the reagents at 4ºC immediately after use.

b. Prepare an appropriate amount of 1X Wash Buffer by diluting the Wash Buffer (20X) with ddH_2O.

c. Reconstitute Standard to 8000pg/ml with Standard Dilution Buffer according to the instructions labeled on the standard vial. Mix gently and incubate for 15min at room temperature. Gently pipette the contents several times to dissolve the standard completely. Generally, each concentration of standard is analyzed at least in duplicates (at least 2 wells) and 100μl is needed for each well. If one bottle of Standard is not enough, use more bottles of Standard, but the contents from different bottles need to be mixed before performing dilutions.

d. Add 250μl of Standard Dilution Buffer to each of 5 tubes and make serial dilutions as shown in Figure 2 to obtain 5 dilutions: 1/2 (4000pg/ml), 1/4 (2000pg/ml), 1/8 (1000pg/ml), 1/16 (500pg/ml), 1/32 (250pg/ml). Mix thoroughly between steps.

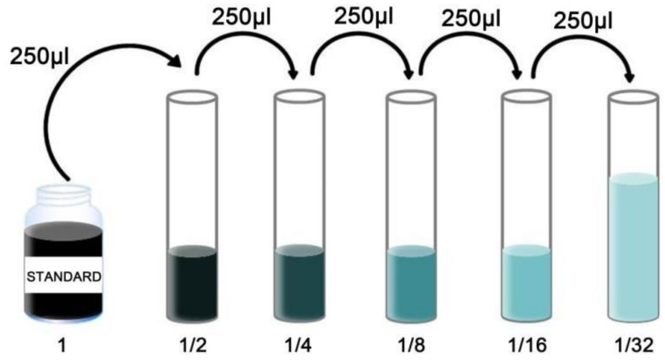

Figure 2. Dilution diagram for preparation of different concentrations of Standard.

3. **Perform ELISA.**

a. Determine the number of pre-coated 8-well strips required for the experiment. Insert the strips in a frame for use. Re-bag any unused strips and store them at 4ºC.

b. Prepare the Standard freshly and plot a standard curve for every run. Set up the blank control by only adding TMB Solution and Stop Solution.

c. Add 100μl of samples or standards to each well. Cover wells with Adhesive Films (transparent), and incubate for 2 hours at room temperature.

***Note:** For the assay of HGF in serum or plasma samples, add 50 μl of Assay Buffer followed by 50 μl of sample. At this point, the sample is diluted 2 times. If the sample concentration is too high and beyond the detection range, pleas add 50 μl of Assay Buffer at first, and then add 50 μl of diluted sample. For cell lysate samples, a preliminary test is required to determine the optimal dilution factor, and the assay method is the same as for serum and plasma samples. It is necessary to record the dilution factor of the sample.*

d. Thoroughly aspirate the solution by gently lowering a pipette tip into the bottom of each well and wash wells 5 times with 300μl 1X Wash Buffer. Allow the buffer to stand for 15-30 seconds before aspiration. After the last wash, invert the strip and tap dry on absorbent tissue.

e. Add 100μl of Biotin-conjugated Antibody to each well (***Note:** the antibody can be used directly without dilution*). Cover wells with Adhesive Films (transparent) and incubate for 1 hour at room temperature.

f. Thoroughly aspirate the solution and wash wells 5 times with 300μl of 1X Wash Buffer, as described in step 3d.

g. Add 100μl of HRP-labeled Streptavidin to each well (***Note:** the HRP-labeled Streptavidin can be used directly without dilution*), cover wells with Adhesive Films (white), and incubate at room temperature for 20 minutes in the dark. If the room temperature is

low, prolong the incubation time as appropriate.

h. Thoroughly aspirate the solution and wash wells 5 times with 300μl of 1X Wash Buffer, as described in step 3d.

i. Add 100μl of TMB Solution to each well, cover wells with Adhesive Films (white), and incubate at room temperature for 15-20 minutes in the dark. If the room temperature is low, prolong the incubation time until the standard sample exhibits significant color change. If the concentration of target protein in sample is high, color change will occur soon.

j. Add 50μl of Stop Solution to each well. Read the absorbance at 450nm immediately after mixing.

4. Analysis.

a. Calculate the average A450 value for each standard and sample. Duplicates should be within 20 percent of the mean value.

b. Subtract the A450 value of blank control from the A450 values of standards and samples (This step can be omitted if there is no blank control).

c. Generate the standard curve by plotting the concentrations of standard on the abscissa and their corresponding A450 values on the ordinate (Figure 3).

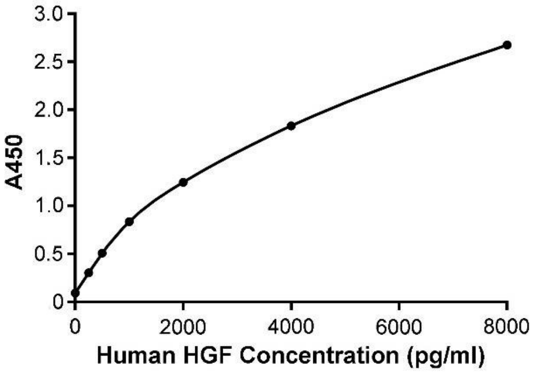

Figure 3. The standard curve of Beyotime's Human HGF ELISA Kit (PH385). This figure is for reference only, which may vary due to different experimental conditions.

d. Determine the concentrations of human HGF in samples from the standard curve based on their A450 values.

Note: *Dilute samples producing signals greater than the upper limit of the standard curve in Standard Dilution Buffer and reanalyze. Multiply the concentration by the dilution factor.*

Related Products:

Cat. No.	Product Name	Pack Size
PH385	Human HGF ELISA Kit	96T

Version 2018.11.21

MULTI SCIENCES
联科生物

Human IL-20 ELISA Kit

Catalog Number:
EK120 - 48
EK120 - 96

For the quantitative determination of human Interleukin 20 (IL-20) concentrations in cell culture supernates, serum and plasma.

This package insert must be read entirely before using this product. For proper performance, follow the protocol provided with each individual kit.

FOR RESEARCH USE ONLY. NOT FOR USE IN DIAGNOSTIC PROCEDURES.

MULTISCIENCES (LIANKE) BIOTECH CO., LTD.
3F, Building 2, No.1688 Tianma Road, Times High Tech Park,
Xiaoshan District, Hangzhou, Zhejiang Province, China.
www.multisciences.net
Tel: +86-057128874209
E-mail: info.cn@liankebio.com

TABLE OF CONTENTS

ASSAY PROCEDURE SUMMARY 1

Introduction

Description 2
Principle of the Assay 2
Limitations of the Procedure 2

General Information

Materials Provided 3
Storage 3
Other Supplies Required 4
Precaution 4
Technical Hints 5

Assay Protocol

Sample Collection and Storage 5
Reagent Preparation 6, 7
Assay Procedure 7, 8

Analysis

Calculation of Results 8
Typical Data 8
Sensitivity 9
Precision 9
Recovery 9
Linearity 9
Calibration 10
Sample Values 10
Specificity 10

ASSAY PROCEDURE SUMMARY

1. Prepare all reagents and standards as directed.

↓

2. Add 300 µl *Washing Buffer (1×)* per well to soak for about 30 seconds. Use immediately after aspirate.

↓

3. Add 100 µl 2-fold diluted Standard to Standard well. Add 100 µl *Standard Diluent/culture medium* to Blank well.

↓

4. Serum/Plasma: Add 50 µl *Assay Buffer (1×)* and 50 µl sample to the sample well.
Cell culture Supernates: Add 100 µl cell culture supernates to the sample well.

↓

5. Add 50 µl of diluted *Detect Antibody* to each well. Step 3, 4 and 5 should be completed within 15 minutes. Incubate for 2 hours at RT. Aspirate and wash 6 times.

↓

6. Add 100 µl of diluted *Streptavidin-HRP* to each well. Incubate for 45 minutes at RT. Aspirate and wash 6 times.

↓

7. Add 100 µl *Substrate Solution* to each well.
Incubate for 5 - 30 minutes at RT. Protect from light.

↓

8. Add 100 µl *Stop Solution* to each well.

↓

9. Read at 450 nm within 30 minutes. Correction 570 or 630 nm.

DESCRIPTION

Human interleukin 20 (IL-20) is a protein belonging to the IL-10 family of cytokines. It exhibits approximately 28% amino acid (aa) identity with IL-10 and 76% aa identity with mouse IL-20. IL-20 is produced by activated keratinocytes and monocytes and transmits an intracellular signal through two distinct cell-surface receptor complexes on keratinocytes and other epithelial cells. A specific receptor for this cytokine is found to be expressed in skin and upregulated dramatically in psoriatic skin, suggesting a role for this protein in epidermal function and psoriasis. In addition, IL-20 regulates proliferation and differentiation of keratinocytes during inflammation, particularly inflammation associated with the skin. IL-20 also causes cell expansion of multipotential hematopoietic progenitor cells. The over-expression of both human and mouse forms of IL-20 results in keratinocyte hyperproliferation, abnormal epidermal differentiation, and neonatal lethality.

PRINCIPLE OF THE ASSAY

Human IL-20 ELISA Kit is based on the quantitative sandwich enzyme-linked immunosorbent assay technique to measure concentration of human IL-20 in the samples. A monoclonal antibody specific for human IL-20 has been immobilized onto microwells. Standard or samples are pipetted into the wells, followed by the addition of biotin-linked detect antibody specific for IL-20, and IL-20 present is bound by the immobilized antibody and detect antibody following the first incubation. After removal of any unbound substances, streptavidin-HRP is added for a second incubation. After washing, substrate solution reacts with HRP and color develops in proportion to the amount of IL-20 bound by the immobilized antibody. The color development is stopped by addition of acid and the optical density value is measured by microplate reader.

LIMITATIONS OF THE PROCEDURE

- FOR RESEARCH USE ONLY. NOT FOR USE IN DIAGNOSTIC PROCEDURES.
- Do not use expired kit or reagents.
- Do not use reagents from other lots or manufacturers. Do not prepare component by yourself.
- If concentration of assayed factor in samples is higher than the highest standard, dilute the serum/plasma samples with *Assay Buffer*, dilute the cell culture supernate samples with *cell culture medium*. Reanalyze these and multiply results by the appropriate dilution factor.
- Any variation in testing personnel, sample preparation, standard dilution, pipetting technique, washing techniques, incubation time, temperature, kit age and equipment can cause variation in results.
- This assay is designed to eliminate interference by factors present in biological samples. Until all factors have been tested in the ELISA immunoassay, the possibility of interference cannot be excluded.

MATERIALS PROVIDED (96 Test)

Unopened kit should be stored at 2 - 8℃.

- **Human IL-20 Microplate** (1 plate): 96-well polystyrene microplate (12 strips of 8 wells) coated with a monoclonal antibody against human IL-20.
- **Human IL-20 Standard** (2 vials): Recombinant human IL-20 in a buffered protein base with preservatives; lyophilized.
- **Human IL-20 Detect Antibody** (1 vial): Biotin-conjugate anti-human IL-20 detect antibody; 100× liquid.
- **Standard Diluent** (1 bottle, 5 ml): In some, very rare cases, an insoluble precipitate of stabilizing protein has been seen in the Standard Diluent. This precipitate does not interfere in any way with the performance of the test and can thus be ignored.
- **Streptavidin-HRP** (1 vial): 100× liquid.
- **Assay Buffer (10×)** (1 bottle, 5 ml): PBS with 0.5 % Tween-20 and 5 % BSA.
- **Substrate** (1 bottle, 11 ml): TMB (tetramethyl-benzidine).
- **Stop Solution** (1 bottle, 11 ml): 0.18 M sulfuric acid.
- **Washing Buffer (20×)** (1 bottle, 50 ml): PBS with 1 % Tween-20.
- **Plate Covers** (6 strips).

STORAGE

Store kit reagents between 2 and 8 ℃. Immediately after use remaining reagents should be returned to cold storage (2 to 8℃). Expiry of the kit and reagents is stated on labels.

Expiration date of the kit components can only be guaranteed if the components are stored properly, and if, in case of repeated use of one component, this reagent is not contaminated by the first handling.

<table>
<tr><th colspan="2">Unopened kit</th><td>Store at 2 - 8℃ (See expiration date on the label).</td></tr>
<tr><th rowspan="3">Opened/
Reconstituted
Reagents</th><td>1× Washing Buffer
1× Assay Buffer
Stop Solution
Standard Diluent
Substrate TMB
Detect Antibody
Streptavidin-HRP</td><td>Up to 1 month at 2 - 8℃.</td></tr>
<tr><td>Standard</td><td>Up to 1 month at ≤ -20 ℃ in a manual defrost freezer. Discard after use.</td></tr>
<tr><td>Microplate Wells</td><td>Up to 1 month at 2 - 8℃. Return unused strips to the foil pouch containing the desiccant pack, reseal along entire edge to maintain plate integrity.</td></tr>
</table>

Provided this is within the expiration date of the kit.

OTHER SUPPLIES REQUIRED

- **Microplate reader** capable of measuring absorbance at 450 nm, with correction wavelength set at 570 nm or 630 nm.
- **Pipettes and pipette tips**.
- 50 µl to 300 µl adjustable **multichannel micropipette** with disposable tips.
- Multichannel micropipette **reservoir**.
- **Beakers, flasks, cylinders** necessary for preparation of reagents.
- **Deionized or distilled water**.
- **Polypropylene** test tubes for dilution.

PRECAUTION

- All chemicals should be considered as potentially hazardous.
- We therefore recommend that this product is handled only by those persons who have been trained in laboratory techniques and that it is used in accordance with the principles of good laboratory practice. Wear suitable protective clothing such as laboratory overalls, safety glasses and gloves.
- Care should be taken to avoid contact with skin or eyes. In the case of contact with skin or eyes wash immediately with water. See material safety data sheet(s) and/or safety statement(s) for specific advice.
- The Stop Solution provided with this kit is an acid solution. Wear eyes, hand, face, and clothing protection when using this material.
- Reagents are intended for research use only and are not for use in diagnostic or therapeutic procedures.
- Do not mix or substitute reagents with those from other lots or other sources.
- Do not use kit reagents beyond expiration date on label.
- Do not expose kit reagents to strong light during storage and incubation.
- Do not eat or smoke in areas where kit reagents or samples are handled.
- Avoid contact of skin or mucous membranes with kit reagents or specimens.
- Rubber or disposable latex gloves should be worn while handling kit reagents or specimens.
- Avoid contact of substrate solution with oxidizing agents and metal.
- Avoid splashing or generation of aerosols.
- In order to avoid microbial contamination or cross- contamination of reagents or specimens which may invalidate the test use disposable pipette tips and/or pipettes.
- Use clean, dedicated reagent trays for dispensing the conjugate and substrate reagent.
- Exposure to acid inactivates the HRP and antibody conjugate.
- Glass-distilled water or deionized water must be used for reagent preparation.
- Substrate solution must be warmed to room temperature prior to use.
- Decontaminate and dispose specimens and all potentially contaminated materials as they could contain infectious agents. The preferred method of decontamination is autoclaving for a minimum of 1 hour at 121.5℃.
- Liquid wastes not containing acid and neutralized waste may be mixed with sodium hypochlorite in volumes such that the final mixture contains 1.0 % sodium hypochlorite. Allow 30 minutes for effective decontamination. Liquid waste containing acid must be neutralized prior to the addition of sodium hypochlorite.
- In some cases, an insoluble precipitate of stabilizing protein has been seen in the Standard Diluent. This precipitate does not interfere in any way with the performance of the test and can thus be ignored. Or remove precipitate by centrifuging at 6,000 × g for 5 minutes.

TECHNICAL HINTS

- When mixing or reconstituting protein solutions, always avoid foaming.
- To avoid cross-contamination, change pipette tips between additions of each standard level, between sample additions, and between reagent additions. Also, use separate reservoirs for each reagent.
- When using an automated plate washer, adding a 30 seconds soak period before washing step and/or rotating the plate between wash steps may improve assay precision.
- To ensure accurate results, proper adhesion of plate sealers during incubation steps is necessary.
- Substrate Solution should remain colorless until added to the plate. Keep Substrate Solution protected from light. Substrate Solution should change from colorless to gradations of blue.
- Stop Solution should be added to the plate in the same order as the Substrate Solution.
- The color developed in the wells will turn from blue to yellow upon addition of the Stop Solution. Wells that are green in color indicate that the Stop Solution has not mixed thoroughly with the Substrate Solution.
- It is recommended that all samples and standards be assayed in duplicate.
- Take care not to scratch the inner surface of the microwells.

SAMPLE COLLECTION AND STORAGE

Cell Culture Supernates – Remove particulates by centrifugation at 300 × g for 10 minutes and assay immediately or aliquot and store samples at ≤ -20℃.

Serum – Use a serum separator tube (SST) and allow samples to clot for 30 minutes before centrifugation for 10 minutes at 1,000 × g. Remove serum and assay immediately or aliquot and store samples at ≤ -20℃.

Plasma – Collect plasma using EDTA, citrate or heparin as anticoagulant. Centrifuge at 1,000 × g within 30 minutes of collection. Assay immediately or aliquot and store samples at ≤ -20℃.

Other biological samples might be suitable for use in the assay. Cell culture supernates, serum and plasma were tested with this assay.

Note: Samples containing a visible precipitate must be clarified prior to use in the assay. Do not use grossly hemolyzed or lipemic specimens.
If samples are to be run within 24 hours, they may be stored at 2 to 8 ℃. For longer storage, aliquot samples and store frozen at -20℃ to avoid loss of bioactive human IL-20. Avoid repeated freeze-thaw cycles.

REAGENT PREPARATION

Bring all reagents and samples to room temperature before use.
If crystals form in the Buffer Concentrates, warm and gently stir them until completely dissolved.

Washing Buffer (1×)

Pour entire contents (50 ml) of the **Washing Buffer (20×)** into a clean 1,000 ml graduated cylinder. Bring to final volume of 1,000 ml with pure or deionized water.
Mix gently to avoid foaming.
Transfer to a clean wash bottle and store at 2 to 25℃. Washing Buffer (1×) is stable for 30 days.

Assay Buffer (1×)

Pour the entire contents (5 ml) of the **Assay Buffer (10×)** into a clean 100 ml graduated cylinder. Bring to final volume of 50 ml with distilled water. Mix gently to avoid foaming.
Store at 2 to 8℃. Assay Buffer (1×) is stable for 30 days.

Detect Antibody

Mix well prior to making dilutions.
Make a **1：100** dilution of the concentrated **Detect Antibody** solution with Assay Buffer (1×) in a clean plastic tube as needed.
The diluted Detect Antibody should be used within 30 minutes after dilution.

Streptavidin-HRP

Mix well prior to making dilutions.
Make a **1：100** dilution of the concentrated **Streptavidin-HRP** solution with Assay Buffer (1×) in a clean plastic tube as needed.
The diluted Streptavidin-HRP should be used within 30 minutes after dilution.

Sample Dilution

If your samples have high IL-20 content, dilute serum/plasma samples with Assay Buffer (1×). For cell culture supernates, dilute with cell culture medium.

Human IL-20 Standard

Reconstitute **Human IL-20 Standard** by addition of distilled water. Reconstitution volume is stated on the label of the standard vial. Swirl or mix gently to insure complete and homogeneous solubilization (concentration of reconstituted standard = 6,000 pg/ml).
Allow the standard to reconstitute for 10 - 30 minutes. Mix well prior to making dilutions.
Use polypropylene tubes.

For serum/plasma samples, mixing *concentrated human IL-20 standard* (230 µl) with 230 µl of *Standard Diluent* creates the high standard (3,000 pg/ml). Pipette 230 µl of *Standard Diluent* into each tube. Use the high standard to produce a 1:1 dilution series (scheme below). Mix each tube thoroughly before the next transfer. *Standard Diluent* serves as the zero standard (0 pg/ml).

For cell culture supernates, mixing *concentrated human IL-20 standard* (230 µl) with 230 µl of cell culture medium creates the high standard (3,000 pg/ml). Pipette 230 µl of cell culture medium into each tube. Use the high standard to produce a 1:1 dilution series. Mix each tube thoroughly before the next transfer. Cell culture medium serves as the zero standard (0 pg/ml).

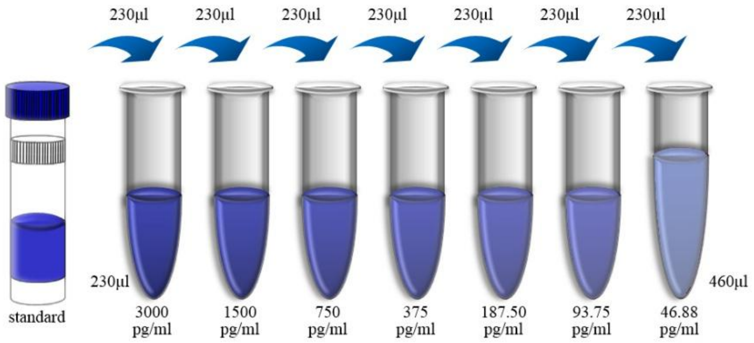

ASSAY PROCEDURE

Bring all reagents and samples to room temperature before use.

1. Prepare all reagents including microplate, samples, standards and working solution as described in the previous sections.
2. Remove excess microplate strips and return them to the foil pouch containing the desiccant pack, and reseal for further use.
3. Add 300 µl *Washing Buffer (1×)* per well, and allow it for about 30 seconds before aspiration. Soaking is highly recommended to obtain a good test performance. Empty wells and tap microwell strips on absorbent pad or paper towel to remove excess *Washing Buffer (1×)*. Use the microwell strips immediately after washing. **Do not allow wells to dry.**
4. Add 100 µl 2-fold diluted Standard to Standard well. Add 100 µl *Standard Diluent / culture medium* to Blank well.
5. Serum/Plasma: Add 50 µl *Assay Buffer (1×)* and 50 µl sample to the sample well. Cell culture supernates: Add 100 µl cell culture supernates to the sample well.
6. Add 50 µl of diluted *Detect Antibody* to each well. Ensure reagent addition in step 4, 5 and 6 is uninterrupted and completed within 15 minutes.
7. Seal the plate with an *adhesive film*. Incubate at room temperature (18 to 25℃) for 2 hours on a microplate shaker set at 300 rpm.
8. Aspirate each well and wash by filling each well with 300 µl *Washing Buffer (1×)*, repeat five times for a total six washes. Complete removal of liquid at each step is essential to the best performance. After the last wash, remove any remaining *Washing Buffer (1×)* by aspirating or decanting. Invert the plate and tap it against clean paper towels .
9. Add 100 µl of diluted *Streptavidin-HRP* to each well.

10. Seal the plate with a fresh *adhesive film*. Incubate at room temperature (18 to 25 ℃) for 45 minutes on a microplate shaker set at 300 rpm.
11. Repeat aspiration/wash as in step 8.
12. Add 100 µl of *Substrate Solution* to each well. Incubate for 5 - 30 minutes at room temperature. Protect from light.
13. Add 100 µl of *Stop Solution* to each well. The color will turn yellow. If the color in the well is green or if the color change does not appear uniform, gently tap the plate to ensure thorough mixing.
14. Measure the optical density value within 30 minutes by microplate reader set to 450 nm. If wavelength correction is available, set to 570 nm or 630 nm. If wavelength correction is not available, subtract readings at 570 nm or 630 nm from the readings at 450 nm. This subtraction will correct for optical imperfections in the plate. Reading directly at 450 nm without correction may generate higher concentration than true value.

CALCULATION OF RESULTS

Average the duplicate optical density readings for each standards and sample, then subtract the average optical density value of the zero standard.

Standard Concentration as horizontal axis, optical density (OD) Value as the vertical axis, regressing the data and create a standard curve using computer software. The data may be linearized by plotting the log of the IL-20 concentrations versus the log of the OD and the best fit line can be determined by regression analysis. This procedure will produce an adequate but less precise fit of the data.

Note: The finally concentration of top standard is 3,000 pg/ml.
If samples have been diluted following the instruction, the final dilution factor is 2. If sample have been diluted by other means, the concentration read from the standard curve must be multiplied by the appropriate dilution factor.

TYPICAL DATA

A standard curve must be run within each assay. This standard curve is provided for demonstration only.

pg/ml	O.D.		Average	Corrected
0.00	0.029	0.033	0.031	
46.88	0.049	0.052	0.051	0.020
93.75	0.073	0.075	0.074	0.043
187.50	0.122	0.119	0.121	0.090
375.00	0.253	0.247	0.250	0.219
750.00	0.548	0.557	0.553	0.522
1500.00	1.198	1.196	1.197	1.166
3000.00	2.325	2.399	2.362	2.331

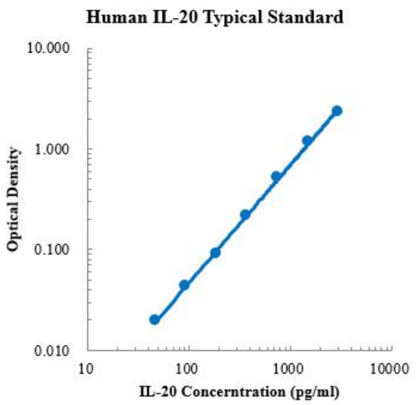

SENSITIVITY

The minimum detectable dose (MDD) of IL-20 is typically about 11.87 pg/ml (mean of 6 independent assays).
The MDD was determined by adding two standard deviations to the mean optical density value of ten zero standard replicates and calculating the corresponding concentration.

PRECISION

Intra-assay Precision (Precision within an assay)
Three serum-based and buffer-based samples of known concentration were tested twenty times on one plate to assess intra-assay precision.

Inter-assay Precision (Precision between assays)
Three serum-based and buffer-based samples of known concentration were tested in six separate assays to assess inter-assay precision.

	Intra-assay precision				Inter-assay precision		
**Sample**	1	2	3		1	2	3
**n**	20	20	20		6	6	6
**Mean (pg/ml)**	162.8	507.9	1635.9		219.8	616.2	1808.0
**Standard deviation**	6.9	16.8	48.0		10.3	28.9	49.7
**CV (%)**	4.2	3.3	2.9		4.7	4.7	2.8

RECOVERY

The spike recovery was evaluated by spiking 3 levels of human IL-20 into five health human serum samples. The un-spiked serum was used as blank in these experiments.
The recovery ranged from 78 % to 121 % with an overall mean recovery of 96 %.

LINEARITY

To assess the linearity of the assay, five samples were spiked with high concentration of IL-20 in human serum and diluted with Standard Diluent to produce samples with values within the

dynamic range of the assay.

	Average (%)	Range (%)
**1:2**	109	108 - 116
**1:4**	112	111 - 119
**1:8**	116	113 - 121
**1:16**	113	107 - 119

CALIBRATION

This immunoassay is calibrated against a highly purified recombinant human IL-20 produced at MultiSciences.

SAMPLE VALUES

Serum/Plasma - Thirty samples from apparently healthy volunteers were evaluated for the presence of IL-20 in this assay. No medical histories were available for the donors used in this study.

Sample Matrix	Number of Samples Evaluated	Range (pg/ml)	Detectable (%)	Mean of Detectable (pg/ml)
Serum	30	145.9 - 555.8	100	271.9

Note: The sample range is non-physiological range. The sample range of healthy human will difference according to species, sample preparation, and testing personnel, equipment varies. The above information is only reference.

SPECIFICITY

This kit could assay both natural and recombinant human IL-20. A panel of substances listed below were prepared at 1 ng/ml in Standard Diluent to determine cross-reactivity. Preparations of the following substances at 1 ng/ml in a mid-range rhIL-20 control to determine interference. No significant cross-reactivity or interference was observed.

Human		Mouse	Rat
IFN-γ	IL-17A	GM-CSF	IFN-γ
IL-1β	IL-18	IFN-γ	IL-1β
IL-2	IL-21	IL-1β	IL-4
IL-4	IL-22	IL-2	IL-6
IL-5	IL-23	IL-4	IL-10
IL-6	MCP-1	IL-6	TNF-α
IL-8	TGF-β1	IL-10	

IL-10	TNF-α	IL-17A	
IL-12	VEGF	TNF-α	

PLATE LAYOUT

	1	2	3	4	5	6	7	8	9	10	11	12
A	S1	S1										
B	S2	S2										
C	S3	S3										
D	S4	S4										
E	S5	S5										
F	S6	S6										
G	S7	S7										
H	Blank	Blank										

MULTI SCIENCES
联科生物

Human TWEAK ELISA Kit

Catalog Number: EK1256
Size: 48 Test, 96 Test, 2 × 96 Test, 5 × 96 Test, 10 × 96 Test

For the quantitative determination of human Tumor necrosis factor-like weak inducer of apoptosis (TWEAK) concentrations in cell culture supernates, serum and plasma.

This package insert must be read entirely before using this product. For proper performance, follow the protocol provided with each individual kit.

FOR RESEARCH USE ONLY. NOT FOR USE IN DIAGNOSTIC PROCEDURES.

MULTISCIENCES (LIANKE) BIOTECH, CO., LTD.
13F, Building 3, 3rd Phase, 108 Xiang Yuan Road, Gongshu Intellect Information Industry Park, Hangzhou, Zhejiang Province, China.
www.multisciences.net
Tel: +86 057128828618-88662
Fax: +86-0571-28828618
E-mail: info.cn@liankebio.com

TABLE OF CONTENTS

ASSAY PRECEDURE SUMMARY 1

Introduction

Description 2
Principle of the Assay 2
Limitations of the Procedure 2

General Information

Materials Provided 3
Storage 3
Other Supplies Required 4
Precaution 4
Technical Hints 5

Assay Protocol

Sample Collection and Storage 5
Reagent Preparation 6, 7
Assay Procedure 7, 8

Analysis

Calculation of Results 8
Typical Data 8
Sensitivity 9
Precision 9
Recovery 9
Linearity 9
Calibration 10
Sample Values 10
Specificity 10

ASSAY PROCEDURE SUMMARY

1. Prepare all reagents and standards as directed.

2. Add 100 µl 2-fold diluted *Standard* to Standard well in duplicate. Add 100 µl *Standard Diluent* to Blank well in duplicate.

3. Add 50 µl *Assay Buffer (1×)* and 50 µl sample to the sample well

4. Add 50 µl diluted *Detect Antibody* to each well. Step 2, 3 and 4 should be completed within 15 minutes.

5. Incubate for 2 hours at RT.

6. Aspirate and wash 6 times.

7. Add 100 µl *Streptavidin-HRP* to each well.

8. Incubate for 45 minutes at RT.

9. Aspirate and wash 6 times.

10. Add 100 µl *Substrate Solution* to each well. Incubate for 5 - 30 minutes at RT. Protect from light.

11. Add 100 µl *Stop Solution* to each well.

12. Read at 450 nm within 30 minutes. Correction 570 or 630 nm.

DESCRIPTION

Tumor necrosis factor-like weak inducer of apoptosis (TWEAK) was discovered in 1997. The protein encoded by this gene is a cytokine that belongs to the tumor necrosis factor (TNF) ligand family. Leukocytes are the main source of TWEAK including human resting and activated monocytes, dendritic cells and natural killer cells. TWEAK can induce apoptosis via multiple pathways of cell death in a cell type-specific manner. This cytokine is also found to promote proliferation and migration of endothelial cells, and thus acts as a regulator of angiogenesis. Excessive activation of the TWEAK pathway in chronic injury has been described to promote pathological tissue changes including chronic inflammation, fibrosis and angiogenesis.

PRINCIPLE OF THE ASSAY

This assay employs the quantitative sandwich enzyme immunoassay technique. A monoclonal antibody specific for human TWEAK has been pre-coated onto a microplate. Standard, samples and biotin-linked detect antibody specific for TWEAK are pipetted into the wells and TWEAK present is bound by the immobilized antibody and detect antibody following incubation. After washing away any unbound substances, streptavidin-HRP is added. After washing, substrate solution is added to the wells and color develops in proportion to the amount of TWEAK bound in the initial step. The color development is stopped and the intensity of the color is measured.

LIMITATIONS OF THE PROCEDURE

- FOR RESEARCH USE ONLY. NOT FOR USE IN DIAGNOSTIC PROCEDURES.
- Do not use expired kit or reagents.
- Do not use reagents from other lots or manufacturers. Do not prepare component by yourself.
- If concentration of assayed factor in samples is higher than the highest standard, dilute the serum/plasma samples with *Assay Buffer*, dilute the cell culture supernate samples with *cell culture medium*. Reanalyze these and multiply results by the appropriate dilution factor.
- Any variation in testing personnel, sample preparation, standard dilution, pipetting technique, washing techniques, incubation time, temperature, kit age and equipment can cause variation in results.
- This assay is designed to eliminate interference by factors present in biological samples. Until all factors have been tested in the ELISA immunoassay, the possibility of interference cannot be excluded.

MATERIALS PROVIDED (96 Test)

Unopened kit should be stored at 2 - 8℃.

- **TWEAK Microplate** (1 plate): 96-well polystyrene microplate (12 strips of 8 wells) coated with a monoclonal antibody against human TWEAK.
- **TWEAK Standard** (2 vials): Recombinant human TWEAK in a buffered protein base with preservatives; lyophilized.
- **TWEAK Detect Antibody** (1 vial, 80 μl): Biotin-conjugate anti-human TWEAK detect antibody; 100× liquid.
- **Standard Diluent** (1 bottle, 5 ml): In some, very rare cases, an insoluble precipitate of stabilizing protein has been seen in the Standard Diluent vial. This precipitate does not interfere in any way with the performance of the test and can thus be ignored.
- **Streptavidin-HRP** (1 vial, 150 μl): 100× liquid.
- **Assay Buffer (10×)** (1 bottle, 5 ml): PBS with 0.5 % Tween-20 and 5 % BSA.
- **Substrate** (1 bottle, 15 ml): TMB (tetramethyl-benzidine).
- **Stop Solution** (1 bottle, 15 ml): 0.18 M sulfuric acid.
- **Washing Buffer (20×)** (1 bottle, 50 ml): PBS with 1 % Tween-20.
- **Plate Covers** (5 strips).

STORAGE

Store kit reagents between 2 and 8 ℃. Immediately after use remaining reagents should be returned to cold storage (2 to 8℃). Expiry of the kit and reagents is stated on labels.

Expiration date of the kit components can only be guaranteed if the components are stored properly, and if, in case of repeated use of one component, this reagent is not contaminated by the first handling.

**Unopened kit**		Store at 2 - 8℃ (See expiration date on the label).
**Opened/ Reconstituted Reagents**	1× Washing Buffer<br>1× Assay Buffer<br>Stop Solution<br>Standard Diluent<br>Substrate TMB<br>Detect Antibody<br>Streptavidin-HRP	Up to 1 month at 2 - 8℃.
	Standard	Up to 1 month at ≤ -20 ℃ in a manual defrost freezer. Discard after use.
	Microplate Wells	Up to 1 month at 2 - 8℃. Return unused strips to the foil pouch containing the desiccant pack, reseal along entire edge to maintain plate integrity.

Provided this is within the expiration date of the kit.

OTHER SUPPLIES REQUIRED

- **Microplate reader** capable of measuring absorbance at 450 nm, with correction wavelength set at 570 nm or 630 nm.
- **Pipettes and pipette tips**.
- 50 µl to 300 µl adjustable **multichannel micropipette** with disposable tips.
- Multichannel micropipette **reservoir**.
- **Beakers, flasks, cylinders** necessary for preparation of reagents.
- **Deionized or distilled water**.
- **Polypropylene** test tubes for dilution.

PRECAUTION

- All chemicals should be considered as potentially hazardous.
- We therefore recommend that this product is handled only by those persons who have been trained in laboratory techniques and that it is used in accordance with the principles of good laboratory practice. Wear suitable protective clothing such as laboratory overalls, safety glasses and gloves.
- Care should be taken to avoid contact with skin or eyes. In the case of contact with skin or eyes wash immediately with water. See material safety data sheet(s) and/or safety statement(s) for specific advice.
- The Stop Solution provided with this kit is an acid solution. Wear eyes, hand, face, and clothing protection when using this material.
- Reagents are intended for research use only and are not for use in diagnostic or therapeutic procedures.
- Do not mix or substitute reagents with those from other lots or other sources.
- Do not use kit reagents beyond expiration date on label.
- Do not expose kit reagents to strong light during storage and incubation.
- Do not eat or smoke in areas where kit reagents or samples are handled.
- Avoid contact of skin or mucous membranes with kit reagents or specimens.
- Rubber or disposable latex gloves should be worn while handling kit reagents or specimens.
- Avoid contact of substrate solution with oxidizing agents and metal.
- Avoid splashing or generation of aerosols.
- In order to avoid microbial contamination or cross- contamination of reagents or specimens which may invalidate the test use disposable pipette tips and/or pipettes.
- Use clean, dedicated reagent trays for dispensing the conjugate and substrate reagent.
- Exposure to acid inactivates the HRP and antibody conjugate.
- Glass-distilled water or deionized water must be used for reagent preparation.
- Substrate solution must be warmed to room temperature prior to use.
- Decontaminate and dispose specimens and all potentially contaminated materials as they could contain infectious agents. The preferred method of decontamination is autoclaving for a minimum of 1 hour at 121.5℃.
- Liquid wastes not containing acid and neutralized waste may be mixed with sodium hypochlorite in volumes such that the final mixture contains 1.0 % sodium hypochlorite. Allow 30 minutes for effective decontamination. Liquid waste containing acid must be neutralized prior to the addition of sodium hypochlorite.
- In some cases, an insoluble precipitate of stabilizing protein has been seen in the Standard Diluent. This precipitate does not interfere in any way with the performance of the test and can thus be ignored. Or remove precipitate by centrifuging at 6,000 × g for 5 minutes.

TECHNICAL HINTS

- When mixing or reconstituting protein solutions, always avoid foaming.
- To avoid cross-contamination, change pipette tips between additions of each standard level, between sample additions, and between reagent additions. Also, use separate reservoirs for each reagent.
- When using an automated plate washer, adding a 30 seconds soak period before washing step and/or rotating the plate between wash steps may improve assay precision.
- To ensure accurate results, proper adhesion of plate sealers during incubation steps is necessary.
- Substrate Solution should remain colorless until added to the plate. Keep Substrate Solution protected from light. Substrate Solution should change from colorless to gradations of blue.
- Stop Solution should be added to the plate in the same order as the Substrate Solution.
- The color developed in the wells will turn from blue to yellow upon addition of the Stop Solution. Wells that are green in color indicate that the Stop Solution has not mixed thoroughly with the Substrate Solution.
- It is recommended that all samples and standards be assayed in duplicate.
- Take care not to scratch the inner surface of the microwells.

SAMPLE COLLECTION AND STORAGE

Cell Culture Supernates – Remove particulates by centrifugation at 300 × g for 10 minutes and assay immediately or aliquot and store samples at ≤ -20℃.

Serum – Use a serum separator tube (SST) and allow samples to clot for 30 minutes before centrifugation for 10 minutes at 1,000 × g. Remove serum and assay immediately or aliquot and store samples at ≤ -20℃.

Plasma – Collect plasma using EDTA, citrate or heparin as anticoagulant. Centrifuge at 1,000 × g within 30 minutes of collection. Assay immediately or aliquot and store samples at ≤ -20℃.

Other biological samples might be suitable for use in the assay. Cell culture supernates, serum and plasma were tested with this assay.

Note: Samples containing a visible precipitate must be clarified prior to use in the assay. Do not use grossly hemolyzed or lipemic specimens.
Samples should be aliquoted and must be stored frozen at -20℃ to avoid loss of bioactive human TWEAK. If samples are to be run within 24 hours, they may be stored at 2 to 8℃.
Avoid repeated freeze-thaw cycles. Prior to assay, the frozen sample should be brought to room temperature slowly and mixed gently.

REAGENT PREPARATION

Bring all reagents and samples to room temperature before use.
If crystals form in the Buffer Concentrates, warm and gently stir them until completely dissolved.

Washing Buffer (1×)

Pour entire contents (50 ml) of the **Washing Buffer (20×)** into a clean 1000 ml graduated cylinder.
Bring to final volume of 1000 ml with pure or deionized water.
Mix gently to avoid foaming.
Transfer to a clean wash bottle and store at 2 to 25℃. Washing Buffer (1×) is stable for 30 days.

Assay Buffer (1×)

Pour the entire contents (5 ml) of the **Assay Buffer (10×)** into a clean 100 ml graduated cylinder.
Bring to final volume of 50 ml with distilled water. Mix gently to avoid foaming.
Store at 2 to 8℃. Assay Buffer (1×) is stable for 30 days.

Detect Antibody

Mix well prior to making dilutions.
Make a **1：100** dilution of the concentrated **Detect Antibody** solution with Assay Buffer (1×) in a clean plastic tube as needed.
The diluted Detect Antibody should be used within 30 minutes after dilution.

Streptavidin-HRP

Mix well prior to making dilutions.
Make a **1：100** dilution of the concentrated **Streptavidin-HRP** solution with Assay Buffer (1×) in a clean plastic tube as needed.
The diluted Streptavidin-HRP should be used within 30 minutes after dilution.

Sample Dilution

If your samples have high TWEAK content, dilute serum/plasma samples with Assay Buffer (1×).
For cell culture supernates, dilute with cell culture medium.

Human TWEAK Standard

Reconstitute **Human TWEAK Standard** by addition of distilled water. Reconstitution volume is stated on the label of the standard vial. Swirl or mix gently to insure complete and homogeneous solubilization (concentration of reconstituted standard = 2,000 pg/ml).
Allow the standard to reconstitute for 10 - 30 minutes. Mix well prior to making dilutions.
Use polypropylene tubes.

For serum/plasma samples, mixing *concentrated human TWEAK standard* (250 µl) with 250 µl of *Standard Diluent* creates the high standard (1,000 pg/ml). Pipette 250 µl of *Standard Diluent* into each tube. Use the high standard to produce a 1:1 dilution series (scheme below). Mix each tube thoroughly before the next transfer. *Standard Diluent* serves as the zero standard (0 pg/ml).

For cell culture supernates, mixing *concentrated human TWEAK standard* (250 µl) with 250 µl of cell culture medium creates the high standard (1,000 pg/ml). Pipette 250 µl of cell culture medium into each tube. Use the high standard to produce a 1:1 dilution series. Mix each tube thoroughly before the next transfer. Cell culture medium serves as the zero standard (0 pg/ml).

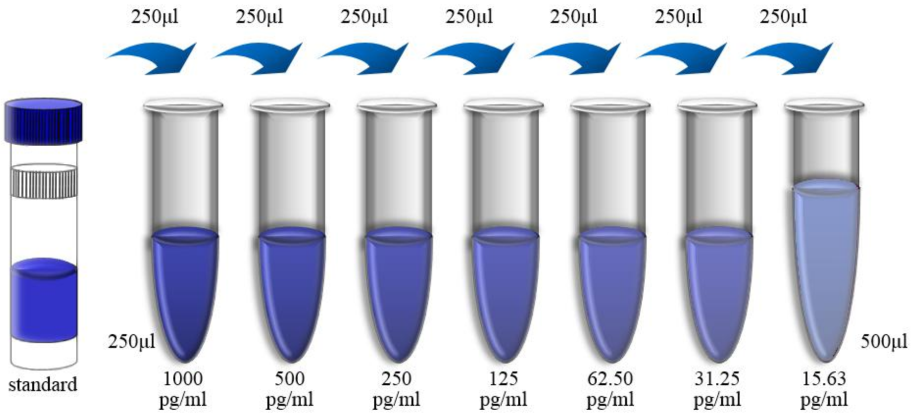

ASSAY PROCEDURE

Bring all reagents and samples to room temperature before use.

1. Prepare all reagents including microplate, samples, standards and working solution as described in the previous sections.
2. Remove excess microplate strips and return them to the foil pouch containing the desiccant pack, and reseal for further use.
3. Add 300 µl *Washing Buffer (1×)* per well, and allow it for about 30 seconds before aspiration. Soaking is highly recommended to obtain a good test performance. Empty wells and tap microwell strips on absorbent pad or paper towel to remove excess *Washing Buffer (1×)*. Use the microwell strips immediately after washing. **Do not allow wells to dry.**
4. Add 100 µl of 2-fold diluted *Standard* to Standard well in duplicate. Add 100 µl of *Standard Diluent* to Blank well in duplicate.
5. Add 50 µl of *Assay Buffer (1×)* and 50 µl sample to the sample well
6. Add 50 µl of diluted *Detect Antibody* to each well. Ensure reagent addition in step 4, 5 and 6 is uninterrupted and completed within 15 minutes.
7. Cover with an adhesive strip. Incubate at room temperature (18 to 25℃) for 2 hours on a microplate shaker set at 300 rpm.
8. Aspirate each well and wash, repeating the process five times for a total six washes. Wash by filling each well with 300µl *Washing Buffer (1×)*. Complete removal of liquid at each step is essential to good performance. After the last wash, remove any remaining Wash Buffer by aspirating or decanting. Invert the plate and blot it against clean paper towels.
9. Add 100 µl of diluted *Streptavidin-HRP* to each well.
10. Cover with a new adhesive strip. Incubate at room temperature (18 to 25℃) for 45 minutes on a microplate shaker set at 300 rpm.

11. Repeat aspiration/wash as in step 8.
12. Add 100 µl of *Substrate Solution* to each well. Incubate for 5 - 30 minutes at room temperature. Protect from light.
13. Add 100 µl of *Stop Solution* to each well. The color will turn yellow. If the color in the well is green or if the color change does not appear uniform, gently tap the plate to ensure thorough mixing.
14. Measure the optical density value within 30 minutes by microplate reader set to 450 nm. If wavelength correction is available, set to 570 nm or 630 nm. If wavelength correction is not available, subtract readings at 570 nm or 630 nm from the readings at 450 nm. This subtraction will correct for optical imperfections in the plate. Reading directly at 450 nm without correction may generate higher concentration than true value.

CALCULATION OF RESULTS

Average the duplicate optical density readings for each standards and sample, then subtract the average optical density value of the zero standard.

Standard Concentration as horizontal axis, optical density (OD) Value as the vertical axis, regressing the data and create a standard curve using computer software. The data may be linearized by plotting the log of the TWEAK concentrations versus the log of the OD and the best fit line can be determined by regression analysis. This procedure will produce an adequate but less precise fit of the data.

Note: The finally concentration of top standard is 1,000 pg/ml. If instruction in this protocol have been followed samples have been diluted by 1:1 ratio (50 µl sample + 50 µl Assay Buffer), the concentration read from the standard curve must be multiplied by the dilution factor (×2).
If samples have been diluted following the instruction, the final dilution factor is 2. If sample have been diluted by other means, the concentration read from the standard curve must be multiplied by the appropriate dilution factor.

TYPICAL DATA

A standard curve must be run within each assay. This standard curve is provided for demonstration only.

pg/ml	O.D.		Average	Corrected
0.00	0.040	0.042	0.041	
15.63	0.087	0.086	0.087	0.046
31.25	0.133	0.134	0.134	0.093
62.50	0.225	0.226	0.226	0.185
125.00	0.385	0.403	0.394	0.353
250.00	0.658	0.702	0.680	0.639
500.00	0.990	1.031	1.011	0.970
1000.00	1.759	1.770	1.765	1.724

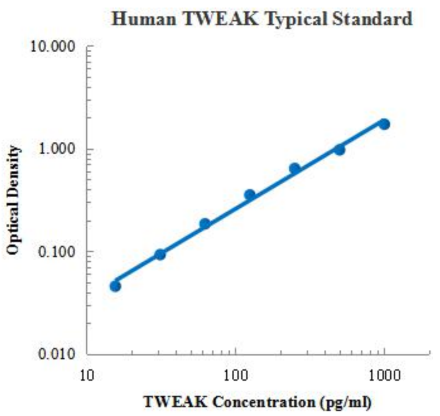

SENSITIVITY

The minimum detectable dose (MDD) of TWEAK is typically less than 0.87 pg/ml.
The MDD was determined by adding two standard deviations to the mean optical density value of ten zero standard replicates and calculating the corresponding concentration.

PRECISION

Intra-assay Precision (Precision within an assay)
Three serum-based and buffer-based samples of known concentration were tested twenty times on one plate to assess intra-assay precision.

Inter-assay Precision (Precision between assays)
Three serum-based and buffer-based samples of known concentration were tested in six separate assays to assess inter-assay precision.

	Intra-assay precision				Inter-assay precision		
**Sample**	1	2	3		1	2	3
**n**	20	20	20		6	6	6
**Mean (pg/ml)**	37.1	150.4	569.0		35.4	141.6	537.1
**Standard deviation**	1.6	5.6	37.5		2.3	7.3	25.0
**CV (%)**	4.3	3.7	6.6		6.5	5.2	4.7

RECOVERY

The spike recovery was evaluated by spiking 3 levels of humant TWEAK into five health human serum samples. The un-spiked serum was used as blank in these experiments.
The recovery ranged from 88% to 117 % with an overall mean recovery of 105 %.

LINEARITY

To assess the linearity of the assay, five samples were spiked with high concentration of TWEAK in human serum and diluted with Standard Diluent to produce samples with values within the dynamic range of the assay.

	Average (%)	Range (%)
**1:2**	91	85 - 95
**1:4**	99	95 - 105
**1:8**	105	98 - 114
**1:16**	103	95 - 110

CALIBRATION

This immunoassay is calibrated against a highly purified recombinant human TWEAK produced at MultiSciences.

SAMPLE VALUES

Serum/Plasma - Thirty samples from apparently healthy volunteers were evaluated for the presence of TWEAK in this assay. No medical histories were available for the donors used in this study.

Sample Matrix	Number of Samples Evaluated	Range (pg/ml)	Detectable (%)	Mean of Detectable (pg/ml)
Serum	30	27.9-286.6	100	148.6

Note: The sample range is non-physiological range. The sample range of healthy human will difference according to species, sample preparation, and testing personnel, equipment varies. The above information is only reference.

SPECIFICITY

This kit could assay both natural and recombinant human TWEAK. A panel of substances listed below were prepared at 1 ng/ml in Standard Diluent to determine cross-reactivity. Preparations of the following substances at 1 ng/ml in a mid-range rhTWEAK control to determine interference. No significant cross-reactivity or interference was observed.

Human		Mouse	Rat
4-1BB Ligand<br>4-1BB Receptor<br>BAFF<br>sCD40 Ligand<br>sFas Ligand<br>OPG<br>sRANK Ligand<br>sRANK Receptor	TNF-α<br>TNF-β<br>sTNF Receptor Type II<br>sTRAIL/Apo2L<br>sTRAIL Receptor 1<br>sTRAIL Receptor 2<br>TWEAK Receptor	sCD40 Ligand<br>sRANK Ligand<br>TNF-α	TNF-α

PLATE LAYOUT

	1	2	3	4	5	6	7	8	9	10	11	12
A	S1	S1										
B	S2	S2										
C	S3	S3										
D	S4	S4										
E	S5	S5										
F	S6	S6										
G	S7	S7										
H	Blank	Blank										

MULTI SCIENCES
联科生物

Human TSLP ELISA Kit

Catalog Number:
EK165 - 48
EK165 - 96

For the quantitative determination of human Thymic Stromal Lymphopoietin (TSLP) concentrations in cell culture supernates, serum and plasma.

This package insert must be read entirely before using this product. For proper performance, follow the protocol provided with each individual kit.

FOR RESEARCH USE ONLY. NOT FOR USE IN DIAGNOSTIC PROCEDURES.

MULTISCIENCES (LIANKE) BIOTECH CO., LTD.
3F, Building 2, No.1688 Tianma Road, Times High Tech Park,
Xiaoshan District, Hangzhou, Zhejiang Province, China.
www.multisciences.net
Tel: +86-057128874209
E-mail: info.cn@liankebio.com

TABLE OF CONTENTS

ASSAY PROCEDURE SUMMARY 1

Introduction

Description 2
Principle of the Assay 2
Limitations of the Procedure 2

General Information

Materials Provided 3
Storage 3
Other Supplies Required 4
Precaution 4
Technical Hints 5

Assay Protocol

Sample Collection and Storage 5
Reagent Preparation 6, 7
Assay Procedure 7, 8

Analysis

Calculation of Results 8
Typical Data 8
Sensitivity 9
Precision 9
Recovery 9
Linearity 9
Calibration 10
Sample Values 10
Specificity 10

ASSAY PROCEDURE SUMMARY

1. Prepare all reagents and standards as directed.

↓

2. Add 300 µl *Washing Buffer (1×)* per well to soak for about 30 seconds. Use immediately after aspirate.

↓

3. Add100 µl 2-fold diluted *Standard* in duplicate. Add 100 µl *Standard Diluent/culture medium* to Blank well.

↓

4. Serum/Plasma: Add 80 µl *Assay Buffer (1×)* and 20 µl sample to the sample well. Cell culture Supernates: Add 100 µl cell culture supernates to the sample well. Step 3 and 4 should be completed within 15 minutes. Incubate for 2 hours at RT. Aspirate and wash 6 times.

↓

5. Add 100 µl of diluted *Detect Antibody* to each well. Incubate for 2 hours at RT. Aspirate and wash 6 times.

↓

6. Add 100 µl of diluted *Streptavidin-HRP* to each well. Incubate for 45 minutes at RT. Aspirate and wash 6 times.

↓

7. Add 100 µl *Substrate Solution* to each well.

Incubate for 5 - 30 minutes at RT. Protect from light.

↓

8. Add 100 µl *Stop Solution* to each well.

↓

9. Read at 450 nm within 30 minutes. Correction 570 or 630 nm.

DESCRIPTION

Thymic stromal lymphopoietin (TSLP) is produced mainly by non-hematopoietic cells such as fibroblasts, epithelial cells and different types of stromal or stromal-like cells. It mainly impacts myeloid cells and induces the release of T cell-attracting chemokines from monocytes and enhances the maturation of myeloid (CD11c+) dendritic cells. TSLP has also been shown to activate the maturation of a specific subset of dendritic cells located within the epidermis, called Langerhans cells. Within the thymus TSLP activation of both myeloid and plasmacytoid (CD123+) dendritic cells results in the production of regulatory T cells. TSLP signals through a heterodimeric receptor complex composed of the thymic stromal lymphopoietin receptor CRLF2 and the IL-7R alpha chain. After binding STAT5 phosphorylation is induced resulting in the expression of upstream transcription factors. TSLP expression is linked to many disease states including asthma, inflammatory arthritis, atopic dermatitis, and eczema and other allergic states.

PRINCIPLE OF THE ASSAY

Human TSLP ELISA Kit is based on the quantitative sandwich enzyme-linked immunosorbent assay technique to measure concentration of human TSLP in the samples. A monoclonal antibody specific for human TSLP has been pre-coated onto a microplate. Standards and samples are pipetted into the wells and TSLP present is bound by the immobilized antibody. After washing away any unbound substances, a biotin-linked detect antibody specific for TSLP is added to the wells. Following a wash to remove any unbound antibody-biotin reagent, Streptavidin-HRP is added. After washing, substrate solution is added to the wells and color develops in proportion to the amount of TSLP bound in the initial step. The color development is stopped and the intensity of the color is measured.

LIMITATIONS OF THE PROCEDURE

- FOR RESEARCH USE ONLY. NOT FOR USE IN DIAGNOSTIC PROCEDURES.
- Do not use expired kit or reagents.
- Do not use reagents from other lots or manufacturers. Do not prepare component by yourself.
- If concentration of assayed factor in samples is higher than the highest standard, dilute the serum/plasma samples with *Assay Buffer*, dilute the cell culture supernate samples with *cell culture medium*. Reanalyze these and multiply results by the appropriate dilution factor.
- Any variation in testing personnel, sample preparation, standard dilution, pipetting technique, washing techniques, incubation time, temperature, kit age and equipment can cause variation in results.
- This assay is designed to eliminate interference by factors present in biological samples. Until all factors have been tested in the ELISA immunoassay, the possibility of interference cannot be excluded.

MATERIALS PROVIDED (96 Test)

Unopened kit should be stored at 2 - 8℃.

- **Human TSLP Microplate** (1 plate): 96-well polystyrene microplate (12 strips of 8 wells) coated with a monoclonal antibody against TSLP .
- **Human TSLP Standard** (2 vials): Recombinant TSLP in a buffered protein base with preservatives; lyophilized.
- **Human TSLP Detect Antibody** (1 vial): Biotin-conjugate anti-TSLP detect antibody; 100× liquid.
- **Standard Diluent** (1 bottle, 5 ml): In some, very rare cases, an insoluble precipitate of stabilizing protein has been seen in the Standard Diluent. This precipitate does not interfere in any way with the performance of the test and can thus be ignored.
- **Streptavidin-HRP** (1 vial): 100× liquid.
- **Assay Buffer (10×)** (1 bottle, 5 ml): PBS with 0.5 % Tween-20 and 5 % BSA.
- **Substrate** (1 bottle, 11 ml): TMB (tetramethyl-benzidine).
- **Stop Solution** (1 bottle, 11 ml): 0.18 M sulfuric acid.
- **Washing Buffer (20×)** (1 bottle, 50 ml): PBS with 1 % Tween-20.
- **Plate Covers** (6 strips).

STORAGE

Store kit reagents between 2 and 8 ℃ . Immediately after use remaining reagents should be returned to cold storage (2 to 8℃). Expiry of the kit and reagents is stated on labels.

Expiration date of the kit components can only be guaranteed if the components are stored properly, and if, in case of repeated use of one component, this reagent is not contaminated by the first handling.

<table>
<tr><td colspan="2">Unopened kit</td><td>Store at 2 - 8℃ (See expiration date on the label).</td></tr>
<tr><td rowspan="3">Opened/
Reconstituted
Reagents</td><td>1× Washing Buffer
1× Assay Buffer
Stop Solution
Standard Diluent
Substrate TMB
Detect Antibody
Streptavidin-HRP</td><td>Up to 1 month at 2 - 8℃.</td></tr>
<tr><td>Standard</td><td>Up to 1 month at ≤ -20 ℃ in a manual defrost freezer. Discard after use.</td></tr>
<tr><td>Microplate Wells</td><td>Up to 1 month at 2 - 8℃. Return unused strips to the foil pouch containing the desiccant pack, reseal along entire edge to maintain plate integrity.</td></tr>
</table>

Provided this is within the expiration date of the kit.

OTHER SUPPLIES REQUIRED

- **Microplate reader** capable of measuring absorbance at 450 nm, with correction wavelength set at 570 nm or 630 nm.
- **Pipettes and pipette tips**.
- 50 μl to 300 μl adjustable **multichannel micropipette** with disposable tips.
- Multichannel micropipette **reservoir**.
- **Beakers, flasks, cylinders** necessary for preparation of reagents.
- **Deionized or distilled water**.
- **Polypropylene** test tubes for dilution.

PRECAUTION

- All chemicals should be considered as potentially hazardous.
- We therefore recommend that this product is handled only by those persons who have been trained in laboratory techniques and that it is used in accordance with the principles of good laboratory practice. Wear suitable protective clothing such as laboratory overalls, safety glasses and gloves.
- Care should be taken to avoid contact with skin or eyes. In the case of contact with skin or eyes wash immediately with water. See material safety data sheet(s) and/or safety statement(s) for specific advice.
- The Stop Solution provided with this kit is an acid solution. Wear eyes, hand, face, and clothing protection when using this material.
- Reagents are intended for research use only and are not for use in diagnostic or therapeutic procedures.
- Do not mix or substitute reagents with those from other lots or other sources.
- Do not use kit reagents beyond expiration date on label.
- Do not expose kit reagents to strong light during storage and incubation.
- Do not eat or smoke in areas where kit reagents or samples are handled.
- Avoid contact of skin or mucous membranes with kit reagents or specimens.
- Rubber or disposable latex gloves should be worn while handling kit reagents or specimens.
- Avoid contact of substrate solution with oxidizing agents and metal.
- Avoid splashing or generation of aerosols.
- In order to avoid microbial contamination or cross- contamination of reagents or specimens which may invalidate the test use disposable pipette tips and/or pipettes.
- Use clean, dedicated reagent trays for dispensing the conjugate and substrate reagent.
- Exposure to acid inactivates the HRP and antibody conjugate.
- Glass-distilled water or deionized water must be used for reagent preparation.
- Substrate solution must be warmed to room temperature prior to use.
- Decontaminate and dispose specimens and all potentially contaminated materials as they could contain infectious agents. The preferred method of decontamination is autoclaving for a minimum of 1 hour at 121.5℃.
- Liquid wastes not containing acid and neutralized waste may be mixed with sodium hypochlorite in volumes such that the final mixture contains 1.0 % sodium hypochlorite. Allow 30 minutes for effective decontamination. Liquid waste containing acid must be neutralized prior to the addition of sodium hypochlorite.
- In some cases, an insoluble precipitate of stabilizing protein has been seen in the Standard Diluent. This precipitate does not interfere in any way with the performance of the test and can thus be ignored. Or remove precipitate by centrifuging at 6,000 × g for 5 minutes.

TECHNICAL HINTS

- When mixing or reconstituting protein solutions, always avoid foaming.
- To avoid cross-contamination, change pipette tips between additions of each standard level, between sample additions, and between reagent additions. Also, use separate reservoirs for each reagent.
- When using an automated plate washer, adding a 30 seconds soak period before washing step and/or rotating the plate between wash steps may improve assay precision.
- To ensure accurate results, proper adhesion of plate sealers during incubation steps is necessary.
- Substrate Solution should remain colorless until added to the plate. Keep Substrate Solution protected from light. Substrate Solution should change from colorless to gradations of blue.
- Stop Solution should be added to the plate in the same order as the Substrate Solution.
- The color developed in the wells will turn from blue to yellow upon addition of the Stop Solution. Wells that are green in color indicate that the Stop Solution has not mixed thoroughly with the Substrate Solution.
- It is recommended that all samples and standards be assayed in duplicate.
- Take care not to scratch the inner surface of the microwells.

SAMPLE COLLECTION AND STORAGE

Cell Culture Supernates – Remove particulates by centrifugation at 300 × g for 10 minutes and assay immediately or aliquot and store samples at ≤ -20℃.

Serum – Use a serum separator tube (SST) and allow samples to clot for 30 minutes before centrifugation for 10 minutes at 1,000 × g. Remove serum and assay immediately or aliquot and store samples at ≤ -20℃.

Plasma – Collect plasma using EDTA, citrate or heparin as anticoagulant. Centrifuge at 1,000 × g within 30 minutes of collection. Assay immediately or aliquot and store samples at ≤ -20℃.

Other biological samples might be suitable for use in the assay. Cell culture supernates, serum and plasma were tested with this assay.

Note: Samples containing a visible precipitate must be clarified prior to use in the assay. Do not use grossly hemolyzed or lipemic specimens.
If samples are to be run within 24 hours, they may be stored at 2 to 8℃. For longer storage, aliquot samples and store frozen at -20℃ to avoid loss of bioactive human TSLP.. Avoid repeated freeze-thaw cycles.

REAGENT PREPARATION

Bring all reagents and samples to room temperature before use.
If crystals form in the Buffer Concentrates, warm and gently stir them until completely dissolved.

Washing Buffer (1×)

Pour entire contents (50 ml) of the **Washing Buffer (20×)** into a clean 1,000 ml graduated cylinder. Bring to final volume of 1,000 ml with pure or deionized water.
Mix gently to avoid foaming.
Transfer to a clean wash bottle and store at 2 to 25℃. Washing Buffer (1×) is stable for 30 days.

Assay Buffer (1×)

Pour the entire contents (5 ml) of the **Assay Buffer (10×)** into a clean 100 ml graduated cylinder. Bring to final volume of 50 ml with distilled water. Mix gently to avoid foaming.
Store at 2 to 8℃. Assay Buffer (1×) is stable for 30 days.

Detect Antibody

Mix well prior to making dilutions.
Make a **1：100** dilution of the concentrated **Detect Antibody** solution with Assay Buffer (1×) in a clean plastic tube as needed.
The diluted Detect Antibody should be used within 30 minutes after dilution.

Streptavidin-HRP

Mix well prior to making dilutions.
Make a **1：100** dilution of the concentrated **Streptavidin-HRP** solution with Assay Buffer (1×) in a clean plastic tube as needed.
The diluted Streptavidin-HRP should be used within 30 minutes after dilution.

Sample Dilution

If your samples have high TSLP content, dilute serum/plasma samples with Assay Buffer (1×). For cell culture supernates, dilute with cell culture medium.

Human TSLP Standard

Reconstitute **Human TSLP Standard** by addition of distilled water. Reconstitution volume is stated on the label of the standard vial. Swirl or mix gently to insure complete and homogeneous solubilization (concentration of reconstituted standard = 2,000 pg/ml).
Allow the standard to reconstitute for 10 - 30 minutes. Mix well prior to making dilutions.
Use polypropylene tubes.

For serum/plasma samples, mixing *concentrated human TSLP standard* (230 μl) with 230 μl of *Standard Diluent* creates the high standard (1,000 pg/ml). Pipette 230 μl of *Standard Diluent* into each tube. Use the high standard to produce a 1:1 dilution series (scheme below). Mix each tube thoroughly before the next transfer. *Standard Diluent* serves as the zero standard (0 pg/ml).

For cell culture supernates, mixing *concentrated human TSLP standard* (230 μl) with 230 μl of cell culture medium creates the high standard (1,000 pg/ml). Pipette 230 μl of cell culture medium into each tube. Use the high standard to produce a 1:1 dilution series. Mix each tube thoroughly before the next transfer. Cell culture medium serves as the zero standard (0 pg/ml).

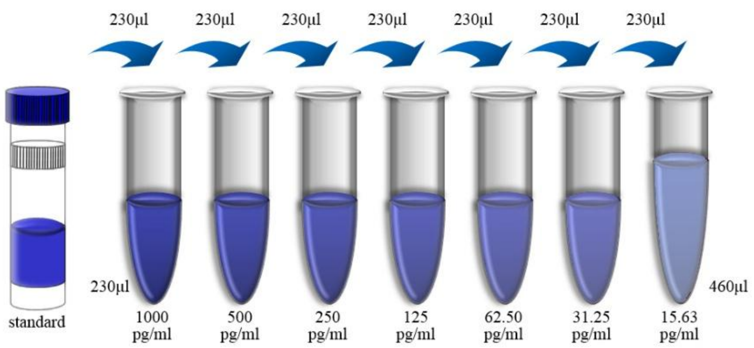

ASSAY PROCEDURE

Bring all reagents and samples to room temperature before use.

1. Prepare all reagents including microplate, samples, standards and working solution as described in the previous sections.
2. Remove excess microplate strips and return them to the foil pouch containing the desiccant pack, and reseal for further use.
3. Add 300 μl *Washing Buffer (1×)* per well, and allow it for about 30 seconds before aspiration. Soaking is highly recommended to obtain a good test performance. Empty wells and tap microwell strips on absorbent pad or paper towel to remove excess *Washing Buffer (1×)*. Use the microwell strips immediately after washing. **Do not allow wells to dry.**
4. Add 100 μl 2-fold diluted Standard to Standard well. Add 100 μl *Standard Diluent / culture medium* to Blank well.
5. Serum/Plasma: Add 80 μl *Assay Buffer (1×)* and 20 μl sample to the sample well. Cell culture supernates: add 100 μl cell culture supernates to the sample well. Ensure reagent addition in step 4 and 5 is uninterrupted and completed within 15 minutes.
6. Seal the plate with an *adhesive film*. Incubate at room temperature (18 to 25℃) for 2 hours on a microplate shaker set at 300 rpm.
7. Aspirate each well and wash by filling each well with 300 μl *Washing Buffer (1×)*, repeat five times for a total six washes. Complete removal of liquid at each step is essential to the best performance. After the last wash, remove any remaining *Washing Buffer (1×)* by aspirating or decanting. Invert the plate and tap it against clean paper towels.
8. Add 100 μl of diluted Detect Antibody to each well.
9. Seal the plate with an adhesive film. Incubate at room temperature (18 to 25℃) for 2 hours on a microplate shaker set at 300 rpm.
10. Repeat aspiration/wash as in step 7.
11. Add 100 μl of diluted Streptavidin-HRP to each well.
12. Seal the plate with a fresh adhesive film. Incubate at room temperature (18 to 25℃) for 45 minutes on a microplate shaker set at 300 rpm.
13. Repeat aspiration/wash as in step 7.
14. Add 100 μl of Substrate Solution to each well. Incubate for 5 - 30 minutes at room temperature. Protect from light.
15. Add 100 μl of Stop Solution to each well. The color will turn yellow. If the color in the well is green or if the color change does not appear uniform, gently tap the plate to ensure thorough

mixing.

16. Measure the optical density value within 30 minutes by microplate reader set to 450 nm. If wavelength correction is available, set to 570 nm or 630 nm. If wavelength correction is not available, subtract readings at 570 nm or 630 nm from the readings at 450 nm. This subtraction will correct for optical imperfections in the plate. Reading directly at 450 nm without correction may generate higher concentration than true value.

CALCULATION OF RESULTS

Average the duplicate optical density readings for each standards and sample, then subtract the average optical density value of the zero standard.

Standard Concentration as horizontal axis, optical density (OD) Value as the vertical axis, regressing the data and create a standard curve using computer software. The data may be linearized by plotting the log of the TSLP concentrations versus the log of the OD and the best fit line can be determined by regression analysis. This procedure will produce an adequate but less precise fit of the data.

Note: The finally concentration of top standard is 1,000 pg/ml.
If samples have been diluted following the instruction, the final dilution factor is 5. If sample have been diluted by other means, the concentration read from the standard curve must be multiplied by the appropriate dilution factor.

TYPICAL DATA

A standard curve must be run within each assay. This standard curve is provided for demonstration only.

pg/ml	O.D.		Average	Corrected
0.00	0.054	0.055	0.055	
15.63	0.110	0.114	0.112	0.058
31.25	0.149	0.156	0.153	0.098
62.50	0.242	0.252	0.247	0.193
125.00	0.407	0.421	0.414	0.360
250.00	0.714	0.734	0.724	0.670
500.00	1.219	1.185	1.202	1.147
1000.00	1.801	1.852	1.827	1.772

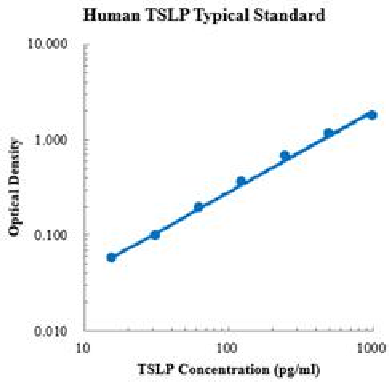

SENSITIVITY

The minimum detectable dose (MDD) of TSLP is typically about 0.13 pg/ml.(mean of 6 independent assays).
The MDD was determined by adding two standard deviations to the mean optical density value of ten zero standard replicates and calculating the corresponding concentration.

PRECISION

Intra-assay Precision (Precision within an assay)
Three serum-based and buffer-based samples of known concentration were tested twenty times on one plate to assess intra-assay precision.

Inter-assay Precision (Precision between assays)
Three serum-based and buffer-based samples of known concentration were tested in six separate assays to assess inter-assay precision.

	Intra-assay precision				Inter-assay precision		
**Sample**	1	2	3		1	2	3
**n**	20	20	20		6	6	6
**Mean (pg/ml)**	48.3	186.6	715.8		47.0	174.7	696.3
**Standard deviation**	1.6	7.7	26.5		2.7	13.1	36.2
**CV (%)**	3.3	4.1	3.7		5.8	7.5	5.2

RECOVERY

The spike recovery was evaluated by spiking 3 levels of human TSLP into five health human serum samples. The un-spiked serum was used as blank in these experiments.
The recovery ranged from 85 % to 118 % with an overall mean recovery of 100 %.

LINEARITY

To assess the linearity of the assay, five samples were spiked with high concentration of TSLP in human serum and diluted with Standard Diluent to produce samples with values within the dynamic range of the assay.

	Average (%)	Range (%)
**1:2**	101	91 - 110
**1:4**	103	95 - 101
**1:8**	96	90 - 109
**1:16**	99	95 - 108

CALIBRATION

This immunoassay is calibrated against a highly purified human TSLP produced at MultiSciences.

SAMPLE VALUES

Serum/Plasma - Thirty samples from apparently healthy volunteers were evaluated for the presence of TSLP in this assay. No medical histories were available for the donors used in this study.

Sample Matrix	Number of Samples Evaluated	Range (pg/ml)	Detectable (%)	Mean of Detectable (pg/ml)
Serum	30	n.d. - 64.4	3	64.4

n.d. = non-detectable. Samples measured below the sensitivity are considered to be non-detectable.

Note: The sample range is non-physiological range. The sample range of healthy human will difference according to species, sample preparation, and testing personnel, equipment varies. The above information is only reference.

SPECIFICITY

This kit could assay both natural and recombinant human TSLP. A panel of substances listed below were prepared at 1 ng/ml in Standard Diluent to determine cross-reactivity. Preparations of the following substances at 1 ng/ml in a mid-range rhTSLP control to determine interference. No significant cross-reactivity or interference was observed.

Human		Mouse	Rat
IFN-γ	IL-17A	GM-CSF	IFN-γ
IL-1β	IL-18	IFN-γ	IL-1β
IL-2	IL-21	IL-1β	IL-4
IL-4	IL-22	IL-2	IL-6
IL-5	IL-23	IL-4	IL-10
IL-6	MCP-1	IL-6	TNF-α
IL-8	TGF-β1	IL-10	
IL-10	TNF-α	TNF-α	
IL-12	VEGF		

PLATE LAYOUT

	1	2	3	4	5	6	7	8	9	10	11	12
A	S1	S1										
B	S2	S2										
C	S3	S3										
D	S4	S4										
E	S5	S5										
F	S6	S6										
G	S7	S7										
H	Blank	Blank										

Beyotime

Support: +86-400-1683301 / info-intl@beyotime.com
Orders: order-intl@beyotime.com
Tel: +86-400-1683301 / +86-800-8283301
Add: 1500 Xinfei Rd, Songjiang, Shanghai, China
Web: www.beyotime.com

Human CCL19/MIP-3β ELISA Kit

Cat. No.	Product Name	Pack Size
PC123	Human CCL19/MIP-3β ELISA Kit	96T

Description:

- Beyotime's Human CCL19/MIP-3β ELISA Kit (Human Macrophage Inflammatory Protein-3β Enzyme-Linked ImmunoSorbent Assay Kit) provides a specific and highly sensitive method for quantification of CCL19/MIP-3β in human serum, plasma, and cell culture supernatant.
- This product has high detection sensitivity, high specificity and good reproducibility. The multiple repeated assays show that the minimum detection amount is 14.5 pg/ml and there is no cross-reactivity with human HCC-4 and MIP-3. The intra-plate and inter-plate coefficients of variation are both less than 10%.
- Macrophage Inflammatory Protein-3β (MIP-3β), also known as CCL19 (Chemokine C-C motif Ligand 19) and ELC (EBI1-Ligand Chemokine), is an important chemokine expressed mainly in secondary lymphoid tissues. *CCL19* gene encodes a 98 amino acid precursor protein containing a 21 amino acid signal peptide and a 77 amino acid mature CCL19 peptide fragment. CCL19 is predominantly expressed in lymph nodes and fibroblastic reticulocytes and, like CCL21, acts on chemokine receptor 7 (CCR7). CCL19 and CCL21 share 32% amino acid homology and structurally, CCL21 has 32 more amino acids at the C-terminus than CCL19 to regulate CCL21 binding to other molecules. Both have approximately the same affinity as CCR7, but produce different effects. CCL19 expression is elevated to some extent in conditions such as atherosclerosis, tumors, bone disease, asthma, HIV infection, and pneumonia.
- Although CCL19 binds to some cell surface receptors such as CCRL12 (C-C motif Receptor Like 2) and ACKR4 (Atypical Chemokine Receptor 4), its function is mainly regulated by the CCR7 receptor, a G protein-coupled receptor of subtype A. The binding of CCL19 to the CCR7 receptor promotes the migration, activation and survival of dendritic cells, T cells, eosinophils, B cells, endothelial cells and different species of tumor cells.
- This kit employs the double-antibody sandwich ELISA for quantification analysis of human CCL19/MIP-3β in samples (Figure 1). The monoclonal antibodies against human CCL19/MIP-3β (capture antibody) are precoated on the plate and when a standard or sample is added, the human CCL19/MIP-3β binds to the capture antibody. The biotin-conjugated human CCL19/MIP-3β antibody is then added and binds to human CCL19/MIP-3β on the plate to form a sandwiched immune complex. After that, HRP-labeled Streptavidin is added and binds to the sandwich immune complex through the specific interaction between biotin and streptavidin. Finally, the chromogenic reaction is initiated by the addition of TMB Solution. TMB produces a deep blue color during the enzymatic degradation of hydrogen peroxide by HRP, and the addition of Stop Solution gives a clear yellow color that absorbs at 450nm. The A450 value is directly proportional to the concentration of human CCL19/MIP-3β in samples which can be calculated from the standard curve generated in the same assay.

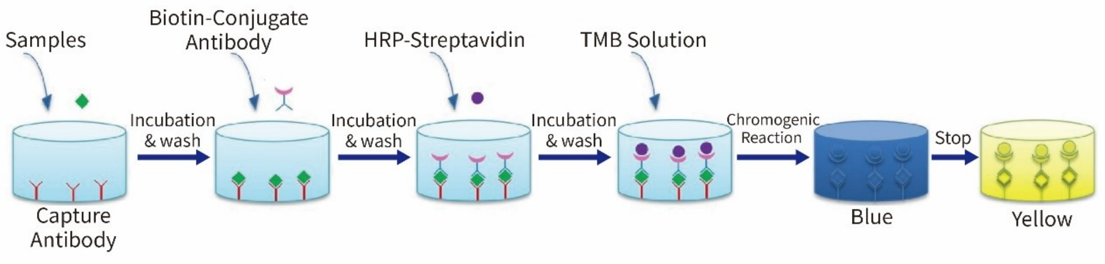

Figure 1. Schematic diagram of double-antibody sandwich ELISA

- This kit is sufficient for 96 assays.

Packing List:

Item	Component	Quantity
PC123-1	96-well Strip-well Plate Coated with Human CCL19/MIP-3β Antibody	8 wells × 12 strips
PC123-2	Assay Buffer	5ml
PC123-3	Standard Dilution Buffer	10ml
PC123-4	Human CCL19/MIP-3β Standard	2-4 bottles
PC123-5	Biotin-conjugated Human CCL19/MIP-3β Antibody	10ml
PC123-6	HRP-labeled Streptavidin	10ml
PC123-7	Wash Buffer (20X)	30ml
PC123-8	TMB Solution	10ml
PC123-9	Stop Solution	5ml
PC123-10	Adhesive Films (transparent)	2
PC123-11	Adhesive Films (white)	2
Manual	—	1 copy

Storage Conditions:

Store the Standard at 4ºC for up to 1-2 weeks, or -20ºC for up to 6 months. Store the other components in this kit at 4ºC for up to 6 months.

Precautions:

- The standard is generally lyophilized powder. Check the instructions labeled on the standard vial to prepare the standard solution.
- Crystal precipitation may exist in Wash Buffer (20X) at low temperatures. Please dissolve it completely using a water bath at room temperature prior to use.
- Standard solution should be prepared freshly. Discard the rest after use.
- Avoid oxidizer and metal contamination that cause the invalidation of TMB Solution.
- Change pipette tips between different samples and liquids to prevent contamination and incorrect loading volumes.
- Do not mix or interchange reagents from different kit lots.
- It is particularly important to perform sufficient mixing of reactions to ensure an accurate result. Please shake the 96-well plate gently after the addition of reagents.
- Most procedures of this experiment should be performed at room temperature (25-28ºC). Temperature lower than 25ºC will result in a significant decrease in the absorbance value of reactions.
- The washing process is very important. Insufficient wash will result in reduced accuracy and increased experimental errors.
- Run all standards, controls, and samples in duplicate.
- Avoid the formation of air bubbles when pipetting.
- This product is for R&D only. Not for drug, household, or other uses.
- For your safety and health, please wear a lab coat and disposable gloves during the operation.

Instructions for Use:

1. **Preparation of the sample.**

   a. **Cell supernatant:** Centrifuge cell cultures at 100-500×g for 5 minutes to collect the supernatant.

   b. **Serum:** Leave the whole blood undisturbed at room temperature for 30 minutes to 2 hours. After the whole blood clots, collect the yellow supernatant (serum) by centrifuging the whole blood at 1000-2000×g for 10 minutes at 4ºC, and keep the serum on ice.

   Note: *Do Not add any preservatives or anticoagulants to serum.*

   c. **Plasma:** Add heparin or EDTA anticoagulant to whole blood and place on ice after mix. After centrifuging at 1000-2000×g for

10 minutes at 4ºC, collect the yellow supernatant (plasma) and keep it on ice.

Note 1: *If the samples cannot be analyzed immediately, make aliquots and store them at -20ºC or -80ºC. Avoid repeated freeze-thaw.*

Note 2: *Samples should be clear and transparent. Remove any particulates from samples by centrifuging before being analyzed.*

Note 3: *Do not use hemolyzed, hyperlipidemia, or contaminated samples for analysis.*

Note 4: *Serum or plasma samples may need to be properly diluted with Assay Buffer before the assay.*

2. **Preparation of the kit.**

a. Thaw the reagents and equilibrate to room temperature (25-28ºC) prior to use. Store the reagents at 4ºC immediately after use.

b. Prepare an appropriate amount of 1X Wash Buffer by diluting the Wash Buffer (20X) with ddH_2O.

c. Reconstitute Standard to 1000pg/ml with Standard Dilution Buffer according to the instructions labeled on the standard vial. Mix gently and incubate for 15min at room temperature. Gently pipette the contents several times to dissolve the standard completely. Generally, each concentration of standard is analyzed at least in duplicates (at least 2 wells) and 100μl is needed for each well. If one bottle of Standard is not enough, use more bottles of Standard, but the contents from different bottles need to be mixed before performing dilutions.

d. Add 250μl of Standard Dilution Buffer to each of 5 tubes and make serial dilutions as shown in Figure 2 to obtain 5 dilutions: 1/2 (500pg/ml), 1/4 (250pg/ml), 1/8 (125pg/ml), 1/16 (62.5pg/ml), 1/32 (31.25pg/ml). Mix thoroughly between steps.

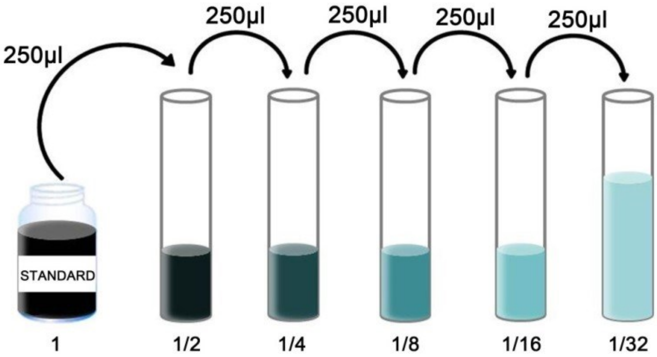

Figure 2. Dilution diagram for preparation of different concentrations of Standard.

3. **Perform ELISA.**

a. Determine the number of pre-coated 8-well strips required for the experiment. Insert the strips in a frame for use. Re-bag any unused strips and store them at 4ºC.

b. Prepare the Standard freshly and plot a standard curve for every run. Set up the blank control by only adding TMB Solution and Stop Solution.

c. Add 100μl of samples or standards to each well. Cover wells with Adhesive Films (transparent), and incubate for 2 hours at room temperature.

Note: *For the assay of CCL19/MIP-3β in serum or plasma samples, add 50 μl of Assay Buffer followed by 50 μl of sample. At this point, the sample is diluted 2 times. If the sample concentration is too high and beyond the detection range, pleas add 50 μl of Assay Buffer at first, and then add 50 μl of the sample diluted with Standard Dilution Buffer. It is necessary to record the dilution factor of the sample.*

d. Thoroughly aspirate the solution by gently lowering a pipette tip into the bottom of each well and wash wells 5 times with 300μl 1X Wash Buffer. Allow the buffer to stand for 15-30 seconds before aspiration. After the last wash, invert the strip and tap dry on absorbent tissue.

e. Add 100μl of Biotin-conjugated Antibody to each well (***Note:*** *the antibody can be used directly without dilution*). Cover wells with Adhesive Films (transparent) and incubate for 1 hour at room temperature.

f. Thoroughly aspirate the solution and wash wells 5 times with 300μl of 1X Wash Buffer, as described in step 3d.

g. Add 100μl of HRP-labeled Streptavidin to each well (***Note:*** *the HRP-labeled Streptavidin can be used directly without dilution*), cover wells with Adhesive Films (white), and incubate at room temperature for 20 minutes in the dark. If the room temperature is low, prolong the incubation time as appropriate.

h. Thoroughly aspirate the solution and wash wells 5 times with 300μl of 1X Wash Buffer, as described in step 3d.

i. Add 100μl of TMB Solution to each well, cover wells with Adhesive Films (white), and incubate at room temperature for 15-20

minutes in the dark. If the room temperature is low, prolong the incubation time until the standard sample exhibits significant color change. If the concentration of target protein in sample is high, color change will occur soon.

j. Add 50μl of Stop Solution to each well. Read the absorbance at 450nm immediately after mixing.

4. Analysis.

a. Calculate the average A450 value for each standard and sample. Duplicates should be within 20 percent of the mean value.

b. Subtract the A450 value of blank control from the A450 values of standards and samples (This step can be omitted if there is no blank control).

c. Generate the standard curve by plotting the concentrations of standard on the abscissa and their corresponding A450 values on the ordinate (Figure 3).

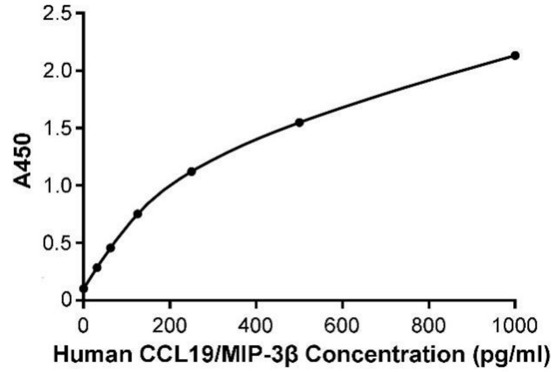

Figure 3. The standard curve of Beyotime's Human CCL19/MIP-3β ELISA Kit (PC123). This figure is for reference only, which may vary due to different experimental conditions.

d. Determine the concentrations of human CCL19/MIP-3β in samples from the standard curve based on their A450 values.

Note: *Dilute samples producing signals greater than the upper limit of the standard curve in Standard Dilution Buffer and reanalyze. Multiply the concentration by the dilution factor.*

Related Products:

Cat. No.	Product Name	Pack Size
PC123	Human CCL19/MIP-3β ELISA Kit	96T
PC130	Human CCL2/MCP-1 ELISA Kit	96T
PC138	Human CCL21/6Ckine ELISA Kit	96T
PC145	Human CCL3/MIP-1α ELISA Kit	96T
PC153	Human CCL4/MIP-1β ELISA Kit	96T
PC160	Human CCL5/RANTES ELISA Kit	96T

Version 2018.11.20
